# Synthesis and Hemolytic Activity of Bile Acid-Indole Bioconjugates Linked by Triazole

**DOI:** 10.1021/acs.joc.3c00815

**Published:** 2023-12-07

**Authors:** Natalia Berdzik, Hanna Koenig, Lucyna Mrówczyńska, Damian Nowak, Beata Jasiewicz, Tomasz Pospieszny

**Affiliations:** † Department of Bioactive Products, Faculty of Chemistry, Adam Mickiewicz University, Uniwersytetu Poznańskiego 8, 61-614 Poznań, Poland; ‡ Department of Cell Biology, Faculty of Biology, Adam Mickiewicz University, Uniwersytetu Poznańskiego 6, 61-614 Poznań, Poland; § Department of Quantum Chemistry, Faculty of Chemistry, Adam Mickiewicz University in Poznan, Uniwersytetu Poznanskiego 8, 61-614 Poznań, Poland

## Abstract

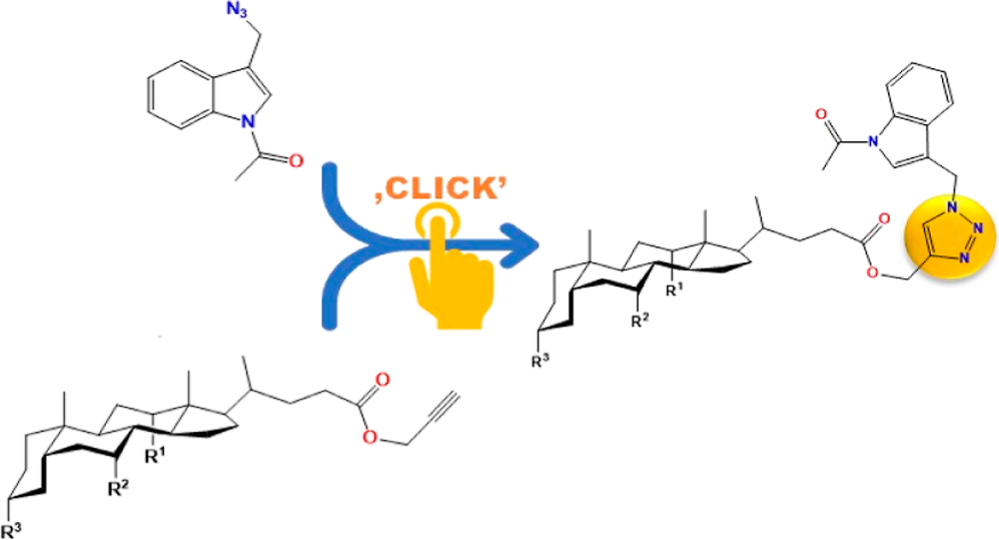

New formyl and acetyl derivatives of bile acid propargyl esters and their bioconjugates with modified gramine molecules have been obtained using the click chemistry method to study their hemolytic potency. The structures of all compounds were confirmed by spectral (^1^H- and ^13^C NMR and FT-IR) analysis and mass spectrometry (ESI-MS) as well as PM5 semiempirical methods. According to the results, the structural modification of formyl and acetyl bile acid derivatives, leading to the formation of new propargyl esters and indole bioconjugates, reduces their hemolytic activity. According to molecular docking studies, the tested ligands are highly likely to exhibit a similar affinity, as native ligands, for the active sites of specific protein domains (PDB IDs: 2Q85 and 5V5Z). The obtained results may be helpful for the development of selective bile acid bioconjugates as effective antibacterial, antifungal, or antioxidant agents.

## 1 Introduction

Natural products represent many chemical compounds synthesized by living organisms that play a significant role in different physiological processes. Steroids and alkaloids are important natural products.^1^ Among steroids, cholesterol is a crucial component of the animal cell membrane^2−8^ and a substrate for synthesizing vitamin D, bile acid, and steroid hormones.^1−4^ Steroids vary in the presence of different functional groups. Due to their biological significance and chemical properties, steroids and their derivatives are widely used in biomimetic chemistry, host––guest chemistry, and pharmacology.^9,10^ As structural building blocks, bile acids are used in constructing molecular receptors, and their dimers are employed in developing macrocyclic artificial receptors that exhibit good organogellation properties.^11−14^

Among the alkaloids, the group that gained much interest is indole alkaloids. Reserpine is known for its antipsychotic and antihypertensive activity. Vincristine and vinblastine are used for cancer treatment. Harmine, Harman, and harmaline exhibit antidepressant, antiplatelet, and spasmolytic activity.^15−18^ Gramine, [1-(1*H*-indol-3-yl)-*N*,*N*-dimethylmethanamine], the best-known indole alkaloid, is often used as an initial compound in synthesizing various biologically active substituted indoles. This tertiary amine and its derivatives have broad biological activities such as antiviral, antibacterial, antioxidant, and anticancer. They also play an essential role in amino acid metabolism and affect redox processes.^19−21^

Bioconjugates are compounds containing at least one biomolecule. Among the most known steroid-polyamine bioconjugates is squalamine, an amino sterol antibiotic.^22,23^ Squalamine has been isolated from liver cells of spiny dogfish (*Squalus acanthias*),,^24^ and its activity against Gram-positive and Gram-negative bacteria, protozoa, fungi, and viruses, including HIV, has been confirmed.^22^

Bioconjugates of indole, such as indole-phthalimide or indole-uracil, exhibit high cytoprotective effects against AAPH-induced oxidative hemolysis.^25,26^ Some indole-containing pyrazole analogs showed significant activity against leukemia, whereas substituted-3-(4,5-diphenyl-1*H*-imidazol-2-yl)-1*H*-indole derivatives act as an antibacterial agent.^27,28^

In recent years, compounds containing a triazole ring have gained more attention. Triazoles are attractive pharmacophores because they resist oxidation, reduction, and hydrolysis.^29−32^ The 1,2,3-triazole five-membered heterocyclic ring can form inter- or intramolecular hydrogen bonds and dipole–dipole interactions, increasing product solubility and ability to bind other molecules.^33^

Owing to the importance of both the indole compounds and bile acids and as a continuation of our interest in alkaloid and steroid modification, in this work, we report the synthesis and structural analysis of bile acids-indole conjugates linked by triazole ring.

It is well-known that bile acids as amphiphilic molecules possess hemolytic potency dependent on their chemical structure and concentration. The molecular mechanism of interaction between bile acids molecules and the cell membrane, as well as the hemolysis type, is dependent on the protonation of the anionic group of bile acids.^34,35^

Based on the fact that the hemolytic activity of bile acids and their formyl and acetyl derivatives can be reduced or eliminated by the introduction of gramine,^36^ the newly synthesized compounds have also been evaluated for their hemolytic potency.

Considering that both bile acid derivatives and indole compounds could be efficient antibacterial and fungicidal agents, we studied their potent antimicrobial activity with a molecular docking methodology. Molecular docking can inform us whether our ligand will fit the protein’s active site and what the binding energy might be. The lower the energy level, the more stable the ligand–receptor complex.^37,38^ Moreover, molecular docking studies can reveal possible interactions between a tiny molecule, the ligand, and a macromolecular target, in this instance, a protein domain.

## 2 Results and Discussion

### 2.1 Synthesis and Spectroscopic Characterization

**Figure 1:**
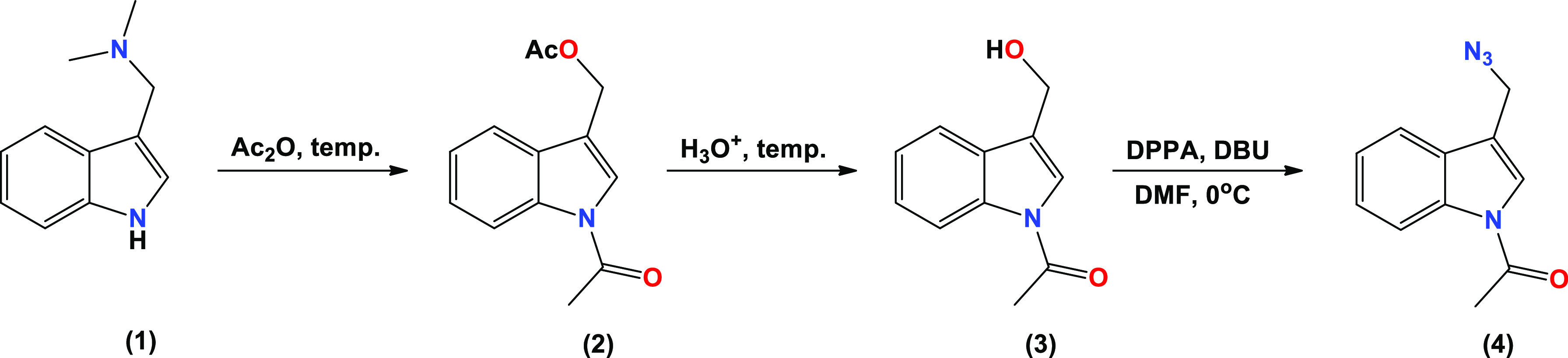
Synthesis of *N*-acetyl-3-azidemethylindole (**4**).

The synthesis of *N*-acetyl-3-azidomethylindole (**4**) is shown in Figure 1. Compound (**4**) was synthesized in good yield from gramine (**1**) by three successive reactions: acylation, hydrolysis, and nucleophilic substitution. In the final experiment, *N*-acetyl-3-hydroxymethylindole and diphenylphosphoryl azide (DPPA) was dissolved in DMF with a catalytic amount of DBU at 0 °C. A mixture of crude bioconjugates was obtained and then separated by column chromatography. The bile acid propargyl esters were obtained by reaction of propargyl bile acids with HCOOH (**8**–**10**) or Ac_2_O (**12**–**14**) with satisfactory yields except for compound (**11**), a byproduct with extremely low efficiency.

**Figure 2:**
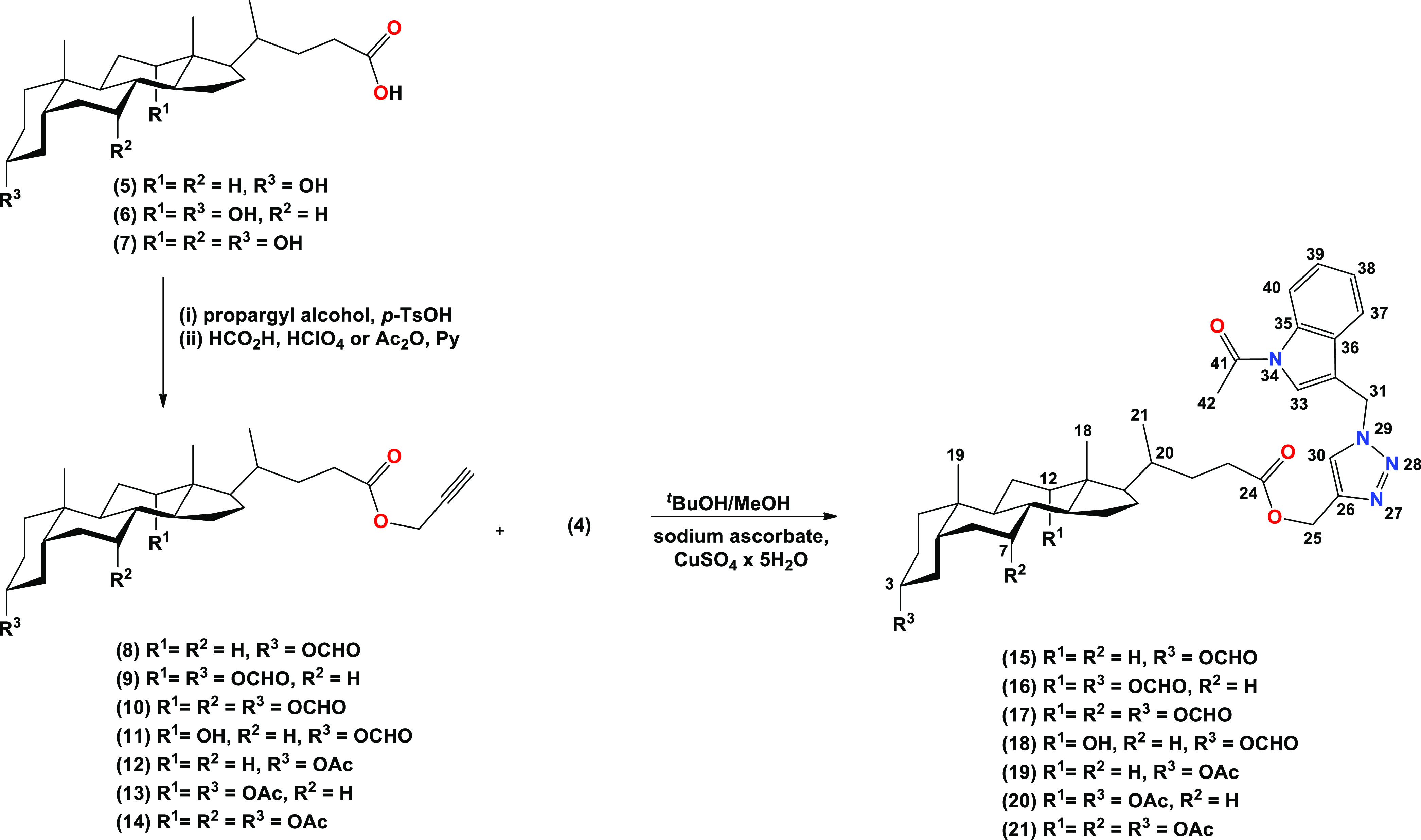
Synthesis of bile-acids propargyl esters (**8**–**14**) and new 1,2,3-triazole ring bioconjugates (**15**–**21**) of bile acid derivatives and gramine derivative.

The synthesis of new bioconjugates of bile acids (lithocholic, LCA; deoxycholic, DCA; cholic, CA) and gramine is presented in Figure 2. The reaction of propargyl esters of bile acid formyl derivatives (**8**–**14**) and the azide derivative of gramine (**4**) in the presence of CuSO_4_·5H_2_O and sodium ascorbate in *t*-BuOH/MeOH (5:1) gave products (**15**–**21**) in good yields.

**Figure 3:**
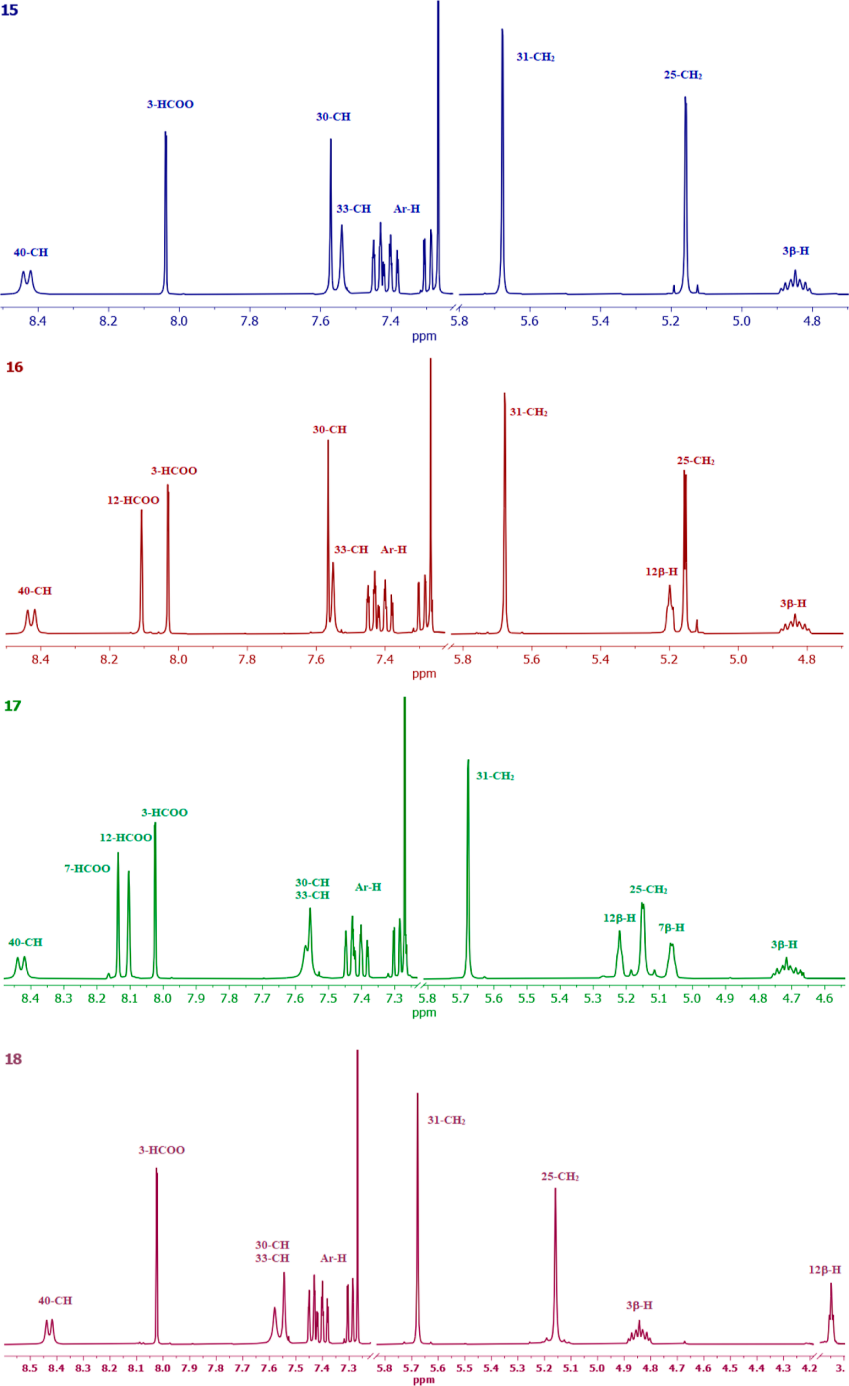
^1^H NMR Spectra in the region of 3.90–8.50 ppm for the most characteristic signals of compounds (**15**–**18**).

The most characteristic signals of representative compounds (**15**–**18**) in the range of 3.90–8.50 ppm in the ^1^H NMR spectra are shown in Figure 3. In the ^1^H NMR spectrum of propargyl 3α-formyl-5β-cholan-24-oate (**8**), propargyl 3α,12α-diformyl-5β-cholan-24-oate (**9**), propargyl 3α,7α,12α-triformyl-5β-cholan-24-oate (**10**), propargyl 3α-formyl-12α-hydroxy-5β-cholan-24-oate (**11**), and 3α-acetyl-5β-cholan-24-oate (**12**), characteristic two hydrogen singlets in the range 0.76–0.64 and 0.95–0.93 ppm and a doublet at 0.98–0.83 ppm assigned to CH_3_-18, CH_3_-19, and CH_3_-21, respectively, are present. The protons of the 3α-OCHO group gave signals in the range 8.04–8.02 ppm for all bioconjugates (**8**–**11**) and additionally 8.11 ppm for the 7α-OCHO group in compound (**10**), and the 12α-OCHO group gave signals in the range of 8.16–8.14 ppm for compounds (**9**) and (**10**). For compound (**12**), the signal at 2.03 ppm corresponds to the protons of the 3α-OCOCH_3_ group. All compounds show characteristic multiplets associated with the axial positions of the C3β–H protons in the steroid skeleton in the range of 4.94–4.67 ppm. In the spectrum of compounds (**10**) and (**11**), additional signals correspond to the C12β–H proton at 5.27 and 3.99 ppm, respectively. For compound **10**, the C7β–H protons appear at 5.07 ppm. All bile acids–indole conjugates with 1,2,3-triazole ring (**15**–**21**) in CDCl_3_ show diagnostic proton signals C30–H at 7.58–7.57 ppm. The formyl derivatives (**15**–**18**) show additional signals at 8.11–8.02 assigned to the proton of the formyloxy groups. Signals diagnostic for the aromatic ring of the indole moiety appear at the range of 8.43–7.28 ppm for all new bioconjugates. The protons of the methylene groups C25–H and C31–H linked directly to the triazole ring give signals at about 5.16–5.15 ppm and 5.69–5.68 ppm, respectively. The diagnostic protons CH_3_ from the acetyl groups of compounds (**19**–**21**) and the *N*-acetoxy group of all new bioconjugates are found at 2.12–2.03 and 2.66 ppm, respectively. ^1^H NMR spectra of bioconjugates (**15**–**21**) show characteristic multiplets of protons of C3β–H from the bile acid skeleton, ranging from 4.89 to 4.53 ppm. The characteristic signals of C12β–H protons are observed at 5.22–5.04 ppm for compounds (**16**, **17**, **20**, **21**) and 3.95 ppm for compound (**18**). Signals assigned to the C7β–H protons of the bile acid skeleton occur as doublets at 5.06 and 4.90 ppm for bioconjugates (**17**) and (**21**), respectively. In the bile acids skeleton, two hydrogen singlets ranking from 0.69 to 0.59 and 0.94–0.90 ppm and a characteristic doublet at 0.90–0.74 ppm were assigned to CH_3_-18, CH_3_-19, and CH_3_-21, respectively.

The ^13^C NMR spectra of substrates (**8**–**14**) and bioconjugates (**15**–**21**) show characteristic signals at 12.71–11.94, 23.07–20.78, and 18.24–17.23 ppm, which are assigned to CH_3_-18, CH_3_-19, and CH_3_-21, respectively. For all newly obtained acetyl derivatives (**12**) and (**19**–**21**), signals from the C=O carbon atom occur at 170.66–170.52 ppm (3α-OCOCH_3_), 170.48–170.44 ppm (12α-OCOCH_3_), and 170.36 ppm (7α-OCOCH_3_). However, for the new formyl derivatives (**15**–**18**), carbon atoms in positions 3α of formyloxy groups resonate at 160.82–160.46 ppm. The carbon atoms of the C(12)=O steroid skeleton gave signals in the range of 160.67–160.47 ppm, whereas the carbon atoms of C(7)=O were detected at 160.55–160.53 ppm. Alternatively, carbon atoms of the C(24)=O and C(41)=O groups of all new bioconjugates gave signals in the range of 174.11–173.06 ppm and 168.32–168.29 ppm, respectively. The diagnostics signal for C(26) as well as C(30) atoms in the 1,2,3-triazole ring in compounds (**15**–**21**) are observed in the range of 143.40–143.25 ppm and 123.59–123.53 ppm. Additionally, signals originating from the indole ring carbon atoms are observed in the 135.96–115.56 ppm range. The carbon atoms of the HC≡C–CH_2_ group in substrates (**8**–**12**) are observed in the range 74.76–74.70 ppm, 77.80–77.72 ppm, 51.79–51.76 ppm, and 77.80–74.70, respectively.

### 2.2 PM5 Calculations

**Figure 4:**
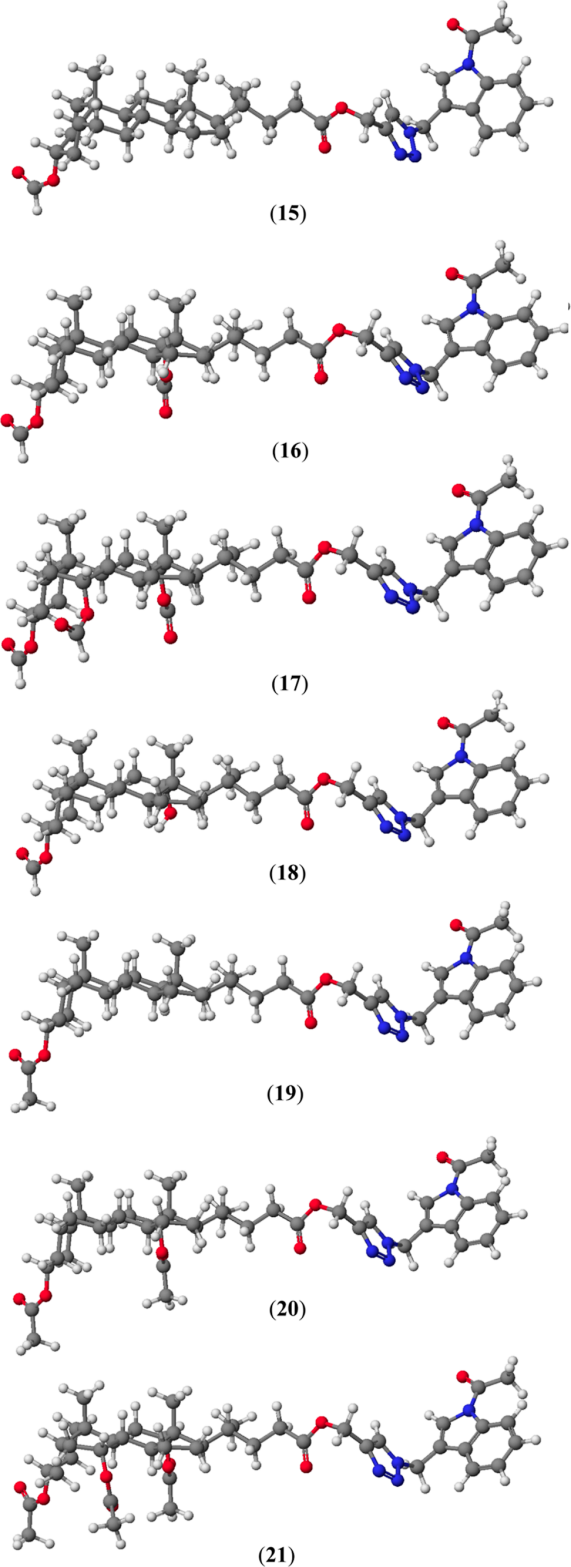
Molecular models of bioconjugates (**15**–**21**) calculated by the PM5 method.

The molecular models of all bioconjugates are shown in Figure 4. The final heats of formation (HOF) for the bioconjugates (**15**–**21**) are presented in Table 1.

**Table 1:** Heat of Formation (HOF) [kcal/mol] Bioconjugates (**15–21**)

compound	HOF [kcal/mol]
**15**	–187.4677
**16**	–277.1396
**17**	–362.3263
**18**	–229.9797
**19**	–196.5772
**20**	–282.5585
**21**	–376.1200

The lowest values of HOF for bioconjugates of bile acids and gramine (**15**–**21**) are observed for cholic acids and their conjugates (**17**) and (**21**). The number of hydroxyl groups in the bile acid skeleton lowers the value of the determinant of the HOF. The same relationship was observed for blocked hydroxyl groups by formyl and acetate groups. The OAc and OAc groups facilitate the formation of intramolecular H-bonds and stable host–guest complexes. These complexes may be stabilized by H-bonding or electrostatic interactions arising from the bile acid molecule’s OCHO and OAc groups. The HOF value decreases with increasing numbers of OCHO and OAc groups in the bile acids skeleton.

Bioconjugates with hydroxyl groups blocked with formyl groups (**15**–**17**) also have higher HOF values than derivatives with acetic groups. This fact can be explained by their smaller steric hindrance and lower electron density, which are not conducive to creating interactions with other molecules. In contrast, comparing the HOF values of bioconjugates of deoxycholic acid (**16**) and (**18**), in which one or two hydroxyl groups have been blocked, the bioconjugate with the unblocked hydroxyl group at C(12) has a higher HOF value than the one with all blocked groups.

### 2.3 Hemolytic Activity

The effect of structural modifications of bile acid molecules on their ability to incorporate into cell membranes was investigated using red blood cells (RBCs) as a model.^34,35^ Our previous study has shown that at a concentration of 0.1 mg/mL, the formyl modification of bile acids increases their hemolytic activity, especially in the case of noncytotoxic cholic acid. The hemolytic activity of acetyl-LCA and DCA was significantly higher than their native forms, whereas the acetyl-CA was not active as membrane perturbing agents at the same concentration.^36^

**Figure 5:**
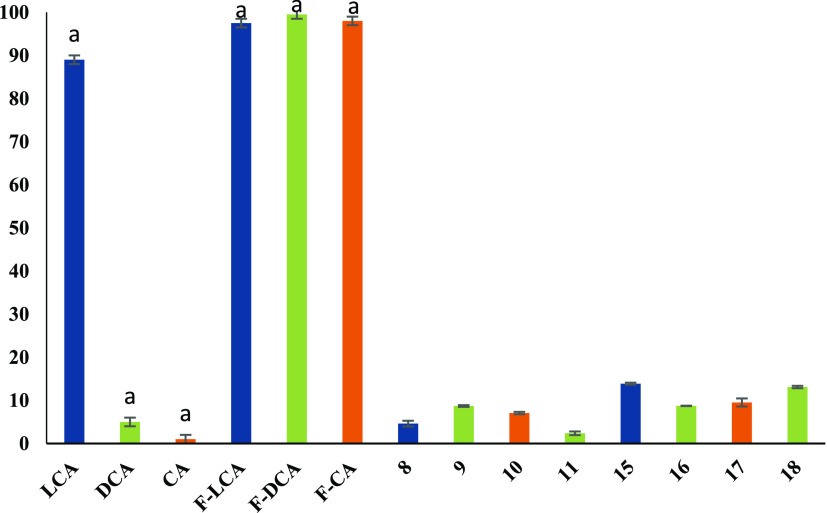
Hemolytic activity of bile acids (LCA, DCA, and CA), their formyloxy derivatives (F-LCA, F-DCA, and F-CA), new esters (**8**–**11**), and bioconjugates with gramine (**15**–**18**). The bile acid derivatives are marked with a different pattern. The compounds were tested at a concentration of 0.1 mg/mL. ^a^Data are published in ref (36).

**Figure 6:**
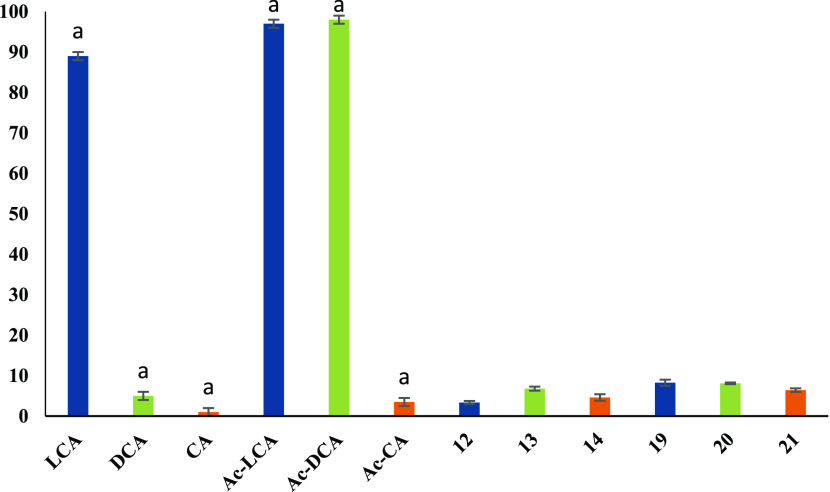
Hemolytic activity of bile acids (LCA, DCA, and CA), their acetyl derivatives (Ac-LCA, Ac-DCA, and Ac-CA), new esters (**12**–**14**), and bioconjugates with gramine (**19**–**21**). The bile acid derivatives are marked with different pattern. The compounds were tested at a concentration of 0.1 mg/mL. ^a^Data are published in ref (36).

As shown in Figure 5, the hemolytic activity (%) of all synthesized formyl derivatives of bile acids propargyl esters (**8**–**11**) and their bioconjugates with modified gramine molecules linked with 1,2,3-triazole ring (**15**–**18**), is significantly decreased (in the range of 2.36–13.87%) in comparison with formyl derivatives (F-LCA, F-DCA, and F-CA in the range of 94–99%). Similarly, acetyl derivatives of bile acid propargyl esters (**12**–**13**) and their triazole bioconjugates (**19**–**20**) are less cytotoxic (3.36–8.26%) than acetylated bile acids (Ac-LCA, Ac-DCA—97–98%). A notable exception is cholic acid derivatives, where the hemolytic activity has not changed significantly (Figure 6).

**Figure 7:**
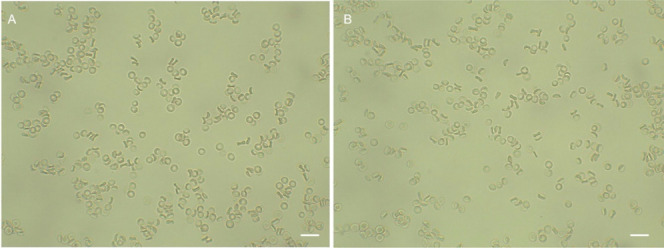
Effect of derivatives **10** (A) and **17** (B) (0.1 mg/mL) on RBC shape as observed in light microscopy (60 min, 37 °C) at 63× magnification. Scale bar—10 μm.

As mentioned before, as amphiphilic compounds, bile acids interact easily with the lipid bilayer of RBC and modify RBC shape from dysocytic to echinocytic or stomatocytic, depending on their chemical structure and concentration used.^36^ As shown in Figure 7, after incubation with derivatives **10** and **17**, the RBC shape is dyscocytic. Therefore, structural modification of bile acid formyl derivatives by introducing indole and 1,2,3-triazole moieties modifies their interaction with the RBC membrane and consequently does not affect the discocytic RBC shape. A similar result is observed in the acetyl derivatives.

### 2.4 Molecular Docking

The PDB IDs for the studied macromolecular structures were 2Q85 and 5V5Z. The possible interactions between the analyzed structures and the protein domains are presented below. The graphical representations reflect the “best” ligand pose in each of the protein domains. It is the pose with the lowest binding energy.

**Figure 8:**
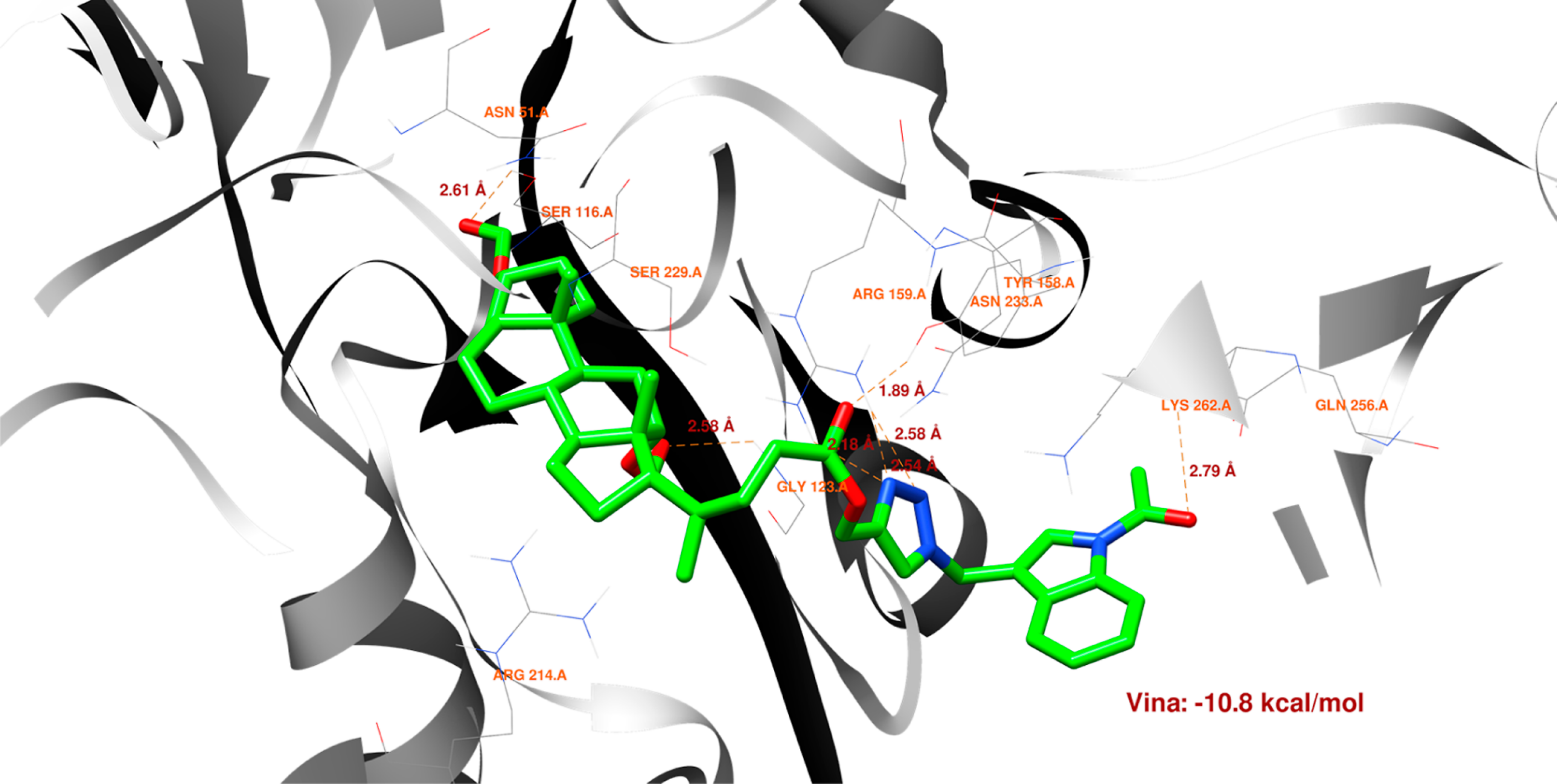
Structure of bioconjugate **16** (Figure 4) inside the active site of the 2Q85 protein domain with yellow marked possible H-bonds.

**Figure 9:**
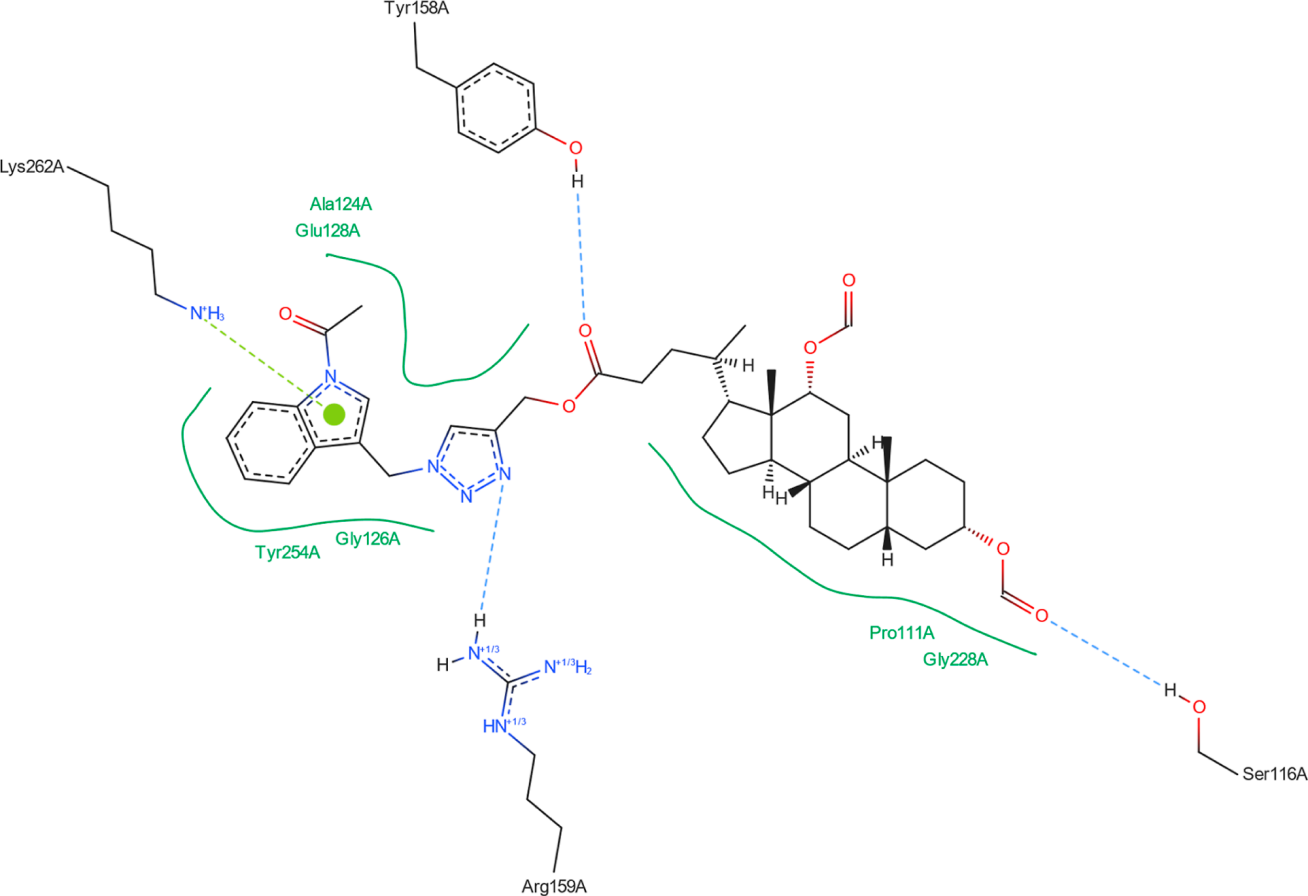
Structure of bioconjugate **16** (Figure 4) inside the active site of the 2Q85 protein domain with marked following interactions: green solid lines—hydrophobic contact, light-blue—hydrogen bond, and light-green—cation–pi interaction.

**Figure 10:**
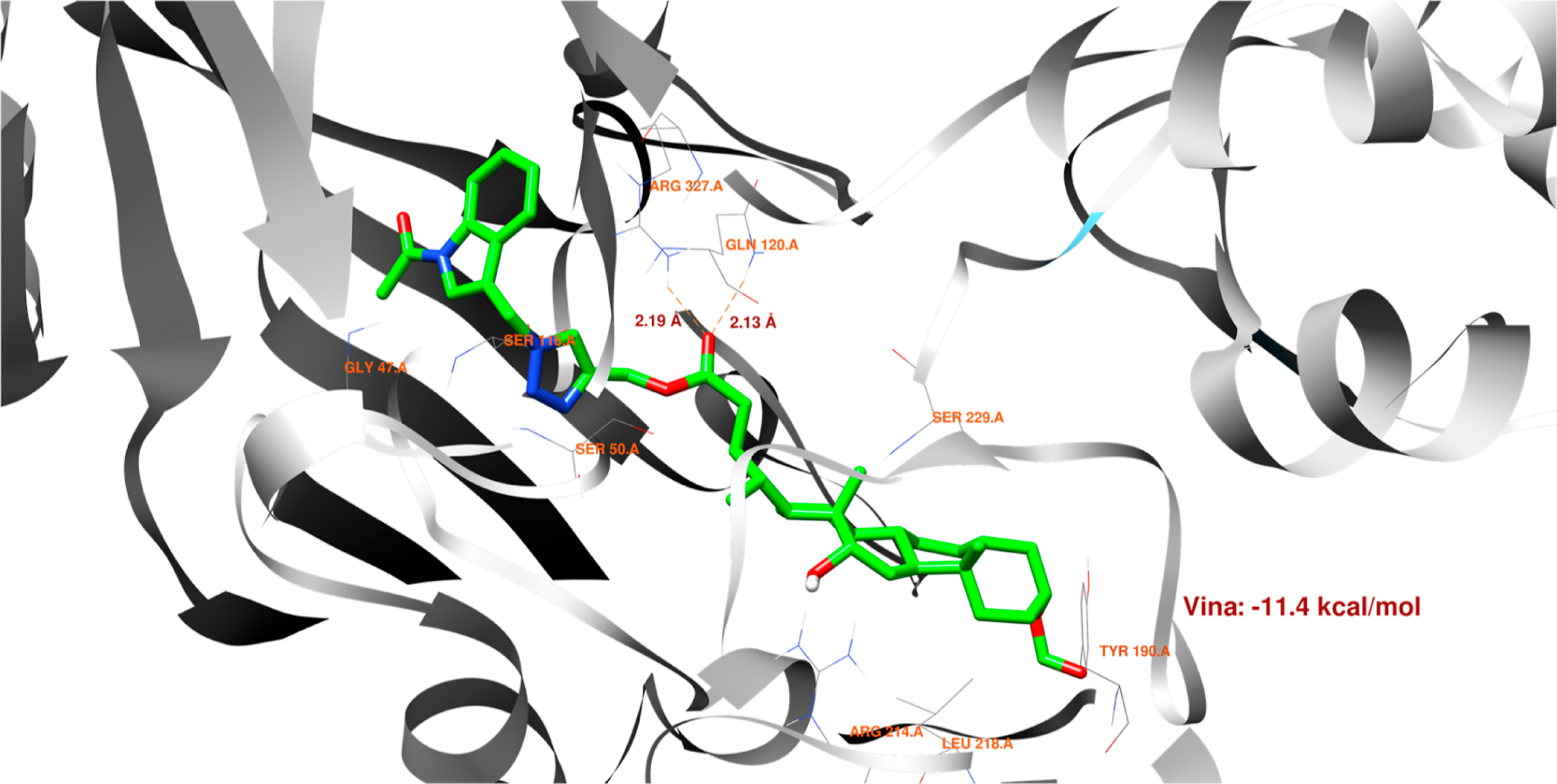
Structure of bioconjugate **18** (Figure 4) inside the active site of the 2Q85 protein domain with yellow marked possible H-bonds.

**Figure 11:**
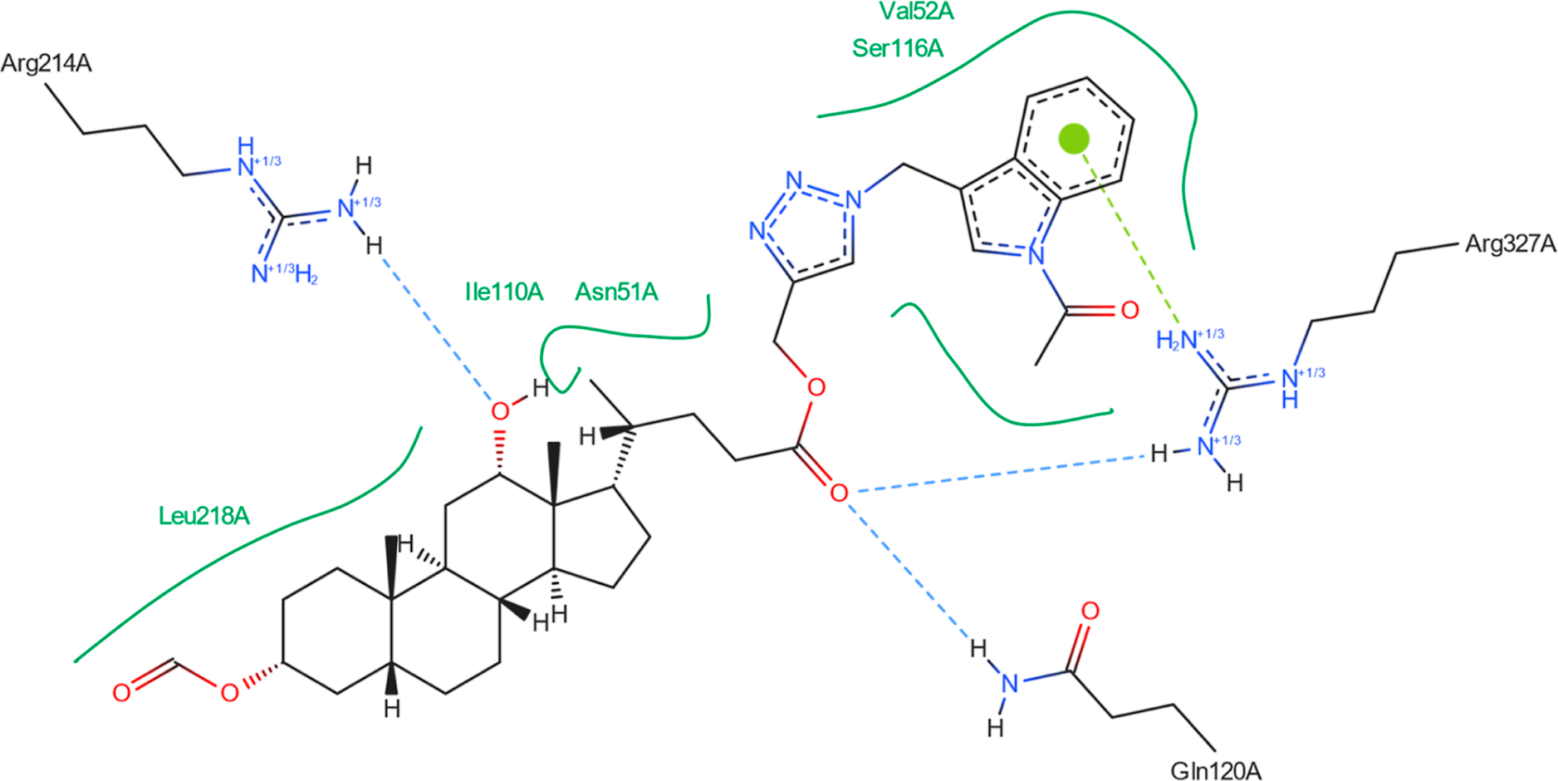
Structure of bioconjugate **18** (Figure 4) inside the active site of the 2Q85 protein domain with marked following interactions: green solid lines—hydrophobic contact, light-blue—hydrogen bond, and light-green—cation–pi interaction.

**Figure 12:**
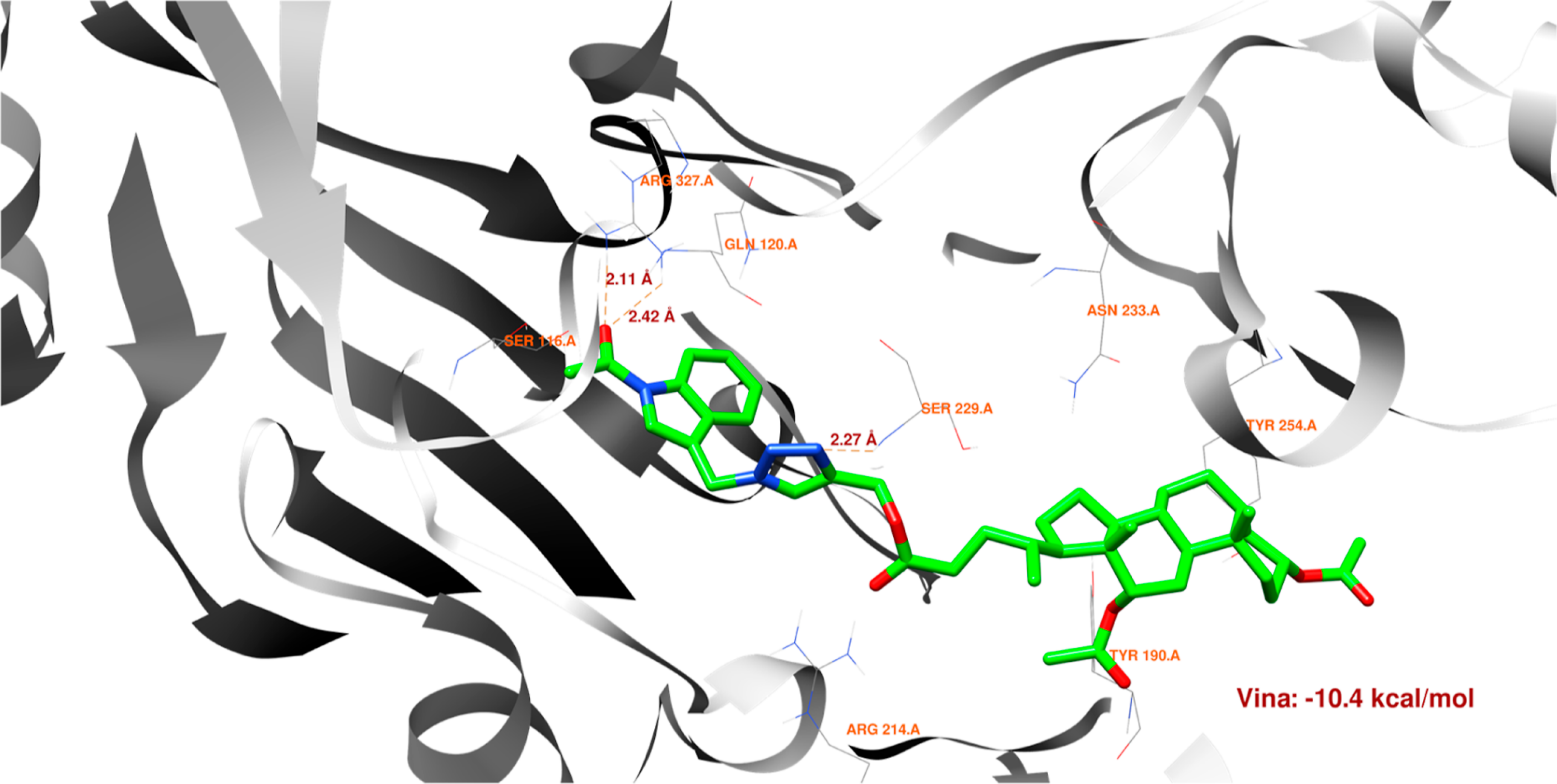
Structure of bioconjugate **20** (Figure 4) inside the active site of the 2Q85 protein domain with yellow marked possible H-bonds.

**Figure 13:**
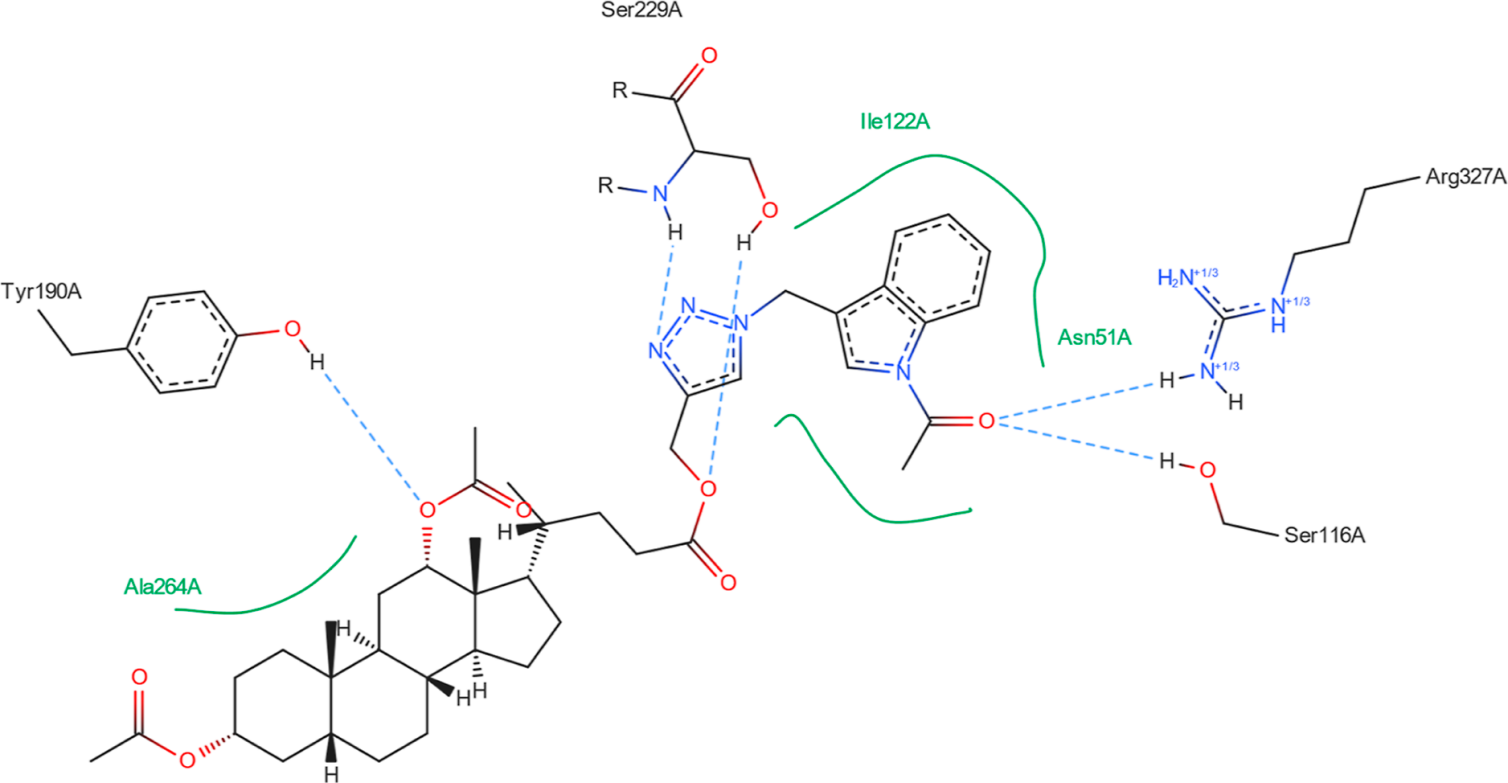
Structure of bioconjugate **20** (Figure 4) inside the active site of the 2Q85 protein domain with marked following interactions: green solid lines—hydrophobic contact and light-blue—hydrogen bond.

Figures 8–13 show how the best poses of the **16**th, **18**th, and **20**th structures interact with the 2Q85 protein domain. For compound **16**, six hydrogen bonds (Figure 8) could be formed between the ligand and the protein domain. The weakest hydrogen bond could occur between the ligand’s oxygen and the protein’s LYS-262 amino acid due to the longest distance. The strongest hydrogen bond could occur between the ligand’s oxygen atom and the TYR-158 residue. Figure 9 indicates the possibility of a cation–pi interaction. For **18**, one hydrogen bond could occur between the ligand and the GLN-120 or ARG-327 residues. Both are strong and comparable (Figure 10). Figure 11 shows the possibility of two hydrogen bond formations along with cation–pi interaction. For derivative **20**, two hydrogen bonds could form between the ligand and the protein domain. Both with comparable strength. Because of the shorter length (2.11 Å), a hydrogen bond between the ligand’s oxygen and the ARG-327 residue is more likely to form. Figure 13 suggests that two additional hydrogen bonds can be formed between the ligand and SER-229 and TYR-190.

**Figure 14:**
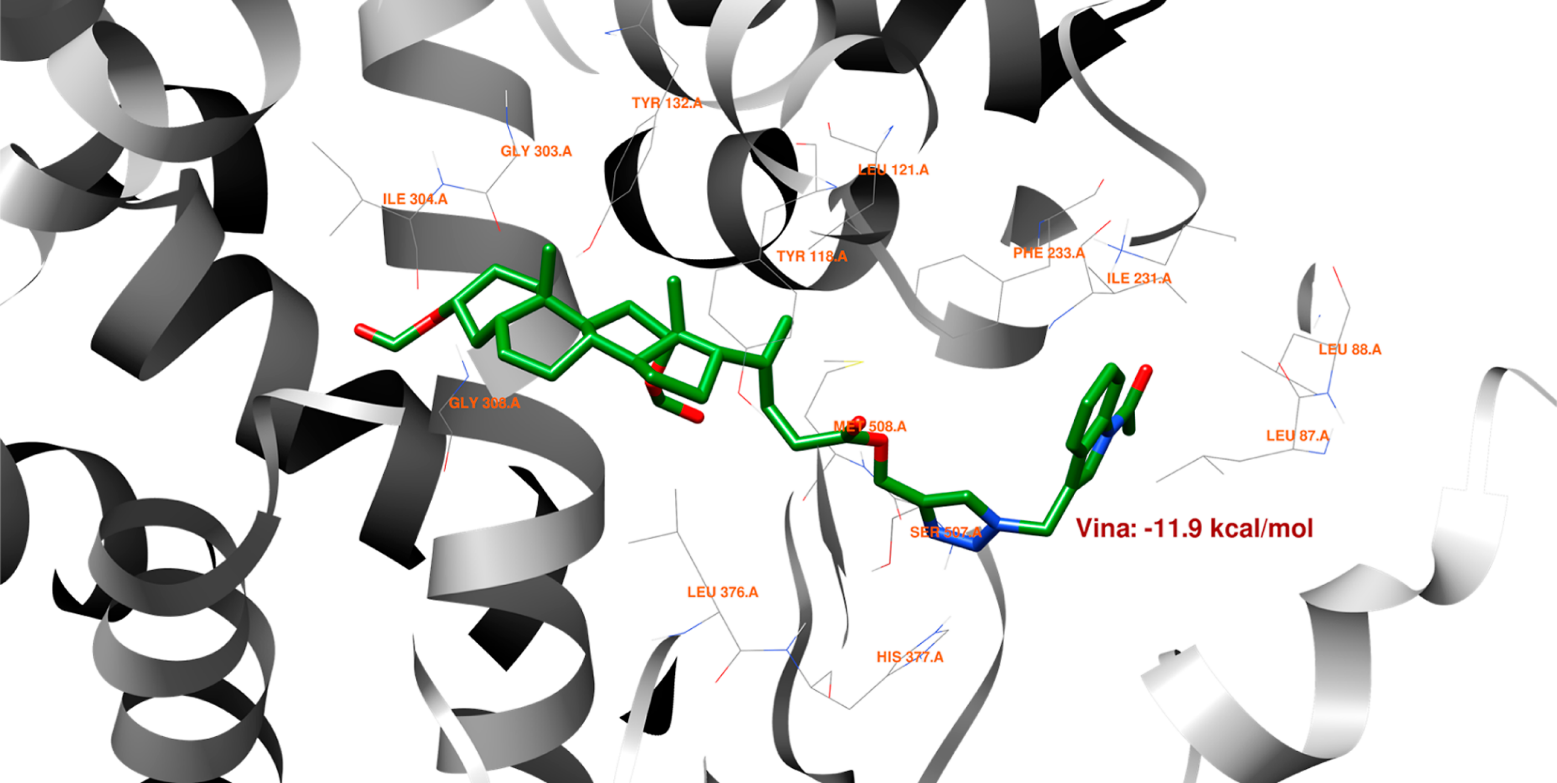
Structure of bioconjugate **16** (Figure 4) inside the active site of the 5V5Z protein domain.

**Figure 15:**
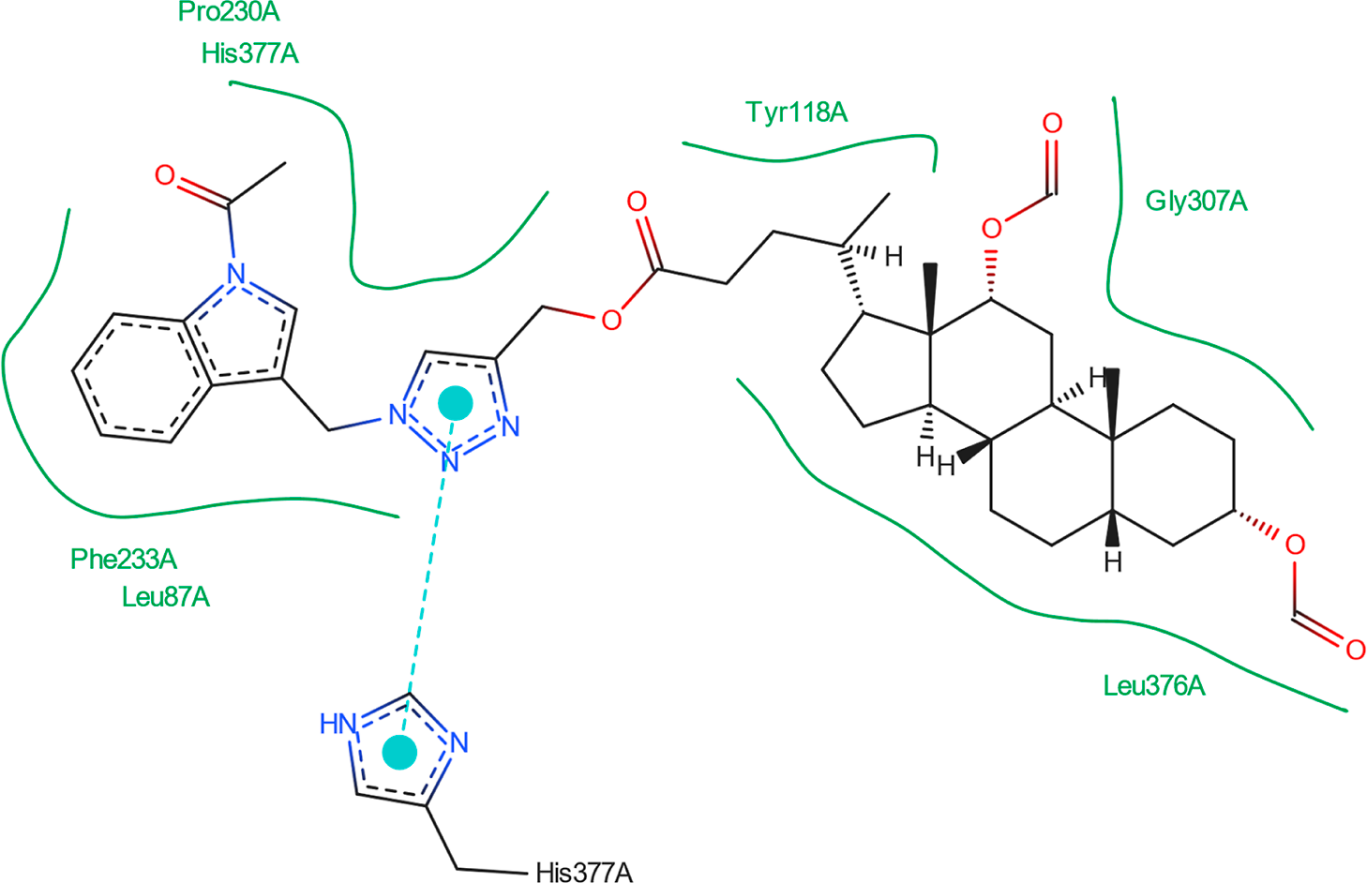
Structure of bioconjugate **16** (Figure 4) inside the active site of the 5V5Z protein domain with marked following interactions: green solid lines—hydrophobic contact and cyan—pi–pi interaction.

**Figure 16:**
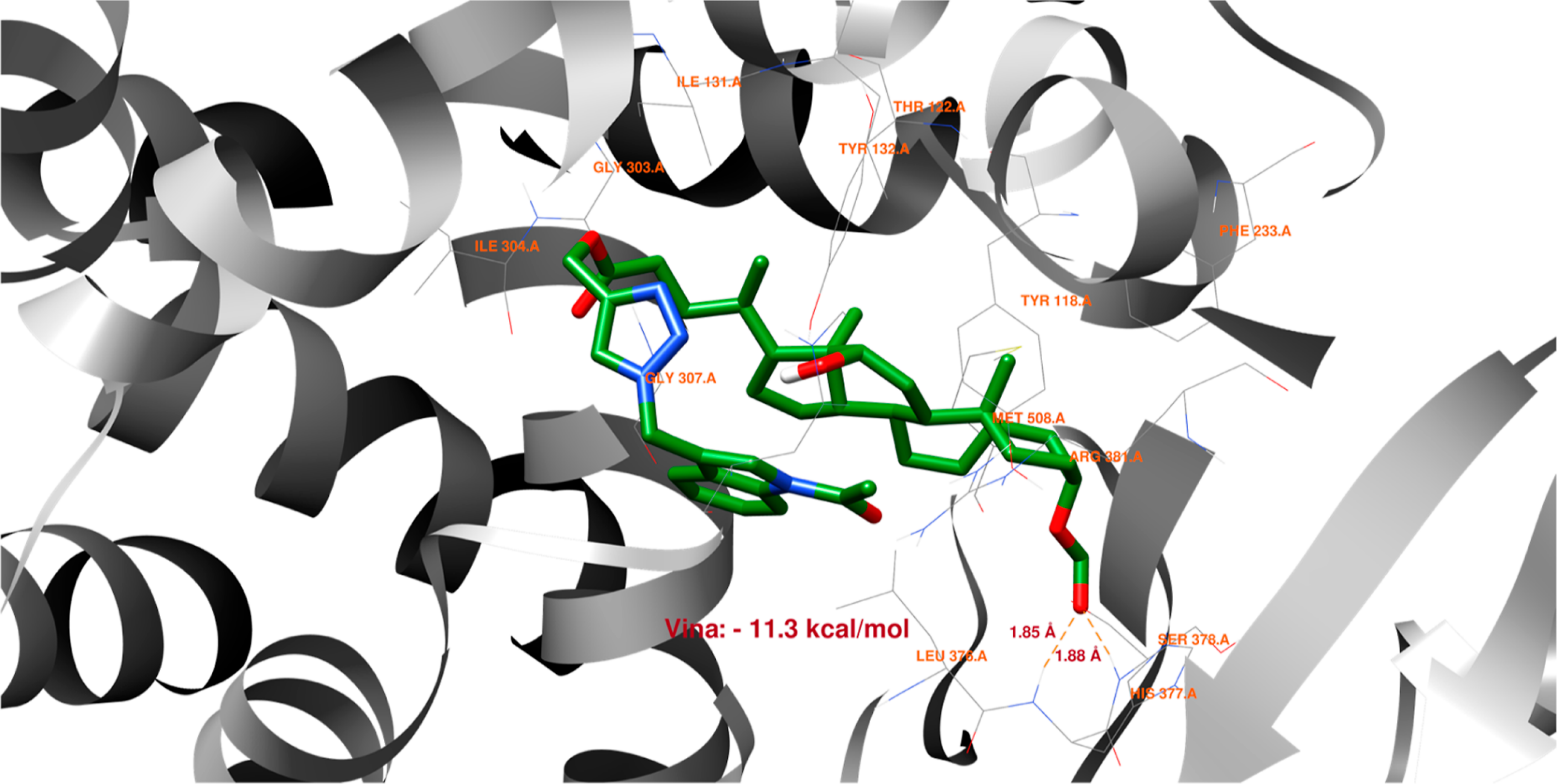
Structure of bioconjugate **18** (Figure 4) inside the active site of the 5V5Z protein domain with yellow marked possible H-bonds.

**Figure 17:**
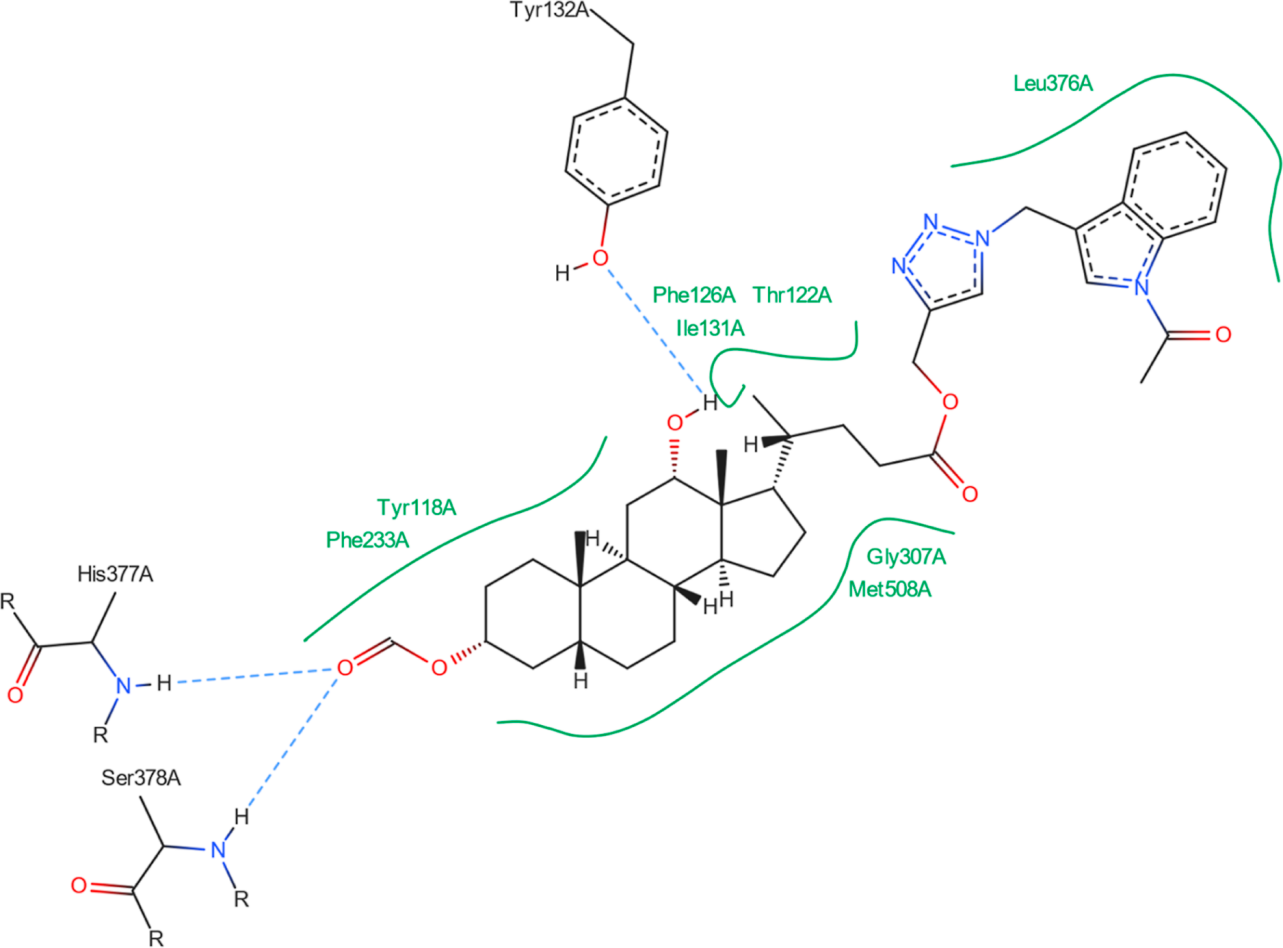
Structure of bioconjugate **18** (Figure 4) inside the active site of the 5V5Z protein domain with marked following interactions: green solid lines—hydrophobic contact and light-blue—hydrogen bond.

**Figure 18:**
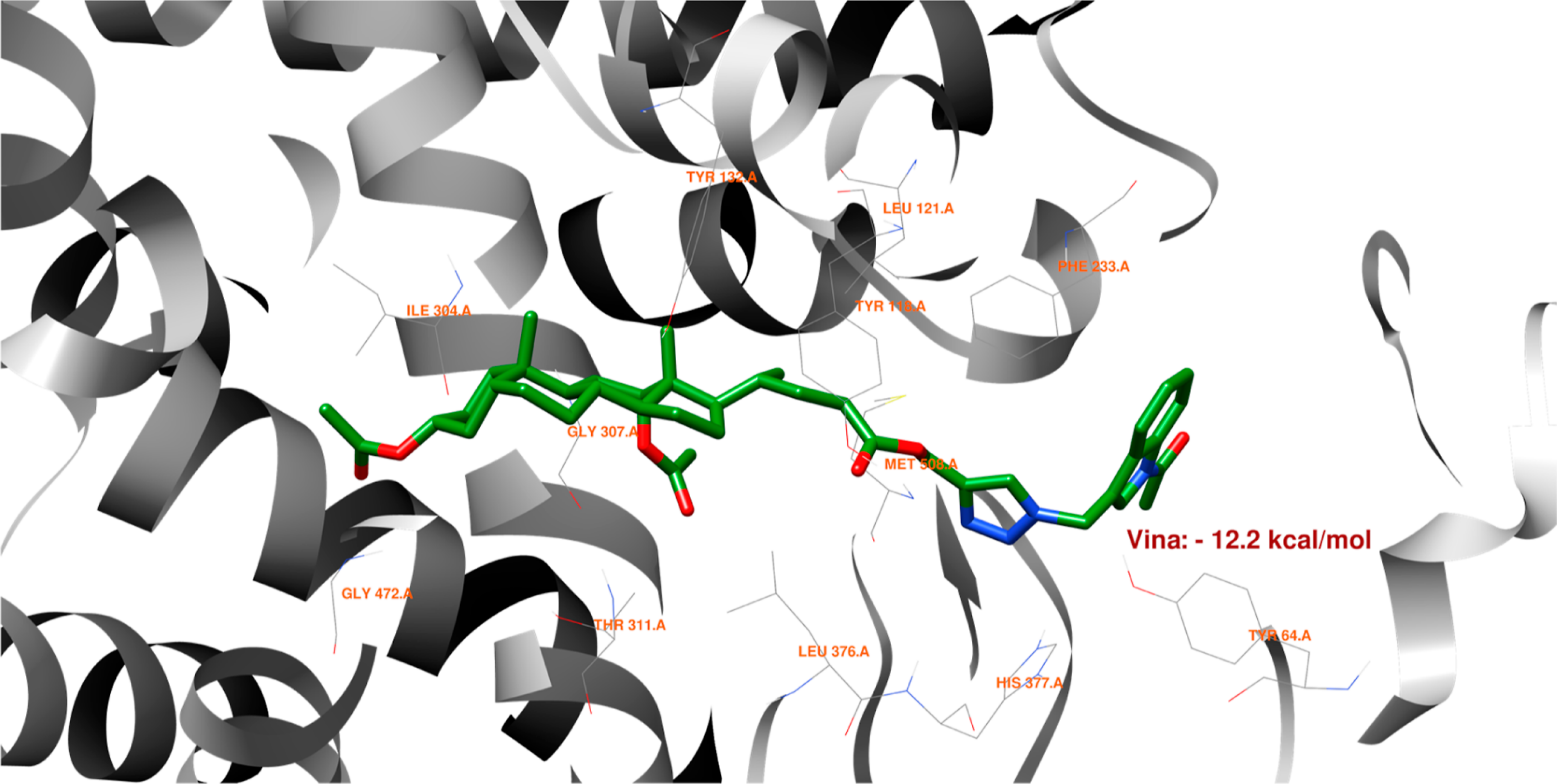
Structure of bioconjugate **20** (Figure 4) inside the active site of the 5V5Z protein domain.

**Figure 19:**
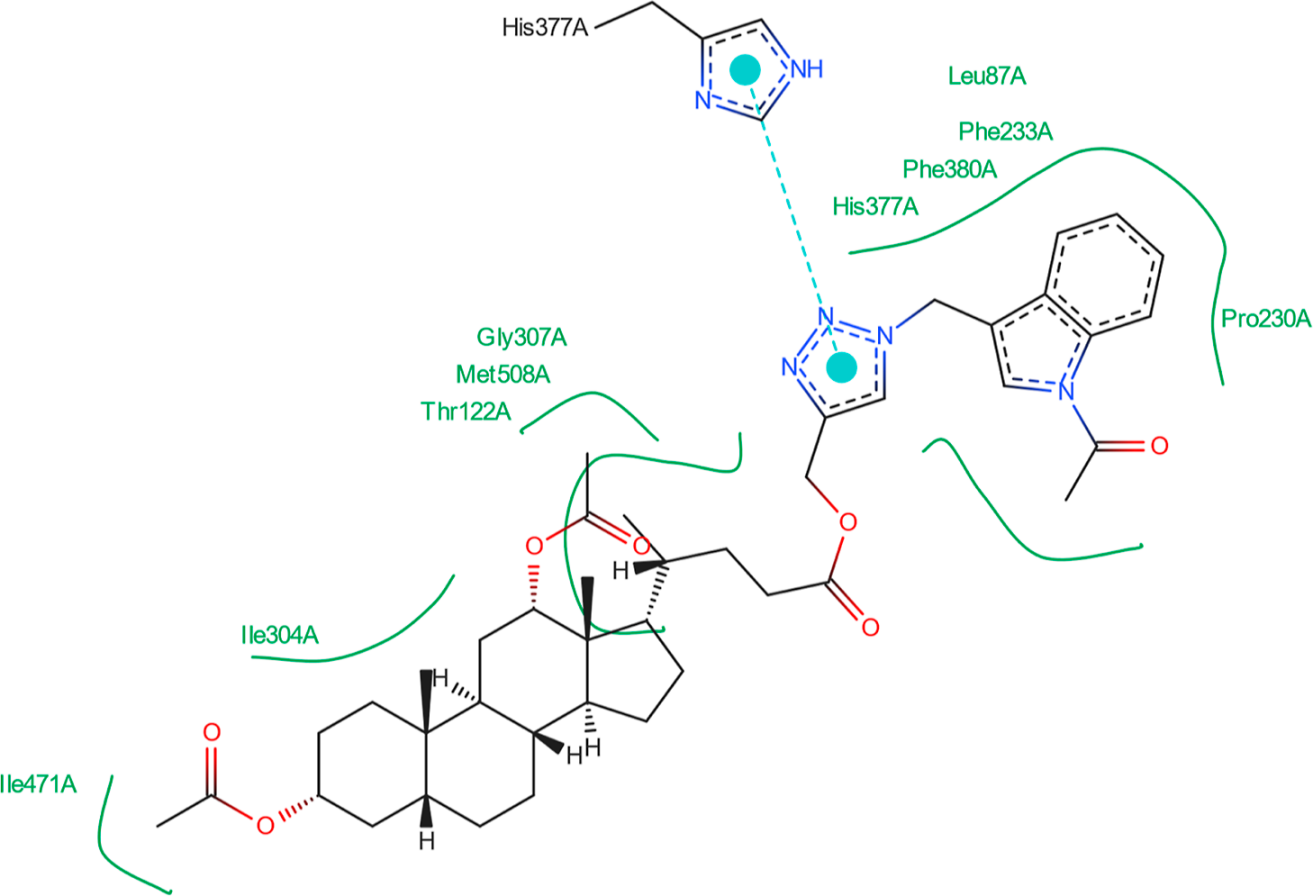
Structure of bioconjugate **20** (Figure 4) inside the active site of the 5V5Z protein domain with marked following interactions: green solid lines—hydrophobic contact, cyan—pi–pi interaction.

Figure 14 shows that no hydrogen bonds can form between the best pose of the **16th**th structure and the 5V5Z protein domain. Figure 15 indicates that the **16**th structure can be additionally stabilized by the pi–pi interaction. Figure 16 indicates that one hydrogen bond could form between the **18**th structure’s best pose and SER-378 or HIS-377 residues in the 5V5Z protein domain. Figure 17 shows one additional hydrogen bond between the ligand and the TYR-132 amino acid. Figure 18 displays no hydrogen bonds between the **20**th structure’s best pose and that of the 5V5Z protein domain. Figure 19 shows the additional stabilization due to pi–pi interactions.

The docked molecules possess a non-negligible affinity to the 2Q85 protein domain, implying that they could be suitable inhibitors. Each figure (Figures 8, 10, 12, 14, 16, and 18) shows binding energies with a specified kcal/mol unit. Despite forming a lower number of possible hydrogen bonds, the affinities of structures **16**, **18**, and **20** (Figure 4) for the 5V5Z protein domain are even a little stronger than those of the 2Q85 domain. Figures 9, 11, 13, 15, 17, and 19 explain the interaction between the ligands and the protein domains.

## 3 Conclusions

In summary, this study presents the synthesis and chemical characterization of new formyl and acetyl derivatives of bile acid propargyl esters and their bioconjugates with modified gramine molecules linked with a 1,2,3-triazole ring. The results obtained in this study confirm that structural modification of formyl and acetyl derivatives of bile acids results in alterations of their cell membrane interactions and decreases their hemolytic activity. The molecular docking investigations reveal that the selected compounds have an affinity for the protein targets of interest (2Q85 and 5V5Z), which are associated with antibacterial and antifungal activity, respectively. Therefore, they are likely to be biologically active compounds.

## 4 Experimental Section

### 4.1 Chemistry

#### 4.1.1 Synthesis of *N*-Acetyl-3-azidemethylindole (**4**)

*N*-Acetyl-3-hydroxymethylindole (**3**) (0.54 mmol, 102 mg) was dissolved in dimethylformamide (2 mL). Then diphenylphosphoryl azide (DPPA) (1 mmol, 275 mg) diluted in dimethylformamide (1 mL) was added. The reaction mixture was cooled to 0 °C, and then 1,8-diazabicyclo[5.4.0]undec-7-ene (DBU) (1 mmol, 152 mg) was added dropwise. The mixture was stirred at 0 °C for 2 h. The reaction was monitored by TLC (PhMe/EtOAc 5:1). The distilled water was added (5 mL), and the reaction mixture was extracted with ethyl acetate, washed with distilled water and brine, then dried over anhydrous Na_2_SO_4_. The organic layer was evaporated under reduced pressure. The crude product was purified by column chromatography (PhMe/EtOAc 50:1).

Yellow oil, yield: 68 mg, 59%, ^1^H NMR (400 MHz, CDCl_3_): δ 8.44 (d, *J* = 8.2 Hz, 1H, 40 H), 7.59 (d, *J* = 8.2 Hz, 1H, 37 H), 7.44 (s, 1H, 33 H), 7.40–7.30 (m, 2H, 38 H, 39 H), 4.49 (d, *J* = 1.0 Hz, 2H, 31-CH_2_–N_3_), 2.64 (s, 3H, 42-CH_3_). ^13^C{^1^H}NMR (101 MHz, CDCl_3_): δ 168.4 (C-41), 136.0, 129.8, 128.8, 125.8, 123.9, 120.1, 118.9, 116.8, 46.1 (31-CH_2_–N_3_), 23.9 (42-CH_3_). FT-IR (KBr, cm^–1^) ν_max_: 3120 (=C–H), 3049 (=C–H), 2928 (C–H), 2871 (C–H), 2107 (–N_3_), 1706 (C=O). EI-MS (*m*/*z*): 214 (25%). Anal. Calcd for C_11_H_10_N_4_O (MW = 214.09): C, 61.67; H, 4.71; N, 26.15; O, 7.47. Found: C, 61.69; H, 4.68; N, 26.17; O, 7.46.

#### 4.1.2 General Procedure for the Preparation of Compounds (**8**–**11**)

Propargyl lithocholate (or deoxycholate and cholate) (7.23 mmol, 1.0 equiv) was dissolved in 20 mL of HCOOH (90%) and heated at 60 °C in a water bath for 4 h. The reaction was monitored by TLC. The reaction mixture was cooled to room temperature and poured onto ice, and the white solid was washed with water. The crude product was obtained and purified by column chromatography (eluent CHCl_3_). The reaction yield was: (**8**) 3.0 g, 94%, (**9**) 3.3 g, 94%, (**10**) 3.72 g, 97%, and (**11**) 200 mg, 6% (product of nonexhaustive formylation), respectively.

##### 4.1.2.1 Propargyl 3α-Formyl-5β-cholan-24-oate (**8**)

White oil, ^1^H NMR (300 MHz, CDCl_3_): δ 8.04 (d, *J* = 1.0 Hz, 1H, 3α-OCHO), 4.94–4.78 (m, 1H, 3β-H), 4.68 (d, *J* = 3.0 Hz, 2H, O–CH_2_), 2.47 (t, *J* = 2.5 Hz, 1H, –C≡CH), 0.94 (s, 3H, CH_3_-19), 0.92 (d, *J* = 6.4 Hz, 3H, CH_3_-21), 0.64 (s, 3H, CH_3_-18). ^13^C{^1^H}NMR (76 MHz, CDCl_3_): δ 173.4 (C-24), 160.8 (3-OCHO), 77.8 (–C≡), 74.7 (≡CH), 74.4 (C-3), 56.4, 55.9, 51.8 (OCH_2_), 42.7, 41.9, 40.4, 40.1, 35.8, 35.3, 34.9, 34.6, 32.2, 30.9, 30.8, 28.2, 27.0, 26.6, 26.3, 24.2, 23.3, 20.8 (C-19), 18.2 (C-21), 12.0 (C-18). FT-IR (KBr, cm^–1^) ν_max_: 3302 (C≡C–H), 2938 (C–H), 2867 (C–H), 1746 (C=O), 1714 (C=O), 1436 (C–H), 1376 (C–O), 1202 (C–O). ESI-MS *m*/*z*: 465 [M + Na]^+^ (100%), 481 [M + K]^+^ (10%). Anal. Calcd for C_28_H_42_O_4_ (MW = 442.31): C, 75.98; H, 9.56; O, 14.46. Found: C, 75.95; H, 9.58; O, 14.47.

##### 4.1.2.2 Propargyl 3α,12α-Diformyl-5β-cholan-24-oate (**9**)

White oil, ^1^H NMR (300 MHz, CDCl_3_): δ 8.14 (s, 1H, 12α-OCHO), 8.04 (d, *J* = 0.9 Hz, 1H, 3α-OCHO), 5.25 (s, 1H, 12β-H), 4.89–4.79 (m, 1H, 3β-H), 4.68 (d, *J* = 3.0 Hz, 2H, O–CH_2_), 2.48 (t, *J* = 2.5 Hz, 1H, –C≡CH), 0.93 (s, 3H, CH_3_-19), 0.83 (d, *J* = 6.3 Hz, 3H, CH_3_-21), 0.75 (s, 3H, CH_3_-18). ^13^C{^1^H} NMR (76 MHz, CDCl_3_): δ 173.2 (C-24), 160.7 (12-OCHO), 160.6 (3-OCHO), 77.7 (C≡), 76.0 (C-12), 74.8 (≡CH), 74.1 (C-3), 51.8, 49.2, 47.3, 45.0, 41.7, 35.6, 34.8, 34.7, 34.2, 34.0, 32.1, 30.9, 30.5, 27.4, 26.8, 26.5, 25.9, 25.7, 23.4, 22.9 (C-19), 17.5 (C-21), 12.3 (C-18). FT-IR (KBr, cm^–1^) ν_max_: 3249 (C≡C–H), 2935 (C–H), 2867 (C–H), 1717 (C=O), 1450 (C–H), 1378 (C–O), 1251 (C–O), 1167 (C–H). ESI-MS *m*/*z*: 525 [M + K]^+^ (100%), 509 [M + Na]^+^ (50%). Anal. Calcd for C_29_H_42_O_6_ (MW = 486.30): C, 71.57; H, 8.70; O, 19.73. Found: C, 71.61; H, 8.68; O, 19.71.

##### 4.1.2.3 Propargyl 3α,7α,12α-Triformyl-5β-cholan-24-oate (**10**)

White oil, ^1^H NMR (300 MHz, CDCl_3_): δ 8.16 (s, 1H, 12α-OCHO), 8.11 (s, 1H, 7α-OCHO), 8.03 (d, *J* = 1.0 Hz, 1H, 3α-OCHO), 5.27 (s, 1H, 12β-H), 5.07 (s, 1H, 7β-H), 4.76–4.69 (m, 1H, 3β-H), 4.67 (dd, *J* = 2.5 Hz, 2H, OCH_2_) 2.47 (t, *J* = 2.5 Hz, 1H, –C≡CH), 0.95 (s, 3H, CH_3_-19), 0.84 (d, *J* = 6.4 Hz, 3H, CH_3_-21), 0.76 (s, 3H, CH_3_-18). ^13^C{^1^H} NMR (76 MHz, CDCl_3_): δ173.1 (C-24), 160.5 (12, 7-OCHO), 160.5 (3-OCHO), 77.7 (–C≡), 75.2 (C-12), 74.7 (≡CH), 73.7 (C-3), 70.6 (C-7), 51.8 (O–CH_2_), 47.2, 45.0, 43.0, 40.8, 37.7, 34.7, 34.5, 34.4, 34.3, 31.3, 30.8, 30.5, 28.5, 27.1, 26.6, 25.5, 22.8, 22.3 (C-19), 17.4 (C-21), 12.1 (C-18). FT-IR (KBr, cm^–1^) ν_max_: 3285 (C≡C–H), 2942 (C–H), 2872 (C–H), 1716 (C=O), 1468 (C–H), 1449 (C–H), 1381 (C–O), 1181 (C–O). ESI-MS *m*/*z*: 553[M + Na]^+^ (100%), 569 [M + K]^+^ (10%). Anal. Calcd for C_30_H_42_O_8_ (MW = 530.29): C, 67.90; H, 7.98; O, 24.12. Found: C, 67.91; H, 8.01; O, 24.08.

##### 4.1.2.4 Propargyl 3α-Formyl-12α-hydroxy-5β-cholan-24-oate (**11**)

White oil, ^1^H NMR (300 MHz, CDCl_3_): δ 8.03 (d, *J* = 1.0 Hz, 1H, 3α-OCHO), 4.91–4.79 (m, 1H, 3β-H), 4.68 (dd, *J*_1_ = 2.5, *J*_2_ = 0.5 Hz, 2H, O–CH_2_), 3.99 (s, 1H, 12β-H), 2.47 (t, *J* = 2.5 Hz, 1H, –C≡CH), 0.98 (d, *J* = 6.3 Hz, 3H, CH_3_-21), 0.93 (s, 3H, CH_3_-19), 0.68 (s, 3H, CH_3_-18). ^13^C{^1^H} NMR (76 MHz, CDCl_3_): δ 173.3 (C-24), 160.8 (3-OCHO), 77.8 (–C≡), 74.7 (≡CH), 74.2 (C-3), 73.1 (C-12), 51.8, 51.7, 48.2, 47.3, 46.5, 41.8, 35.9, 35.0, 34.8, 34.1, 34.0, 33.6, 32.1, 31.0, 30.7, 28.7, 27.4, 26.9, 26.5, 26.0, 23.6, 23.1 (C-19), 17.3 (C-21), 12.7 (C-18). FT-IR (KBr, cm^–1^) ν_max_: 3510 (O–H), 3253 (C≡C–H), 2944 (C–H), 2866 (C–H), 2129 (C≡C), 1725 (C=O), 1466 (C–H), 1380 (C–O), 1194 (C–O). ESI-MS *m*/*z*: 481 [M + Na]^+^ (100%), 497 [M + K]^+^ (20%). Anal. Calcd for C_28_H_42_O_5_ (MW = 458.30): C, 73.33; H, 9.23; O, 17.44. Found: C, 73.40; H, 9.19; O, 17.41.

##### 4.1.2.5 Synthesis of Propargyl 3α-Acetyl-5β-cholan-24-oate (**12**)

Propargyl lithocholate (5 mmol, 2 g) was dissolved in 2 mL of anhydrous pyridine, and 2 mL of acetic anhydride was placed in the round-bottom flask. The mixture was stirred at room temperature for 22 h. The reaction was monitored by TLC. The reaction mixture was poured onto ice, extracted with CHCl_3_, washed with 5% NaHCO_3_ solution, water, and brine, and dried over anhydrous Na_2_SO_4_. 2.14 g of crude product (97%) was obtained as an oil and purified by column chromatography (eluent CHCl_3_: hexane fraction 10:1). 1.52 g portion of pure product (**12**) (71%) was obtained.

White oil, ^1^H NMR (300 MHz, CDCl_3_): δ 4.77–4.67 (m, 3H, 3β-H), 4.68 (dd, *J*_1_ = 2.5, *J*_2_ = 0.5 Hz, 2H, O–CH_2_) 2.46 (t, *J* = 6.8 Hz, 1H, –C≡CH), 2.03 (s, 3H, 3-OAc), 0.92 (s, 3H, CH_3_-19), 0.91 (d, *J* = 6.1 Hz, 3H, CH_3_-21), 0.64 (s, 3H, CH_3_-18). ^13^C{^1^H} NMR (76 MHz, CDCl_3_): δ 173.4 (C-24), 170.7 (3-AcO), 77.8 (C≡), 74.7 (≡CH), 74.4 (C-3), 56.5, 55.9, 53.2, 51.8, 42.7, 41.9, 40.4, 40.1, 35.8, 35.3, 35.0, 34.6, 32.2, 31.0, 30.8, 28.2, 27.0, 26.6, 26.3, 24.2, 23.3, 21.5, 20.8 (C-19), 18.2 (C-21), 12.0 (C-18). FT-IR (KBr, cm^–1^) ν_max_: 3253 (C≡C–H), 2925 (C–H), 2863 (C–H), 1719 (C=O), 1452 (C–H), 1376 (C–H), 1249 (C–O), 1162 (C–O), 1022 (C–O). ESI-MS *m*/*z*: 479 [M + Na]^+^ (100%), 495 [M + K]^+^ (10%). Anal. Calcd for C_29_H_44_O_4_ (MW = 456.32): C, 76.27; H, 9.71; O, 14.01. Found: C, 76.29; H, 9.67; O, 14.04.

Compounds **13** and **14** were synthesized according to literature data.^39,40^

#### 4.1.3 General Procedure for the Preparation of Compounds (**15**–**21**)

An appropriate bile acid derivative (**8**–**14**) (0,2 mmol, 1.0 equiv) was dissolved in *t*-BuOH/MeOH (5:1), (4 mL) at 60 °C using a water bath. Then an *N*-acethyl-3-azidomethylindole (**4**) (0,2 mmol, 1.0 equiv) was dissolved in *t*-BuOH/MeOH (5:1) (2 mL) was added. CuSO_4_·5H_2_O (0.00002 mmol, 0.0001 equiv) and sodium ascorbate (0.00007 mmol, 0.0004 equiv) in 1 mL of distilled water were added. The mixture was stirred on a magnetic stirrer and heated in a water bath at 60 °C. Subsequent portions of the catalyst were added until the aqueous layer of the reaction mixture turned bluish-green. The reaction was monitored by TLC (PhMe/EtOAc 1:1). An orange solid precipitated as the reaction mixture was stirred. The reaction mixture was extracted with chloroform. The organic layer was evaporated under reduced pressure. The crude product was purified over column chromatography (gradient elution, starting from CHCl_3_/EtOAc 5:1).

##### 4.1.3.1 {1-[(*N*-Acetyl-methylindole)-3-methylene]-1*H*-1,2,3-triazole-4-yl} Methyl 3α-Formyloxy-cholan-24-oate (**15**)

White oil, yield: 108 mg, 82%, ^1^H NMR (400 MHz, CDCl_3_): δ 8.43 (d, *J* = 7.9 Hz, 1H, 40 H), 8.04 (d, *J* = 0.9 Hz, 1H, 3-HCOO), 7.57 (s, 1H, 30 H), 7.54 (br s, 1H, 33-H_indole_), 7.45–7.38 (m, 2H, 38 H, 39 H), 7.31–7.29 (m, 1H, 37 H), 5.68 (d, *J* = 1.0 Hz, 2H, CH_2_-31), 5.16 (d, *J* = 1.4 Hz, 2H, CH_2_-25), 4.89–4.81 (m, 1H, 3β-H), 2.66 (s, 3H, CH_3_-42), 0.93 (s, 3H, CH_3_-19), 0.85 (d, *J* = 6.4 Hz, 3H, CH_3_-21), 0.60 (s, 3H, CH_3_-18). ^13^C{^1^H} NMR (101 MHz, CDCl_3_): δ 174.1 (C-24), 168.3 (C-41), 160.8 (3-OCHO), 143.4 (C-26), 136.0, 128.4, 126.1, 124.6, 124.2, 123.6 (C-30), 118.6, 116.8, 115.6, 74.4 (C-3), 57.4, 56.4, 55.8, 45.5, 42.7, 41.9, 40.4, 40.0, 35.7, 35.2, 34.9, 34.5, 32.2, 31.0, 30.8, 29.7, 28.1, 26.9, 26.6, 26.3, 24.1, 23.3, 20.9 (C-19), 18.9 (C-21), 12.0 (C-18). FT-IR (KBr, cm^–1^) ν_max_: 3434 (C–H), 3140 (=C–H), 2927(C–H), 2865 (C–H), 1717 (C=O). ESI-MS (*m*/*z*): 679 [M + Na]^+^ (100%), 695 [M + K]^+^ (20%), 692 [M + Cl]^−^. Anal. Calcd for C_39_H_52_N_4_O_5_ (MW = 656.39): C, 71.31; H, 7.98; N, 8.53; O, 12.18. Found: C, 71.28; H, 7.87; N, 8.76; O, 12.09.

##### 4.1.3.2 {1-[(*N*-Acetyl-methylindole)-3-methylene]-1*H*-1,2,3-triazole-4-yl} Methyl 3α,12α-Diformyloxy-cholan-24-oate (**16**)

White oil, yield: 97 mg, 69%, ^1^H NMR (400 MHz, CDCl_3_): δ 8.43 (d, *J* = 8.3 Hz, 1H, 40 H), 8.11 (s, 1H, 12-HCOO), 8.03 (d, *J* = 0.9 Hz, 1H, 3-HCOO), 7.57 (s, 1H, 30 H), 7.55 (br s, 1H, 33 H), 7.45–7.38 (m, 2H, 38 H, 39 H), 7.30–7.29 (m, 1H, 37 H), 5.68 (d, *J* = 0.8 Hz, 2H, CH_2_-31), 5.20 (d, *J* = 3.3 Hz, 1H, 12β-H), 5.15 (d, *J* = 2.0 Hz, 2H, CH_2_–25), 4.87–4.80 (m, 1H, 3β-H), 2.66 (s, 3H, CH_3_-42), 0.92 (s, 3H, CH_3_-19), 0.76 (d, *J* = 6.4 Hz, 3H, CH_3_-21), 0.69 (s, 3H, CH_3_-18). ^13^C{^1^H} NMR (101 MHz, CDCl_3_): δ 173.9 (C-24), 168.3 (C-41), 160.6 (3-OCHO), 160.5 (12-OCHO), 143.3 (C-26), 135.9, 128.3, 126.1, 124.8, 124.2, 123.5 (C-30), 118.6, 116.8, 115.6, 75.9 (C-12), 74.1 (C-3), 57.4, 49.2, 47.2, 45.4, 44.9, 41.7, 35.5, 34.7, 34.6, 34.1, 34.0, 32.0, 30.9, 30.5, 27.3, 26.7, 26.4, 25.9, 24.0, 23.4, 22.9 (C-19), 17.4 (C-21), 12.2 (C-18). FT-IR (KBr, cm^–1^) ν_max_: 3143(=C–H), 2941 (C–H), 2872 (C–H), 1716 (C=O). ESI-MS (*m*/*z*): 723 [M + Na]^+^ (100%), 739 [M + K]^+^ (12%), 701 [M + H]^+^ (10%), 799 [M + ClO_4_]^−^, 735 [M + Cl]^−^. Anal. Calcd for C_40_H_52_N_4_O_7_ (MW = 700.38): C, 68.55; H, 7.48; N, 7.99; O, 15.98. Found: C, 68.54; H, 7.46; N, 8.01; O, 15.99.

##### 4.1.3.3 {1-[(*N*-Acetyl-methylindole)-3-methylene]-1*H*-1,2,3-triazole-4-yl} Methyl 3α,7α,12α-Triformyloxy-cholan-24-oate (**17**)

White oil, yield: 103 mg, 69%, ^1^H NMR (400 MHz, CDCl_3_): δ 8.43 (d, *J* = 8.5 Hz, 1H, 40 H), 8.14 (s, 1H, 7-HCOO), 8.10 (s, 1H, 12-HCOO), 8.02 (d, *J* = 0.9 Hz, 1H, 3-HCOO), 7.57 (br s, 1H, 30 H), 7.45–7.38 (m, 2H, 38 H, 39 H), 7.30–7.28 (m, 1H, 37 H), 5.68 (d, *J* = 0.9 Hz, 2H, CH_2_-31), 5.22 (t, *J* = 3.0 Hz, 1H, 12β-H), 5.15 (d, *J* = 2.7 Hz, 2H, CH_2_-25), 5.06 (d, *J* = 3.5 Hz, 1H, 7β-H), 4.75–4.69 (m, 1H, 3β-H), 2.66 (s, 3H, CH_3_-42), 0.94 (s, 3H, CH_3_-19), 0.76 (d, *J* = 6.5 Hz, 3H, CH_3_-21), 0.70 (s, 3H, CH_3_-18). ^13^C{^1^H} NMR (101 MHz, CDCl_3_): δ 173.8 (C-24), 168.3 (C-41), 160.6 (3-OCHO), 160.5 (12-OCHO), 160.5 (7-OCHO), 143.3 (C-26), 134.0, 128.4, 126.1, 124.8, 124.2, 123.5 (C-30), 118.6, 116.8, 115.6, 75.2 (C-12), 73.7 (C-3), 70.6 (C-7), 57.4, 47.0, 45.5, 44.9, 42.9, 40.8, 37.6, 34.6, 34.5, 34.2, 31.3, 30.8, 30.4, 28.5, 27.1, 26.5, 25.5, 24.0, 22.7, 22.3 (C-19), 17.4 (C-21), 12.0 (C-18). FT-IR (KBr, cm^–1^) ν_max_: 3143 (=C–H), 2941 (C–H), 2869 (C–H), 1715 (C=O). ESI-MS (*m*/*z*): 767 [M + Na]^+^ (100%), 783 [M + K]^+^ (60%), 745 [M + H]^+^ (20%), 843 [M + ClO_4_]^−^, 823 [M + Br]^−^, 779 [M + Cl]^−^. Anal. Calcd for C_41_H_52_N_4_O_9_ (MW = 744.37): C, 66.11; H, 7.04; N, 7.52; O, 19.33. Found: C, 66.14; H, 7.01; N, 7.54; O, 19.31.

##### 4.1.3.4 {1-[(*N*-Acetyl-methylindole)-3-methylene]-1*H*-1,2,3-triazole-4-yl} Methyl 3α-Formyloxy-12α-hydroxycholan-24-oate (**18**)

White oil, yield: 91 mg, 68%, ^1^H NMR (400 MHz, CDCl_3_): δ 8.43 (d, *J* = 8.3 Hz, 1H, 40 H), 8.02 (d, *J* = 0.9 Hz, 1H, 3-HCOO), 7.58 (br s, 1H, 30 H), 7.56 (br s, 1H, 33 H), 7.45–7.38 (m, 2H, 38 H, 39 H), 7.31–7.28 (m, 1H, 37 H), 5.68 (d, *J* = 0.8 Hz, 2H, CH_2_-31), 5.16 (s, 2H, CH_2_-25), 4.88–4.80 (m, 1H, 3β-H), 3.95 (t, *J* = 2.9 Hz, 1H, 12β-H), 2.66 (s, 3H, CH_3_-42), 0.92 (s, 3H, CH_3_-19), 0.90 (d, *J* = 6.3 Hz, 3H, CH_3_-21), 0.63 (s, 3H, CH_3_-18). ^13^C{^1^H} NMR (101 MHz, CDCl_3_): δ 174.0 (C-24), 168.3 (C-41), 160.8 (3-OCHO), 143.4 (C-26), 136.0, 128.4, 126.1, 124.8, 124.2, 123.6 (C-30), 118.6, 116.8, 115.6, 74.2, 73.0, 57.4, 48.2, 47.2, 46.4, 45.5, 41.8, 35.9, 35.0, 34.8, 34.1, 33.6, 32.1, 31.0, 30.7, 28.7, 27.4, 26.9, 26.5, 26.0, 24.0, 23.5, 23.1 (C-19), 17.2 (C-21), 12.6 (C-18). FT-IR (KBr, cm^–1^) ν_max_: 3435 (O–H), 3144 (=C–H), 2936 (C–H), 2868 (C–H), 1716 (C=O). ESI-MS (*m*/*z*): 695 [M + Na]^+^ (100%), 673 [M + H]^+^ (15%), 753 [M + Br]^−^, 707 [M + Cl]^−^. Anal. Calcd for C_39_H_52_N_4_O_6_ (MW = 672.39): C, 69.62; H, 7.79; N, 8.33; O, 14.27. Found: C, 69.59; H, 7.80; N, 8.31; O, 14.30.

##### 4.1.3.5 {1-[(*N*-Acetyl-methylindole)-3-methylene]-1*H*-1,2,3-triazole-4-yl} Methyl 3α-Acetoxy-cholan-24-oate (**19**)

White oil, yield: 80 mg, 60%, ^1^H NMR (400 MHz, CDCl_3_): δ 8.43 (d, *J* = 7.9 Hz, 1H, 40 H), 7.57 (s, 1H, 30 H, 7.54 (bs, 1H, 33 H), 7.45–7.39 (m, 2H, 38 H, 39 H), 7.31–7.28 (m, 1H, 37 H), 5.68 (d, *J* = 0.9 Hz, 2H, CH_2_–31), 5.16 (d, *J* = 1.6 Hz, 2H, CH_2_-25), 4.76–4.68 (m, 1H, 3β-H), 2.66 (s, 3H, CH_3_-42), 2.03 (s, 3H, 3-OCOCH_3_), 0.92 (s, 3H, CH_3_-19), 0.85 (d, *J* = 6.4 Hz, 3H, CH_3_-21), 0.59 (s, 3H, CH_3_-18). ^13^C{^1^H} NMR (101 MHz, CDCl_3_): δ 174.1 (C-24), 170.6 (3-COCH_3_), 168.3 (C-41), 143.4 (C-26), 135.9, 128.3, 126.1, 124.7, 124.2, 123.6 (C-30), 118.6, 116.8, 115.6, 74.4 (C-3), 57.3, 56.4, 55.9, 45.5, 42.7, 41.8, 40.3, 40.1, 35.7, 35.2, 35.0, 34.5, 32.2, 31.0, 30.8, 28.1, 27.0, 26.6, 26.3, 24.1, 24.0, 23.3, 21.5, 20.8 (C-19), 18.2 (C-21), 11.9 (C-18). FT-IR (KBr, cm^–1^) ν_max_: 3147 (=C–H), 2930 (C–H), 2865 (C–H), 1734 (C=O). ESI-MS (*m*/*z*): 693 [M + Na]^+^ (100%), 710 [M + K]^+^ (50%), 671 [M + H]^+^ (10%), 705 [M + Cl]^−^. Anal. Calcd for C_40_H_54_N_4_O_5_ (MW = 670.41): C, 71.61; H, 8.11; N, 8.35; O, 11.92. Found: C, 71.64; H, 8.12; N, 8.37; O, 11.87.

##### 4.1.3.6 {1-[(*N*-Acetyl-methylindole)-3-methylene]-1*H*-1,2,3-triazole-4-yl} Methyl 3α,12α-Diacetoxy-cholan-24-oate (**20**)

White oil, yield: 124 mg, 85%, ^1^H NMR (400 MHz, CDCl_3_): δ 8.43 (d, *J* = 8.2 Hz, 1H, 40 H), 7.57 (d, *J* = 7.7 Hz, 2H, 30 H, 33 H), 7.45–7.38 (m, 2H, 38 H, 39 H), 7.30–29 (m, 1H, 37 H), 5.69 (s, 2H, CH_2_-31), 5.16 (s, 2H, CH_2_-25), 5.04 (t, *J* = 2.9 Hz, 1H, 12β-H), 4.74–4.66 (m, 1H, 3β-H), 2.66 (s, 3H, CH_3_-42), 2.08 (s, 3H, 12-OCOCH_3_), 2.04 (s, 3H, 3-OCOCH_3_), 0.90 (s, 3H, CH_3_-19), 0.73 (d, *J* = 6.4 Hz, 3H, CH_3_-21), 0.67 (s, 3H, CH_3_-18). ^13^C{^1^H} NMR (101 MHz, CDCl_3_): δ 173.9 (C-24), 170.6 (3-COCH_3_), 170.4 (12-COCH_3_), 168.3 (C-41), 143.3 (C-26), 135.9, 128.4, 126.1, 124.8, 124.2, 123.6 (C-30), 118.6, 116.8, 115.6, 75.8 (C-3), 74.2 (C-12), 57.4, 49.3, 47.4, 45.5, 44.9, 41.8, 35.6, 34.7, 34.5, 34.3, 34.0, 32.2, 30.9, 30.6, 27.2, 26.8, 26.6, 25.6, 25.6, 24.0, 23.3, 23.0, 21.4, 21.4 (C-19), 17.4 (C-21), 12.3 (C-18). FT-IR (KBr, cm^–1^) ν_max_: 3121 (=C–H), 2930 (C–H), 2868 (C–H), 1735 (C=O). ESI-MS (*m*/*z*): 751 [M + Na]^+^ (100%), 767 [M + K]^+^ (10%), 808 [M + Br]^−^, 763 [M + Cl]^−^. Anal. Calcd for C_42_H_56_N_4_O_7_ (MW = 728.41): C, 69.21; H, 7.74; N, 7.69; O, 15.36. Found: C, 69.17; H, 7.73; N, 7.72; O, 15.38.

##### 4.1.3.7 {1-[(*N*-Acetyl-methylindole)-3-methylene]-1*H*-1,2,3-triazole-4-yl} Methyl 3α,7α,12α-Triacetoxycholan-24-oate (**21**)

White oil, yield: 138 mg, 88%, ^1^H NMR (400 MHz, CDCl_3_): δ 8.43 (d, *J* = 8.6 Hz, 1H, 40 H), 7.58 (br s, 1H, 30 H, 7.56 (bs, 1H, 33 H), 7.45–7.38 (m, 2H, 38 H, 39 H), 7.30–7.29 (m, 1H, 37 H), 5.68 (s, 2H, CH_2_–31), 5.15 (s, 2H, CH_2_-25), 5.04 (t, *J* = 3.0 Hz, 1H, 12β-H), 4.90 (d, *J* = 9.5 Hz, 1H, 7β-H), 4.61–4.53 (m, 1H, 3β-H), 2.66 (s, 3H, CH_3_-42), 2.12 (s, 3H, 7-OCOCH_3_), 2.09 (s, 3H, 12-OCOCH_3_), 2.05 (s, 3H, 3-OCOCH_3_), 0.91 (s, 3H, CH_3_-19), 0.74 (d, *J* = 6.4 Hz, 3H, CH_3_-21), 0.68 (s, 3H, CH_3_-18); ^13^C{^1^H} NMR (101 MHz, CDCl_3_): δ 173.9 (C-24), 170.5 (3-COCH_3_), 170.5 (12-COCH_3_), 170.4 (7-COCH_3_), 168.3 (C-41), 143.3 (C-26), 136.0, 128.4, 126.1, 124.8, 124.2, 123.5 (C-30), 118.6, 116.8, 115.6, 75.3 (C-3), 74.1 (C-12), 70.6 (C-7), 57.3, 47.2, 45.5, 43.3, 40.9, 37.7, 34.7, 34.6, 34.4, 34.3, 31.2, 30.8, 30.5, 28.8, 27.1, 26.9, 25.5, 24.0, 22.7, 22.5, 21.6, 21.5, 21.4 (C-19), 17.4 (C-21), 12.1 (C-18). FT-IR (KBr, cm^–1^) ν_max_: 3141 (=C–H), 2961 (C–H), 2871 (C–H), 1732 (C=O). ESI-MS (*m*/*z*): 809 [M + Na]^+^ (100%), 825 [M + K]^+^ (50%), 787 [M + H]^+^ (20%), 866 [M + Br]^−^, 821 [M + Cl]^−^. Anal. Calcd for C_44_H_58_N_4_O_9_ (MW = 786.42): C, 67.15; H, 7.43; N, 7.12; O, 18.30. Found: C, 67.17; H, 7.40; N, 7.14; O, 18.29.

Synthesis of {1-[(*N*-acetyl-methylindole)-3-methylene]-1*H*-1,2,3-triazole-4-yl} methyl 3α,7α,12α-triacetoxycholan-24-oate (**21**)—The experiment was carried out on a larger scale.

##### 4.1.3.8 Propargyl 3α,7α,12α-Triacetyl-5β-cholan-24-oate (**14**)

2 mmol (1.11 g) was dissolved in *t*-BuOH/MeOH (5:1), (40 mL) at 60 °C using a water bath. Then, *N*-acethyl-3-azidomethylindole (**4**) (2 mmol, 428 mg) was dissolved in *t*-BuOH/MeOH (5:1) (20 mL). CuSO_4_·5H_2_O (0.0002 mmol, 50 mg) and sodium ascorbate (0.0007 mmol, 139 mg) in 10 mL of distilled water were added. The mixture was stirred on a magnetic stirrer and heated in a water bath at 60 °C. Subsequent portions of the catalyst were added until the aqueous layer of the reaction mixture turned bluish-green. The reaction was monitored by TLC (PhMe/EtOAc 1:1). An orange solid precipitated as the reaction mixture was stirred. The reaction mixture was extracted with chloroform. The organic layer was evaporated under reduced pressure. The crude product was purified over column chromatography (gradient elution, starting from CHCl_3_/EtOAc 5:1). 944 mg portion of pure product (60%) was obtained as a white oil.

### 4.2 PM5 Calculations

PM5 semiempirical calculations were performed by using the WinMopac 2003 program.

### 4.3 Biological Activity

#### 4.3.1 Human Red Blood Cells

Freshly human RBC suspensions were purchased from the blood bank in Pozna according to the bilateral agreement between Adam Mickiewicz University and blood bank no. ZP/2867/D/21 without any contact with blood donors. RBCs were washed three times (3000 rpm, 10 min, 4 °C) in 7.4 pH phosphate buffered saline (PBS—137 mM NaCl, 2.7 mM KCl, 10 mM Na_2_HPO_4_, 1.76 mM KH_2_PO_4_) supplemented with 10 mM glucose. After washing, RBC was suspended in the PBS buffer at 1.65 × 10^9^ cells/mL, stored at 4 °C, and used within 5 h.

#### 4.3.2 Hemolysis Assay

RBCs (1.65 × 10^8^ cells/mL, ∼1.5% hematocrit) were incubated in PBS (7.4 pH) supplemented with 10 mM glucose and containing derivatives tested at a 0.1 mg/mL concentration for 60 min at 37 °C in a thermo-shaker. RBCs incubated in PBS without compounds tested were taken as the negative controls, and RBCs incubated in ice-cold deionized water were taken as the positive controls. Each sample was prepared in triplicate, and the experiments were repeated three times (*n* = 9). After incubation, the RBC suspensions were centrifuged (3000 rpm, 10 min, 4 °C) and the absorbance of the supernatants at 540 nm was measured. The results were expressed as a percentage (%) of hemolysis, which was calculated using the following formula
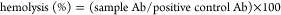
where sample Ab is the absorbance value of the supernatant of RBC incubated with compounds tested, and positive control, AB is an absorbance value of the supernatant of RBC incubated in ice-cold deionized water. Each sample was prepared in triplicate, and the results are presented as a mean value (±SD) of three independent experiments (*n* = 9).

#### 4.3.3 Erythrocyte Shape Evaluation

The incubation was carried out as follows: RBC was fixed in the mixture of 5% paraformaldehyde (PFA) and 0.01% glutaraldehyde (GA) for 60 min at room temperature. After fixation, cells were washed by exchanging the supernatant with PBS buffer, settled on poly-l-lysine-treated (0.1 mg/mL, 10 min, room temperature) cover glasses (15 min, room temperature), and mounted 80% glycerol. The coverslips were sealed with nail polish. Many cells in several separate experimental samples were studied using a RED-233 MOTIC microscope (63× objective, 10× ocular). Images were acquired using a Motica 3.0 MP microscopic camera and the program Motic Images Plus 3.0. The shapes of RBC in every sample were estimated according to the Bessis classification.^41^

#### 4.3.4 Statistical Analysis

For hemolytic activity, data were plotted as the arithmetic mean ± standard deviation (SD) of the results of three independent experiments, with every sample (test samples and positive controls) in triplicate (*n* = 9).

### 4.4 Molecular Docking

The molecular docking procedure was carried out using the rdkit tool,^42^ which allows for the construction of 3D structures from SMILES representations of structures. The 3D structures were saved in *.pdb format and then converted to the *.pdbqt format required by the AutoDock Vina algorithm.^43^ The OpenBabel program was used to convert the files.^44,45^ The receptors were prepared with the help of AutoDock Tools 1.5.7.^46,47^ The molecular docking method was carried out using the AutoDock Vina multiple CPU technique.^43^ The visualizations of the molecular docking’s best ligand’s poses along with the possible H-bond formation have been done with the Chimera tool (1.16).^48^ The 2D ligand–protein domain interactions graphs have been prepared with the application of ProteinsPlus.^49−54^

2Q85 and 5V5Z (PDB IDs) were chosen as receptors for the molecular docking of structures **16**, **18**, and **20** (see Figure 4). These are obtained from the Protein Data Bank (PDB).^55^ The former is attributed to antibacterial action (*Escherichia coli* MurB),^56^ whereas the latter is attributed to antifungal activity (CYP51_ca_).^57^

Docked structures share the same active site of the protein domain as cocrystallized ligands. The search parameters for 2Q85’s search parameters were center (*x*, *y*, *z*): 13.853, 0.000, 0.000, and size (*x*, *y*, *z*) (80 × 80 × 80) Å^3^. 5V5Z had the center (*x*, *y*, *z*) of −41.500, −11.400, 23.547, and the size (*x*, *y*, *z*) of (70 × 100 × 60) Å^3^.

## Data Availability

The data underlying this study are available in the published article and its Supporting Information.

## Supporting Information Available

General information and copies of ESI-MS and ^1^H and ^13^C NMR spectra (PDF)
