# Supplementary material for: Synthesis and Hemolytic Activity of Bile Acid-Indole Bioconjugates Linked by Triazole
Source: J Org Chem. 2023 Dec 7;88(24):16719–34. doi: 10.1021/acs.joc.3c00815 (PMC10729025; doi:10.1021/acs.joc.3c00815)
Supplement: Supplementary file 1 — jo3c00815_si_001.pdf [file jo3c00815_si_001.pdf]

Supporting information

Synthesis and hemolytic activity of bile acids-indole bioconjugates linked by triazole

Natalia Berdzik[a], Hanna Koenig[a], Lucyna Mrówczyńska[b], Damian Nowak[c], Beata Jasiewicz[a]* and Tomasz Pospieszny[a]*

[a]*Department of Bioactive Products, Faculty of Chemistry, Adam Mickiewicz University, Uniwersytetu Poznańskiego 8, 61-614 Poznań, Poland*

[b]*Department of Cell Biology, Faculty of Biology, Adam Mickiewicz University, Uniwersytetu Poznańskiego 6, 61-614 Poznań, Poland*

[c]*Department of Quantum Chemistry, Faculty of Chemistry, Adam Mickiewicz University in Poznan, Uniwersytetu Poznanskiego 8, 61-614 Poznan, Poland*

*Corresponding Authors: beata.jasiewicz@amu.edu.pl and tposp@amu.edu.pl

Table of Contents

1. General information S2
2. Copies of ESI-MS spectra S2–S6
3. Copies of NMR spectra S6-S19

1. General Information

All of the synthesis reagents were purchased from Sigma-Aldrich. Solvents chloroform, dichloromethane, toluene, dimethylformamide, dimethylsulfoxide, tetrahydrofuran, ethyl acetate, and methanol were obtained from commercial sources (Merck, Fisher) and used without purification. IR Spectra: FT/IR Nicolet iS5 (KBr pellet, cm^{-1}) ^{1}H and ^{13}C NMR spectra: Bruker Avance 600 MHz, Varian VNMR-S 400 MHz and Varian Mercury 300 MHz spectrometer (Oxford, UK). EI-MS spectra: AMD Intectra Mass AMD 402 spectrometer ESI-MS spectra: Waters HPLC/MS chromatograph. The elemental analysis was performed on the Elemental Analyzer Vario EL III apparatus, examining the percentage content of nitrogen, carbon and hydrogen.

2. Copies of ESI-MS spectra

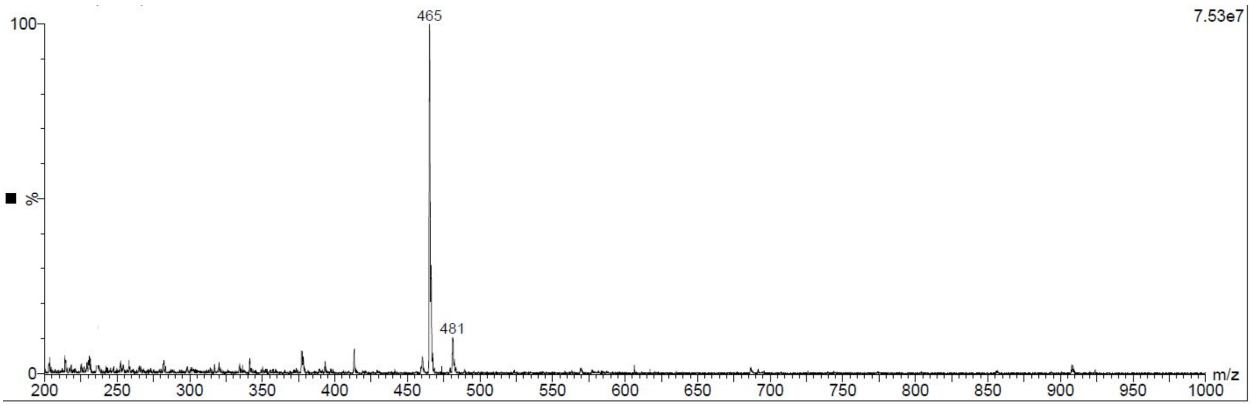

ESI-MS spectra of compound **8**

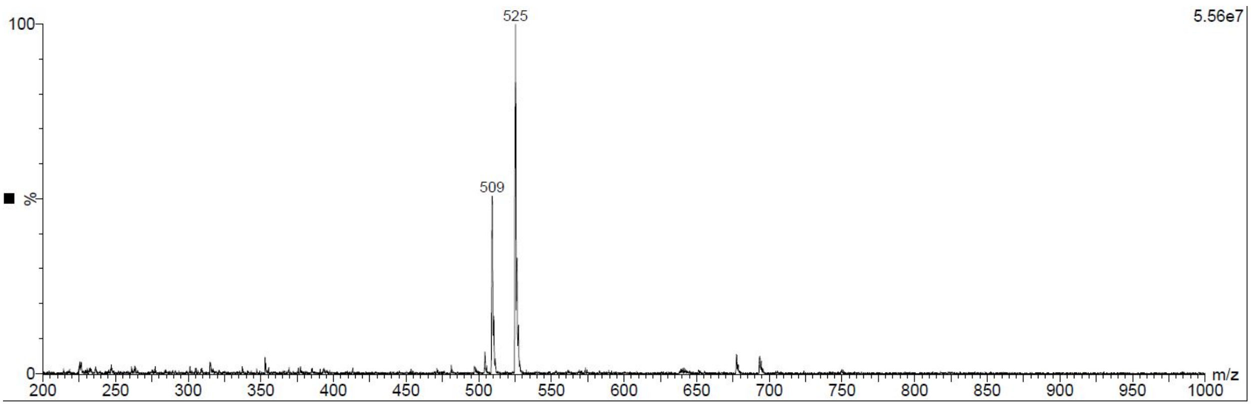

ESI-MS spectra of compound **9**

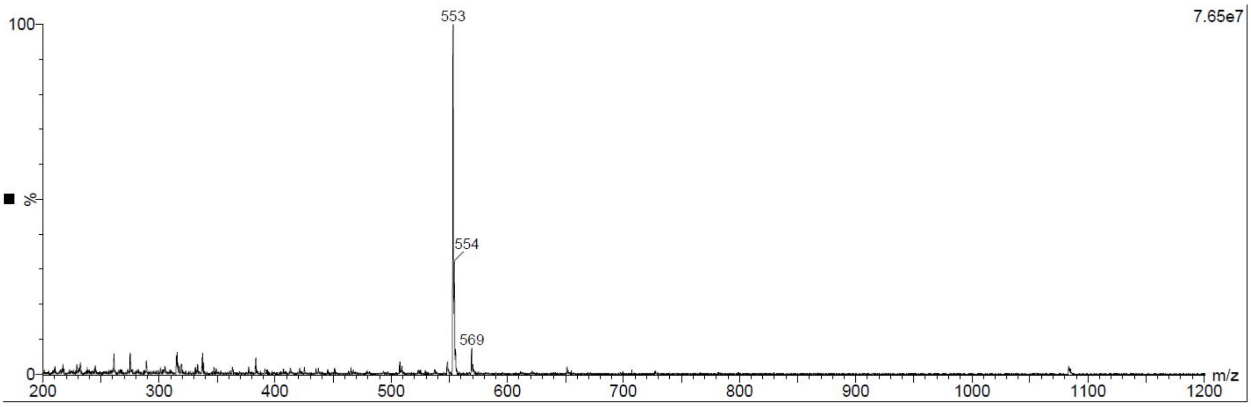

ESI-MS spectra of compound **10**

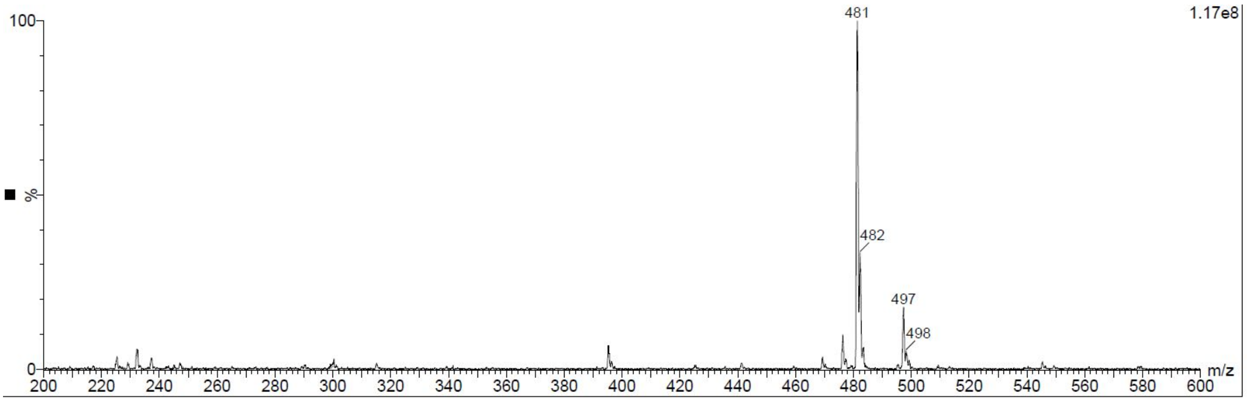

ESI-MS spectra of compound **11**

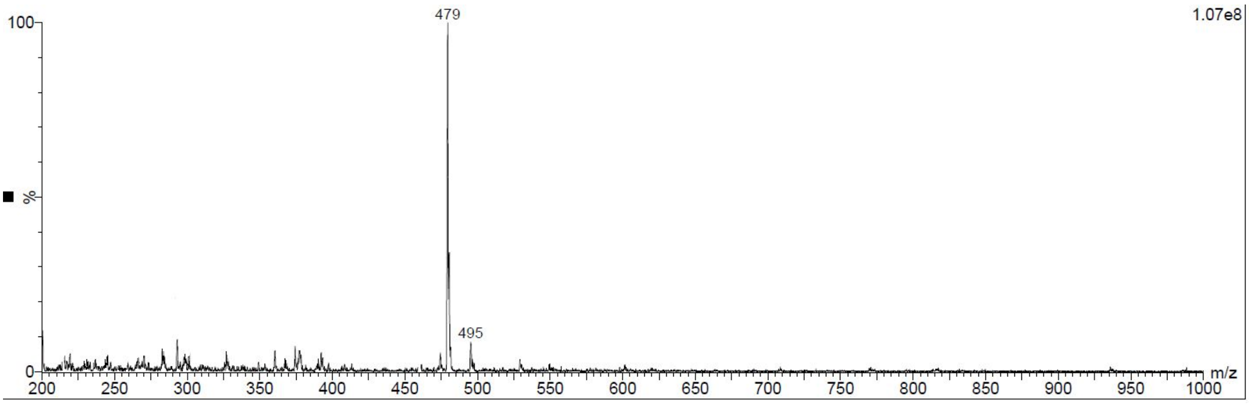

ESI-MS spectra of compound **12**

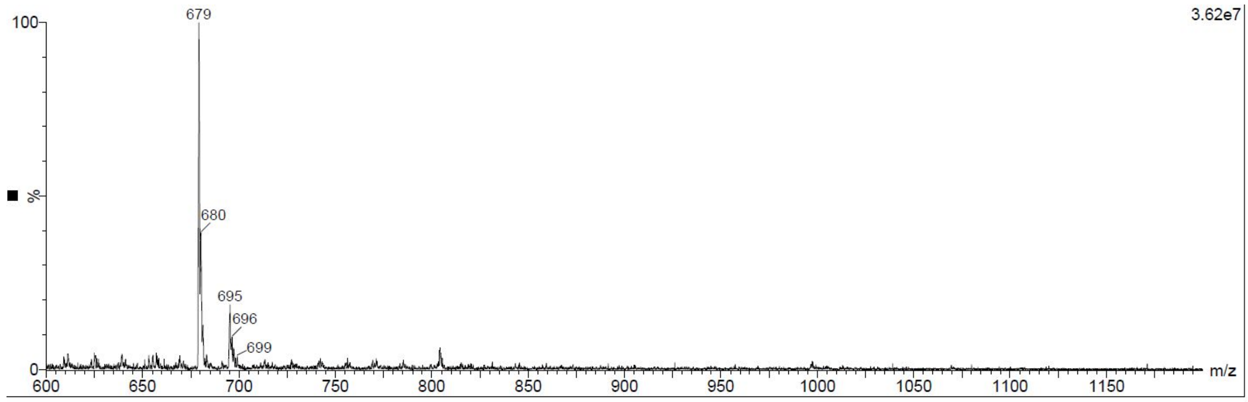

ESI-MS spectra of compound **15**

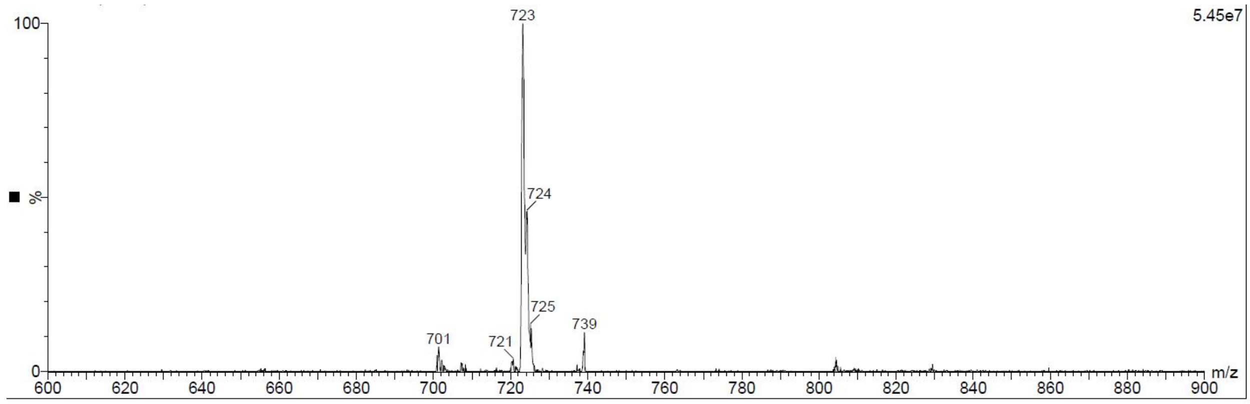

ESI-MS spectra of compound **16**

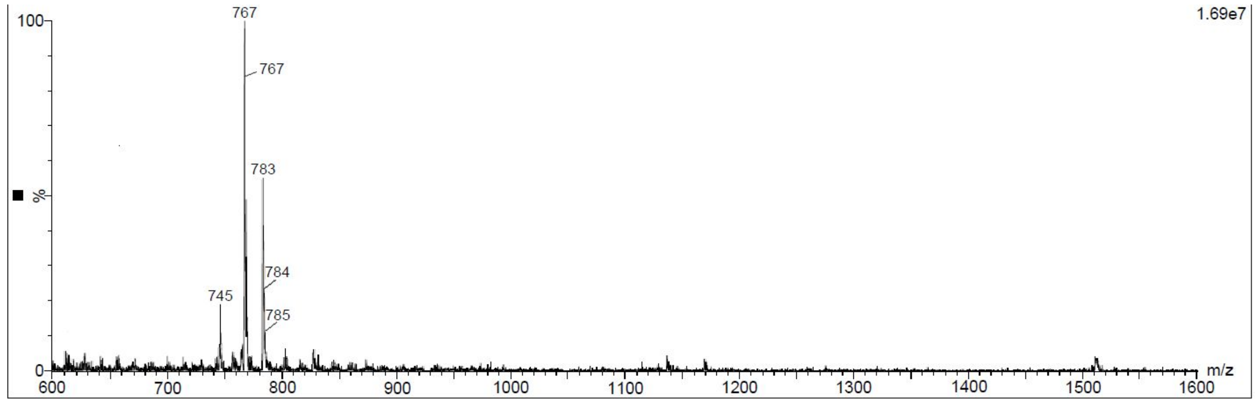

ESI-MS spectra of compound **17**

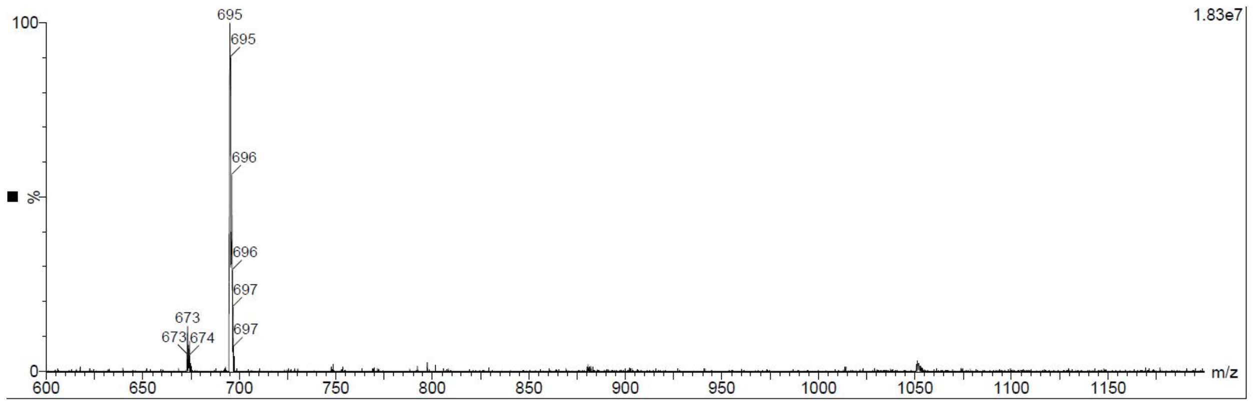

ESI-MS spectra of compound **18**

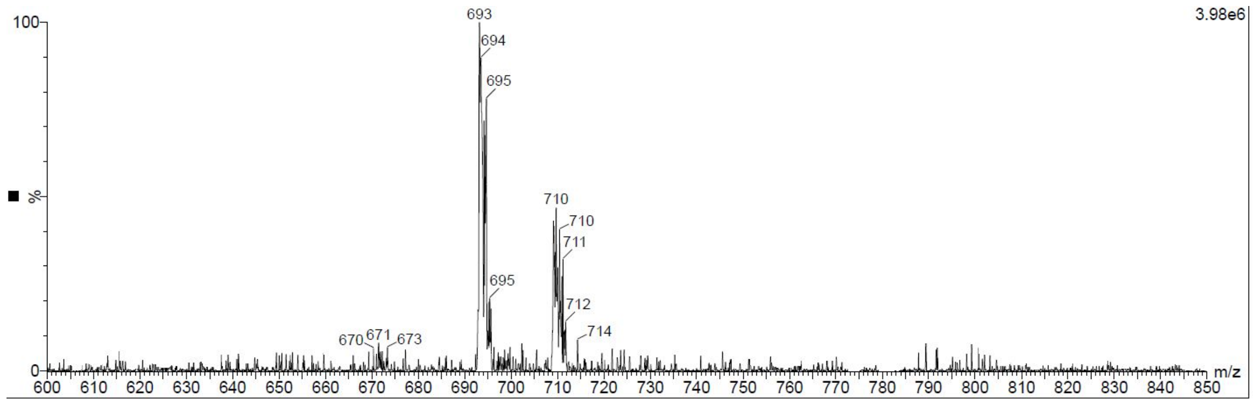

ESI-MS spectra of compound **19**

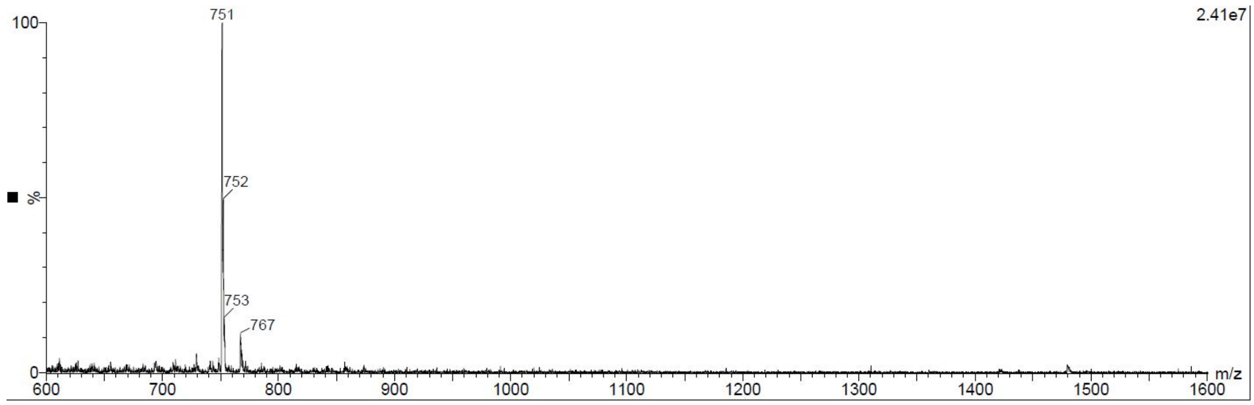

ESI-MS spectra of compound **20**

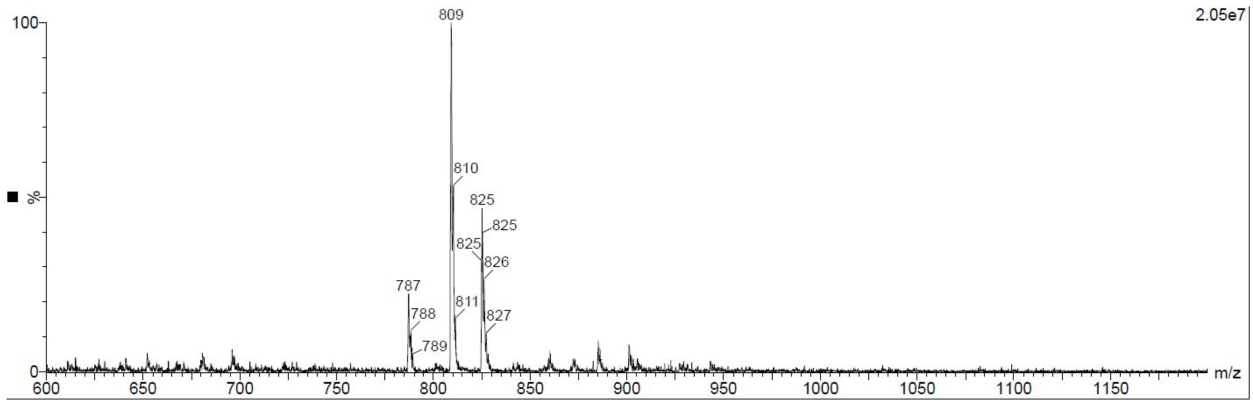

ESI-MS spectra of compound **21**

4. Copies of NMR spectra

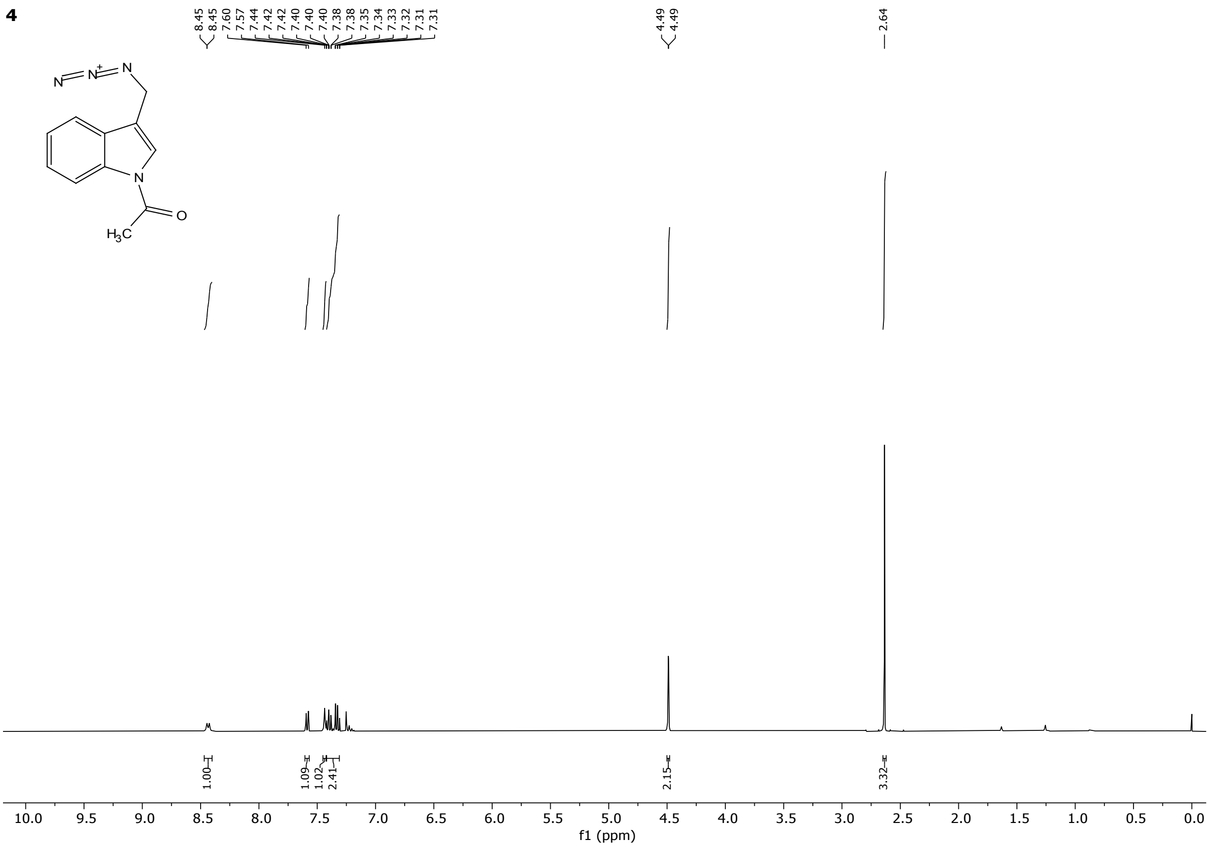

^{1}H NMR (400 MHz, $CDCl_3$) spectra of compound **4**

4

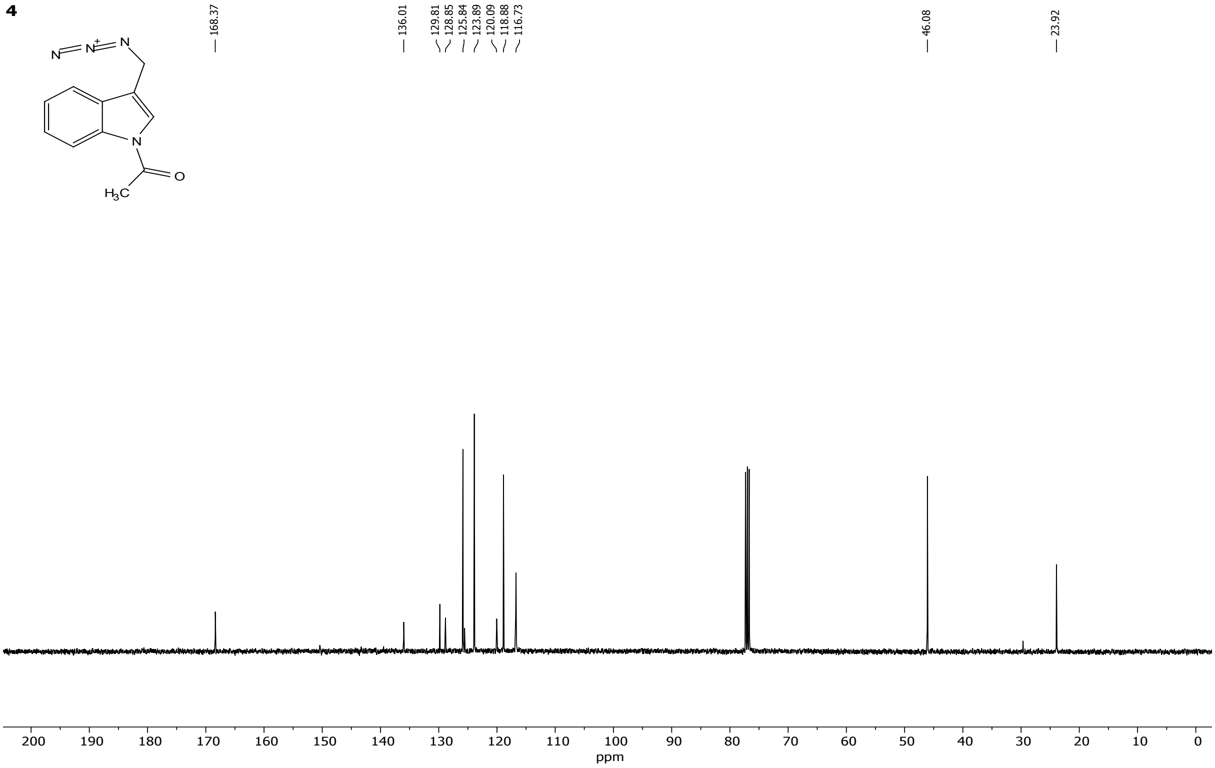

$^{13}C\{^1H\}$ NMR (101 MHz, $CDCl_3$) spectra of compound **4**

8

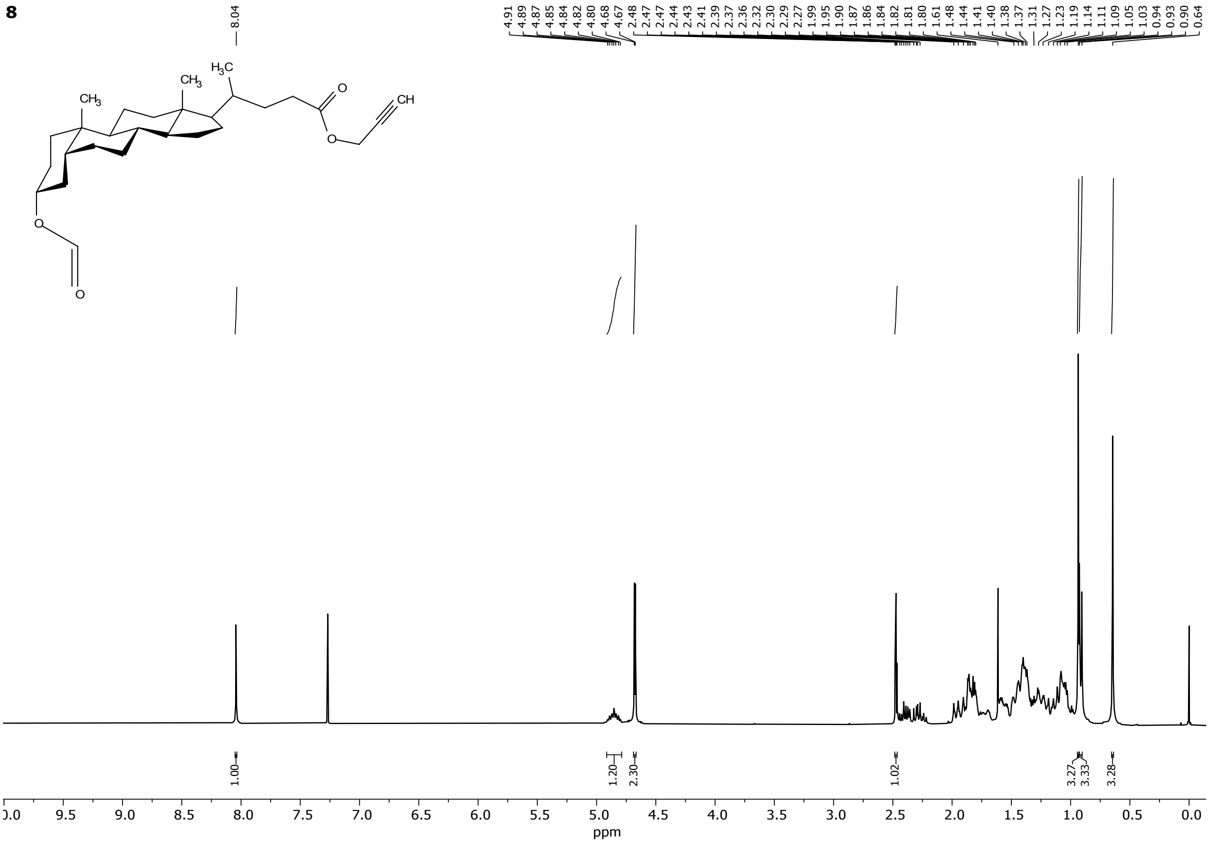

^1H NMR (300 MHz, $CDCl_3$) spectra of compound **8**

8

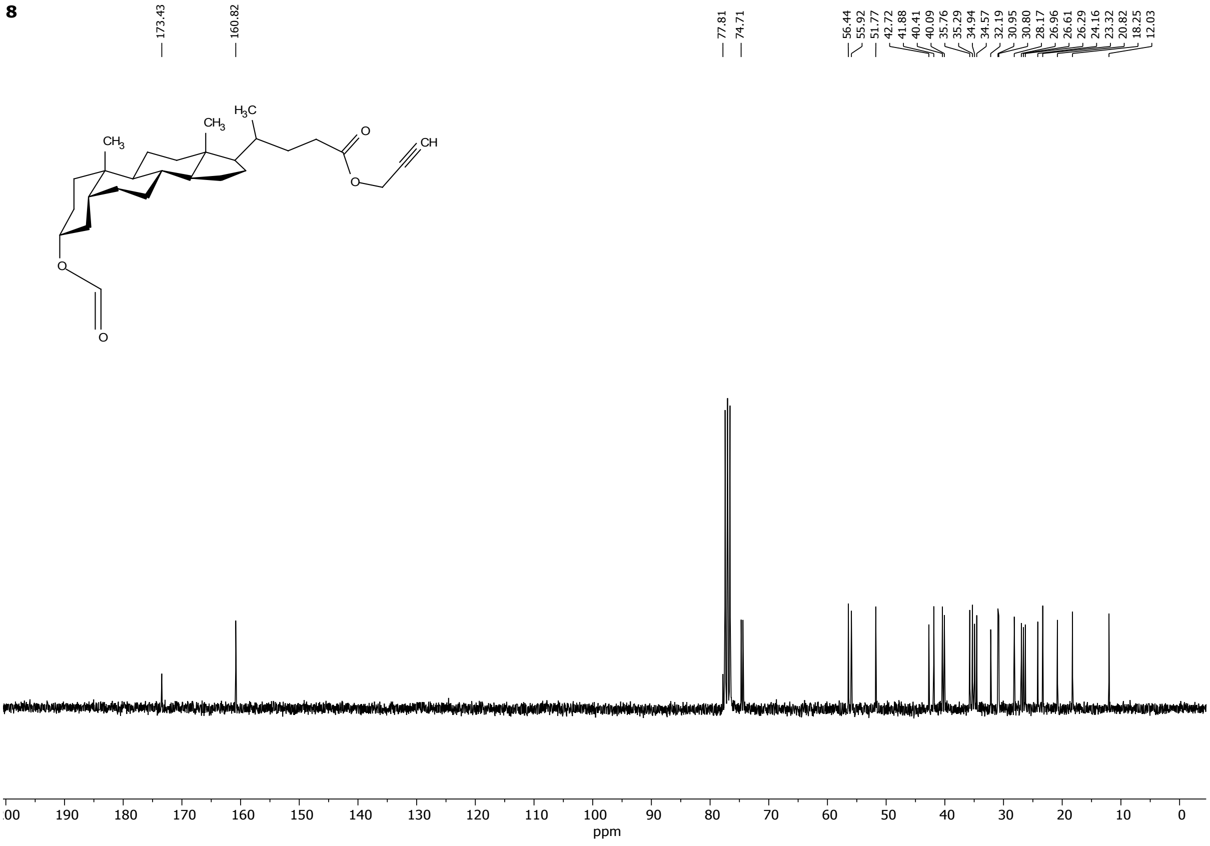

$^{13}C\{^{1}H\}$ NMR (76 MHz, $CDCl_3$) spectra of compound **8**

9

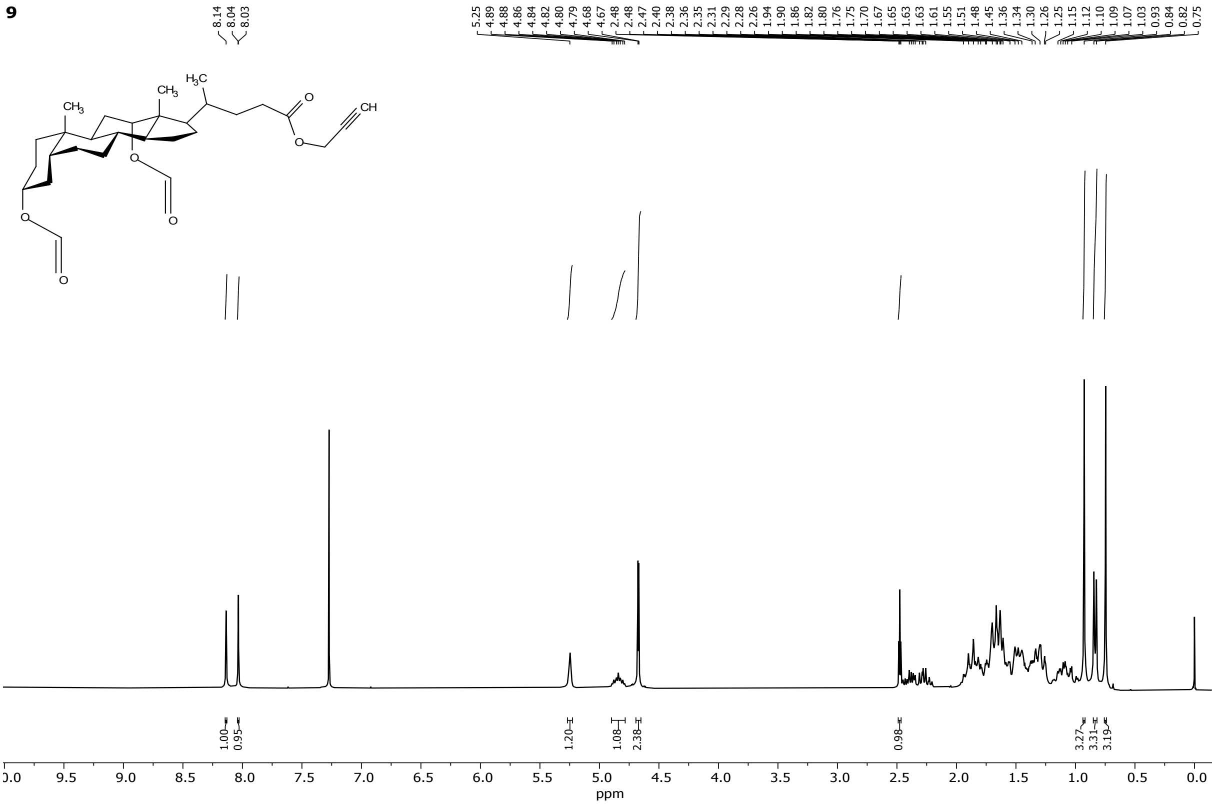

^{1}H NMR (300 MHz, $CDCl_3$) spectra of compound **9**

9

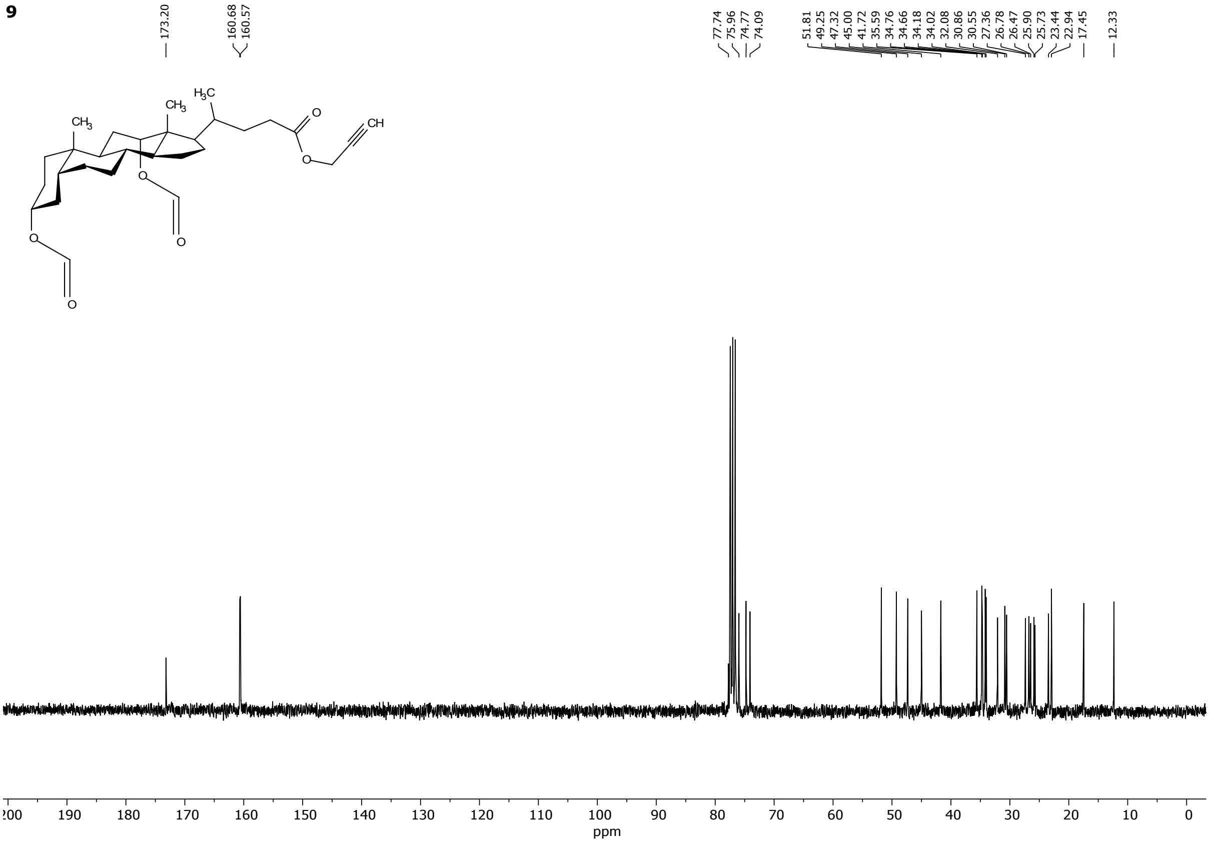

$^{13}C\{^{1}H\}$ NMR (76 MHz, $CDCl_3$) spectra of compound **9**

10

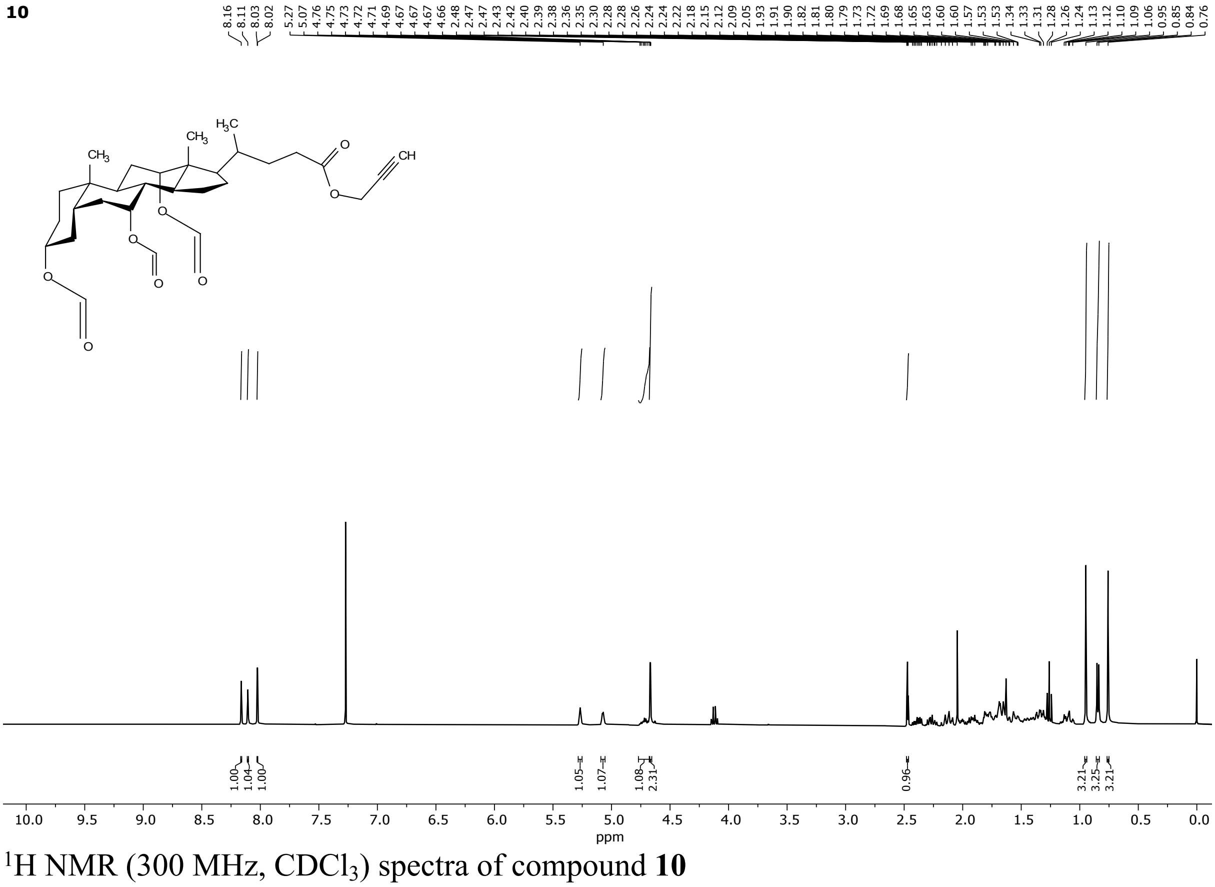

^{1}H NMR (300 MHz, $CDCl_3$) spectra of compound **10**

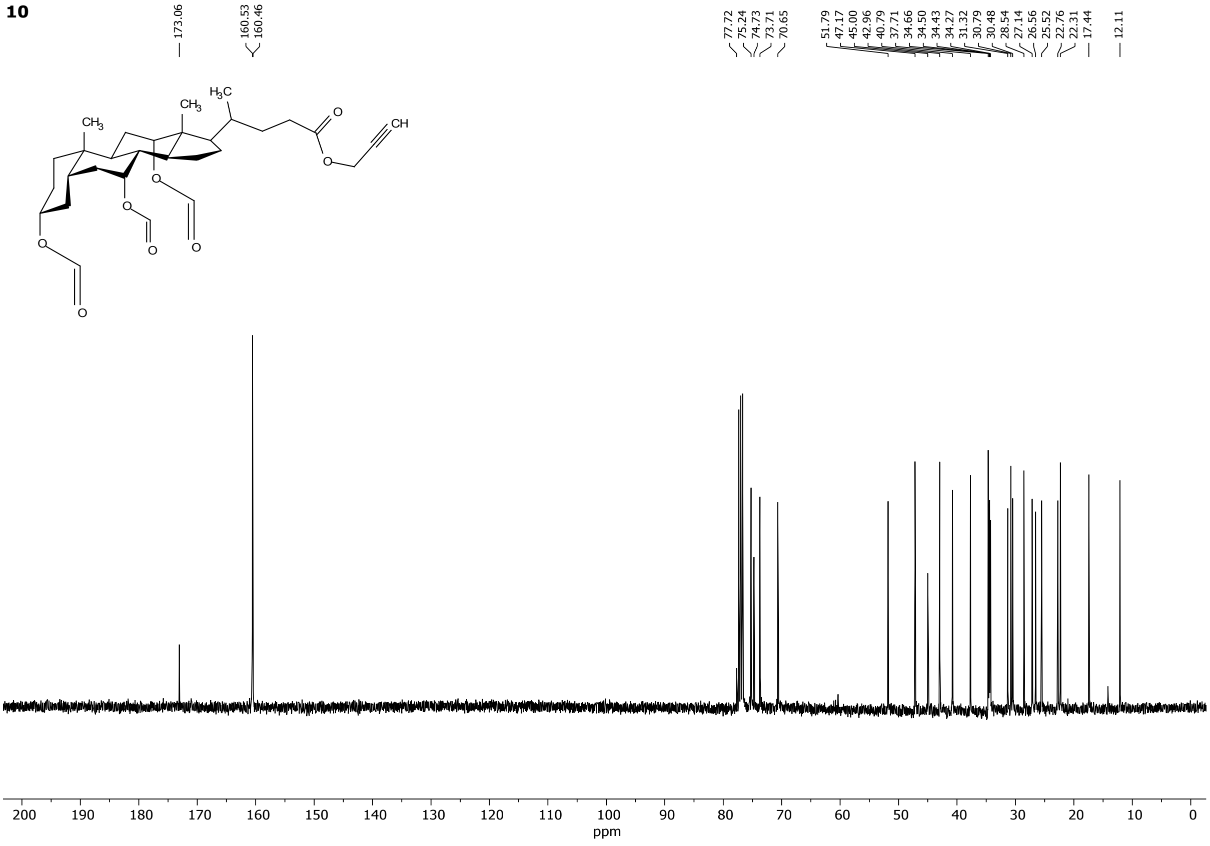

$^{13}C\{^{1}H\}$ NMR (76 MHz, $CDCl_3$) spectra of compound **10**

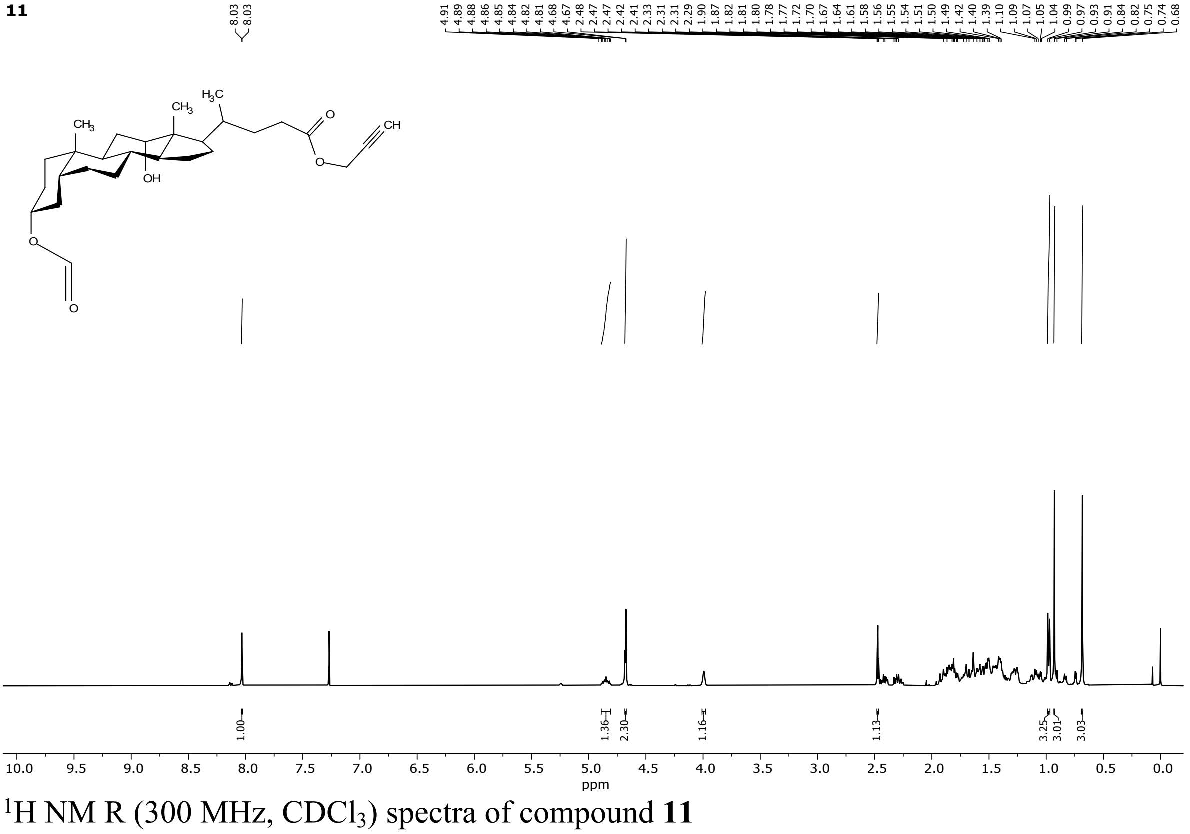

^{1}H NM R (300 MHz, $CDCl_3$) spectra of compound **11**

11

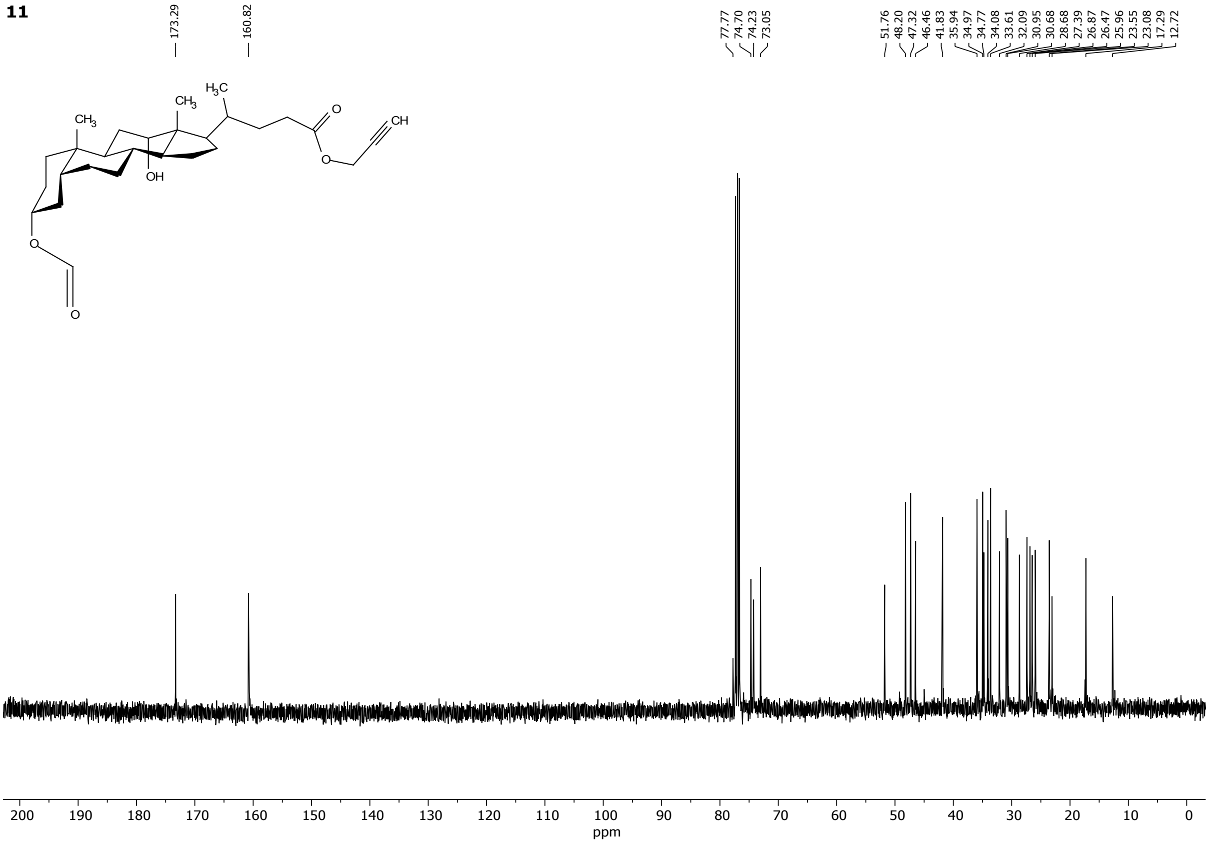

$^{13}C\{^{1}H\}$ NMR (76 MHz, $CDCl_3$) spectra of compound **11**

12

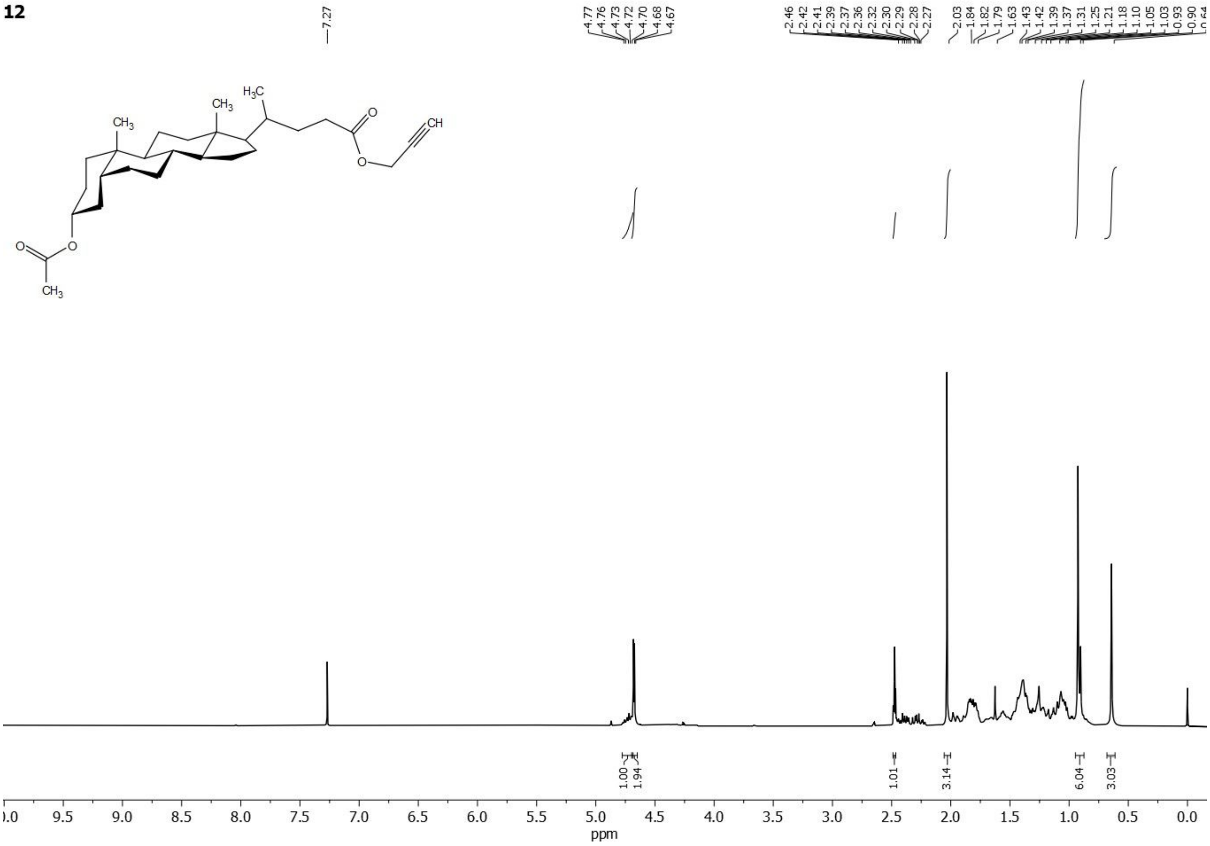

^{1}H NMR (300 MHz, $CDCl_3$) spectra of compound **12**

12

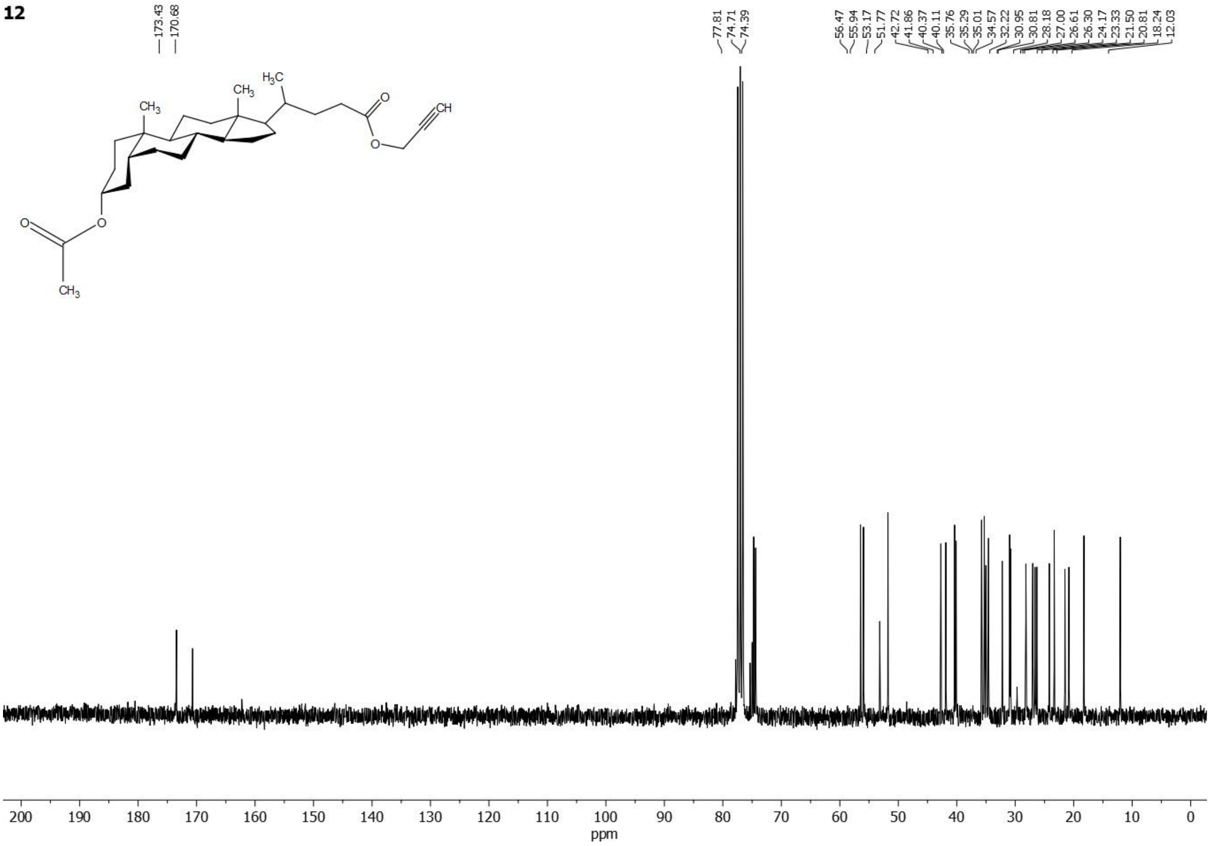

$^{13}C\{^{1}H\}$ NMR (76 MHz, $CDCl_3$) spectra of compound **12**

15

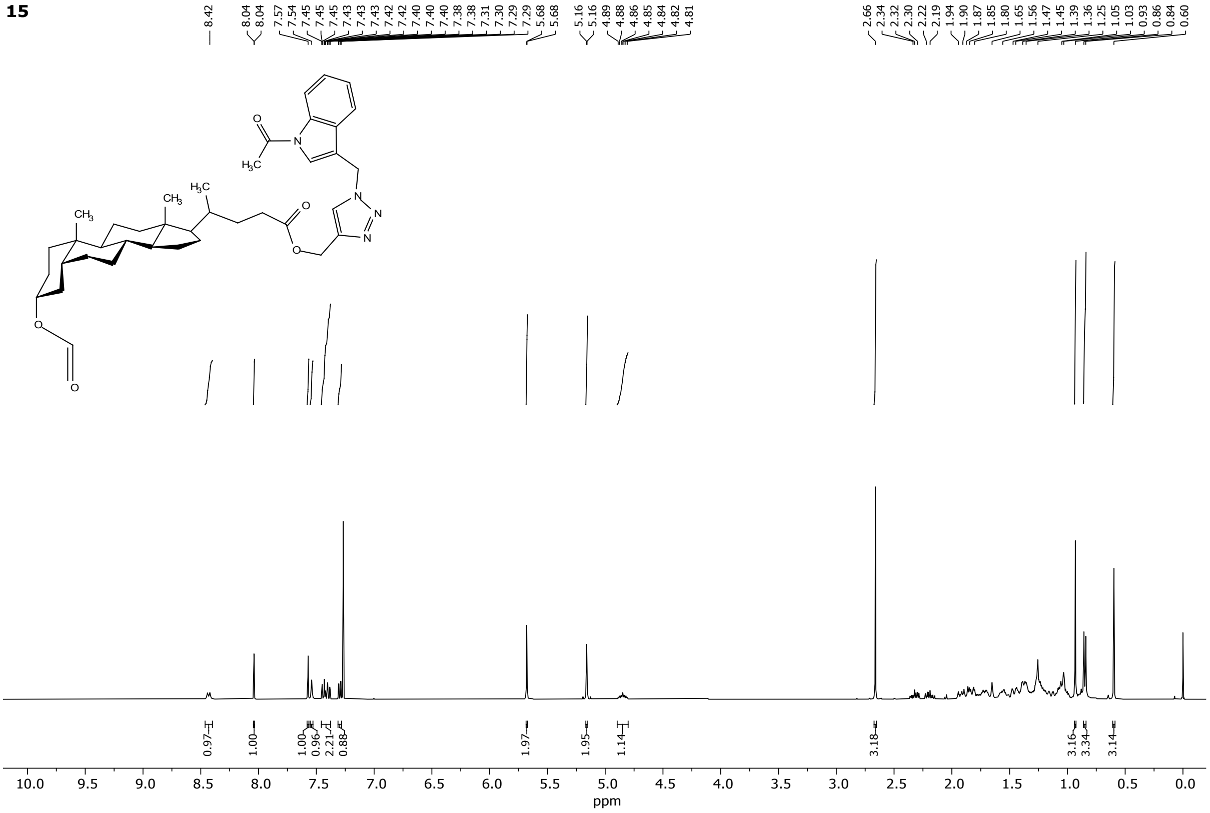

^{1}H NMR (400 MHz, $CDCl_3$) spectra of compound **15**

15

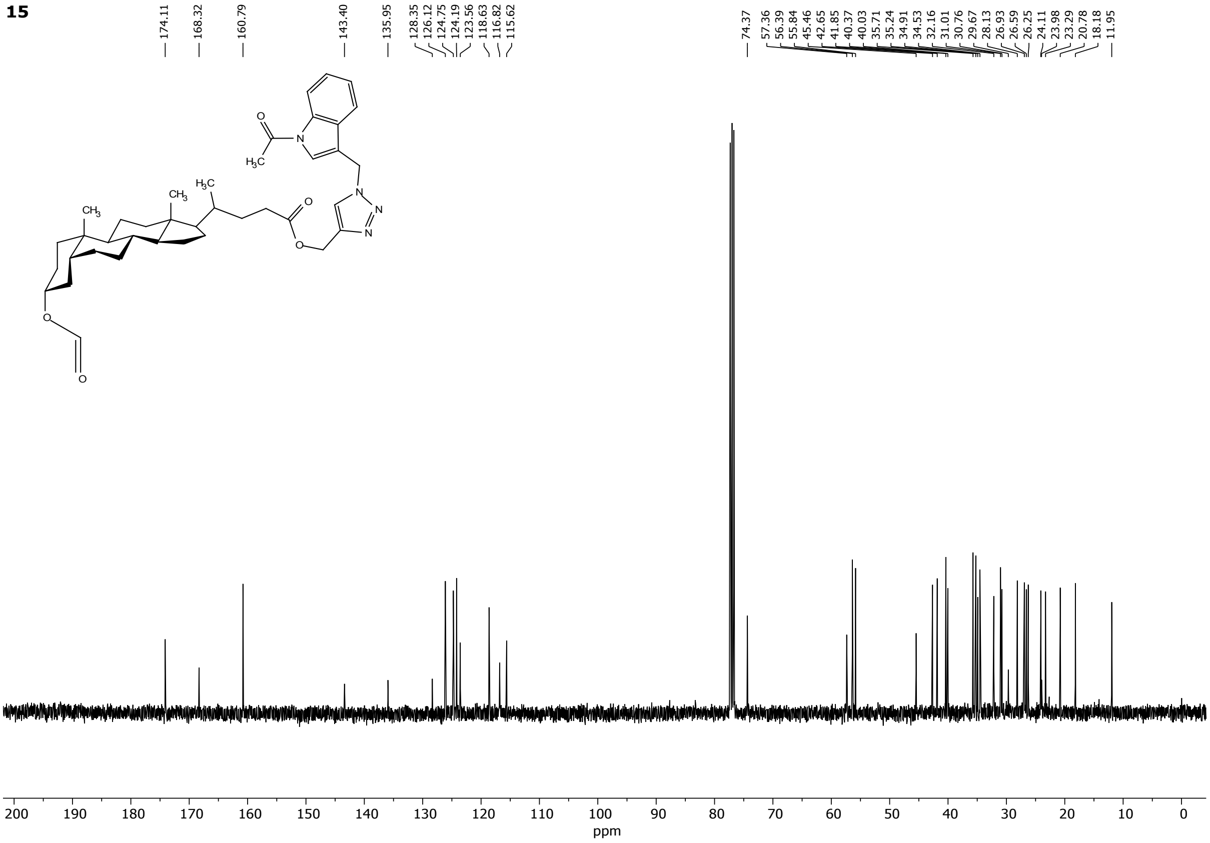

$^{13}C\{^1H\}$ NMR (101 MHz, $CDCl_3$) spectra of compound **15**

16

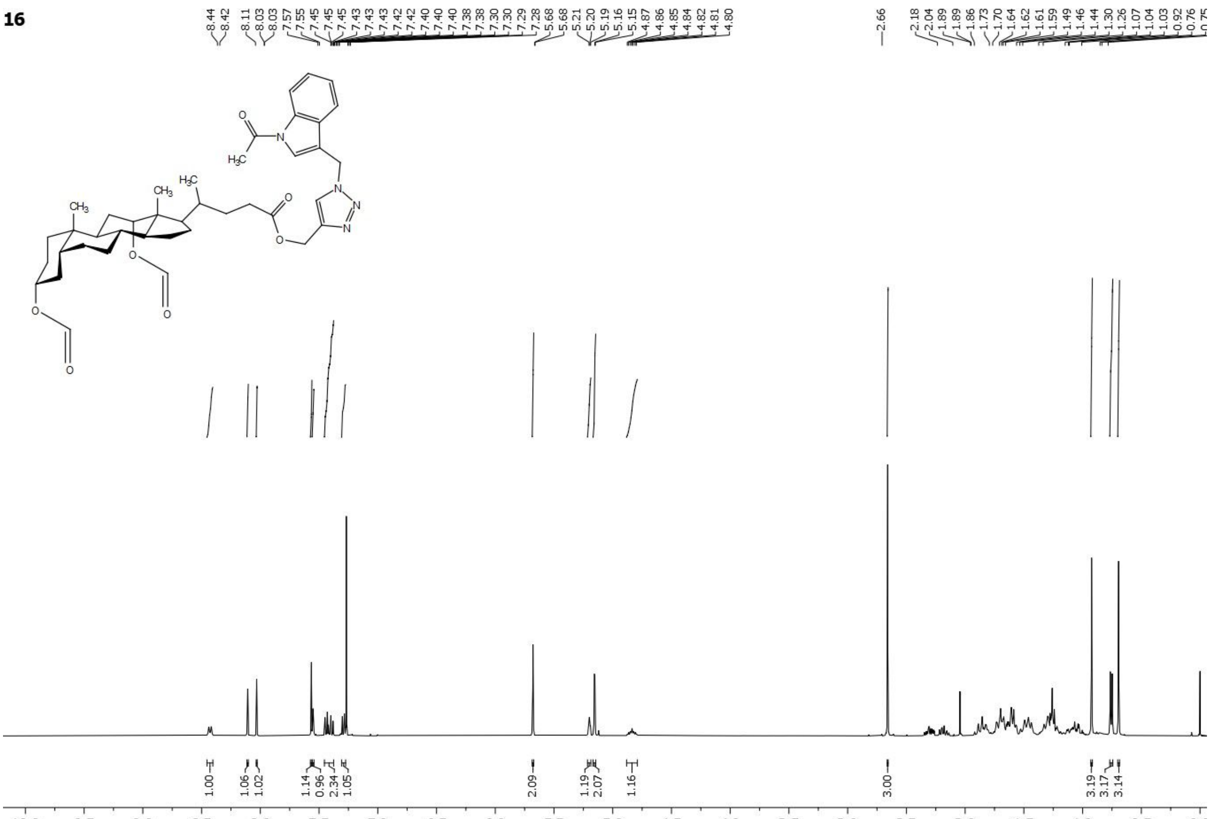

^1H NMR (400 MHz, $CDCl_3$) spectra of compound **16**

16

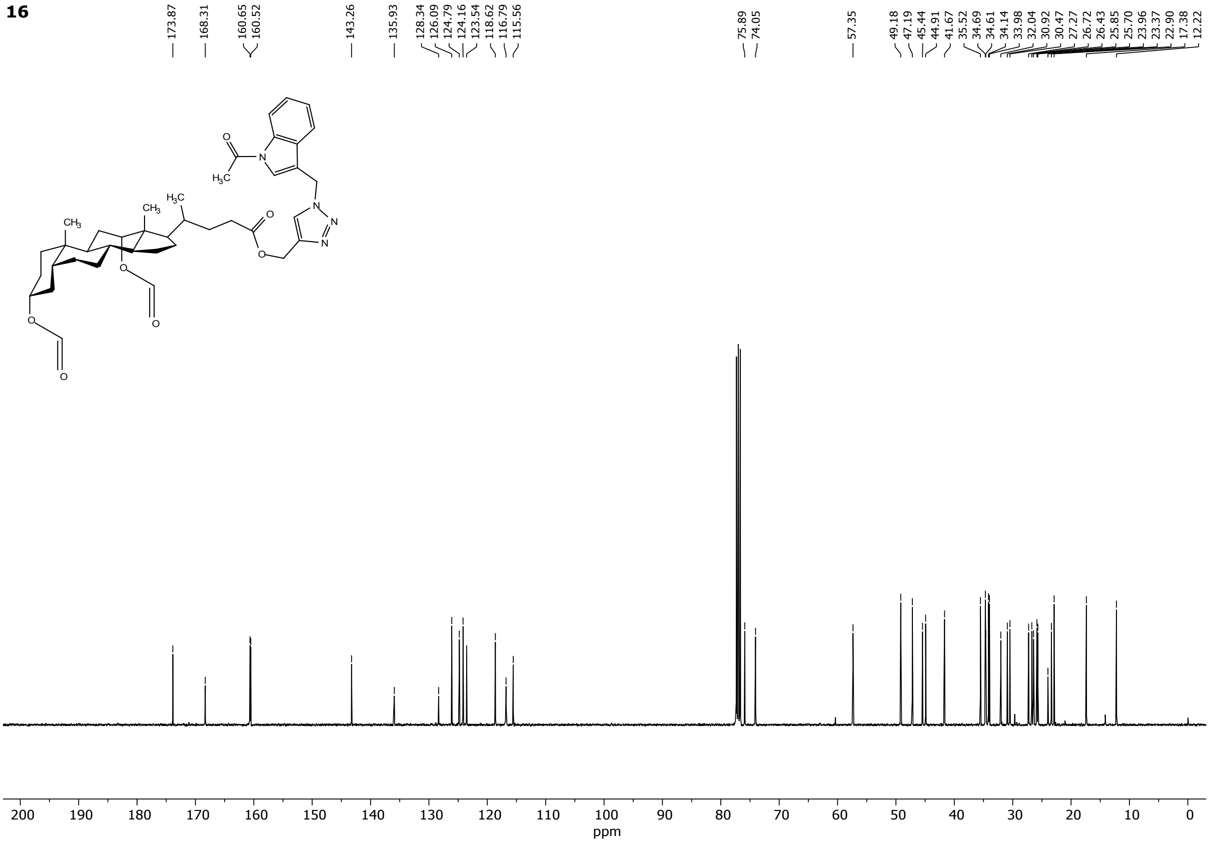

$^{13}C\{^{1}H\}$ NMR (101 MHz, $CDCl_3$) spectra of compound **16**

17

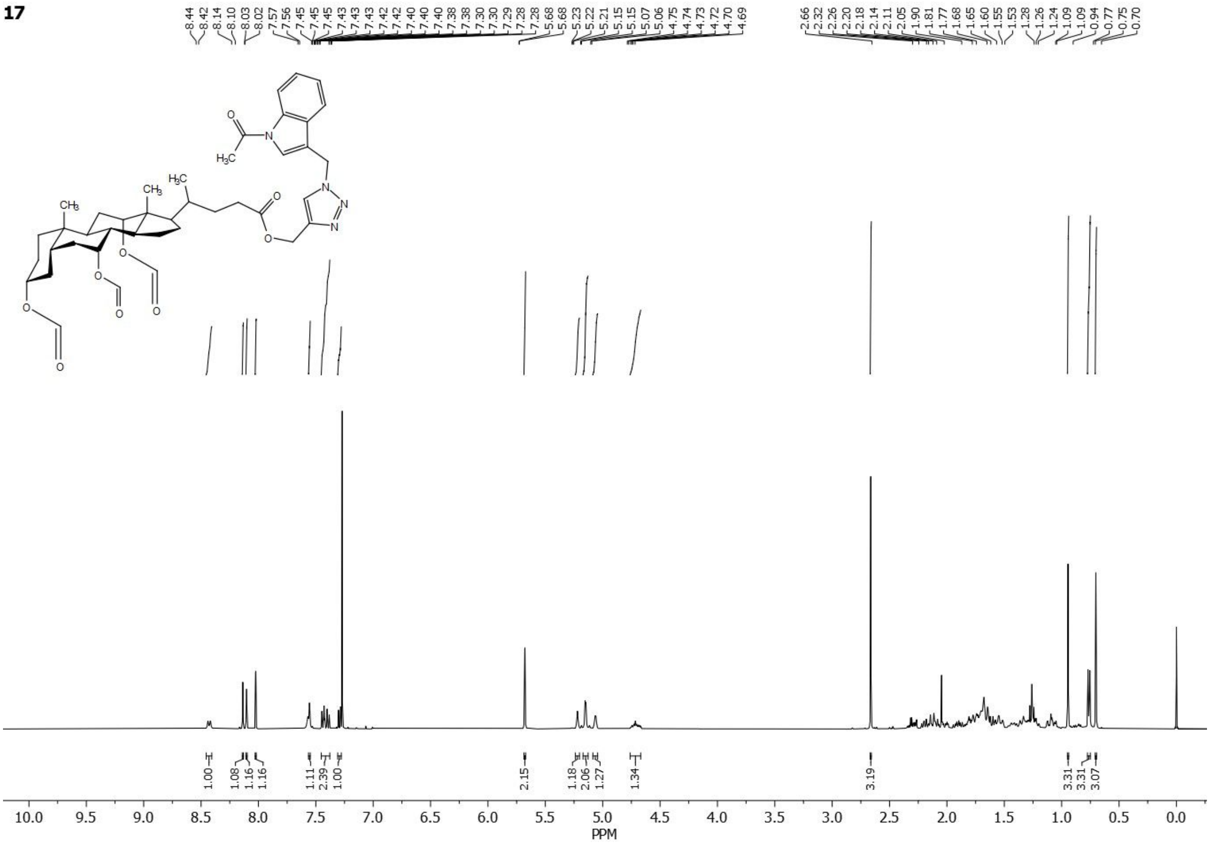

^{1}H NMR (400 MHz, $CDCl_3$) spectra of compound **17**

17

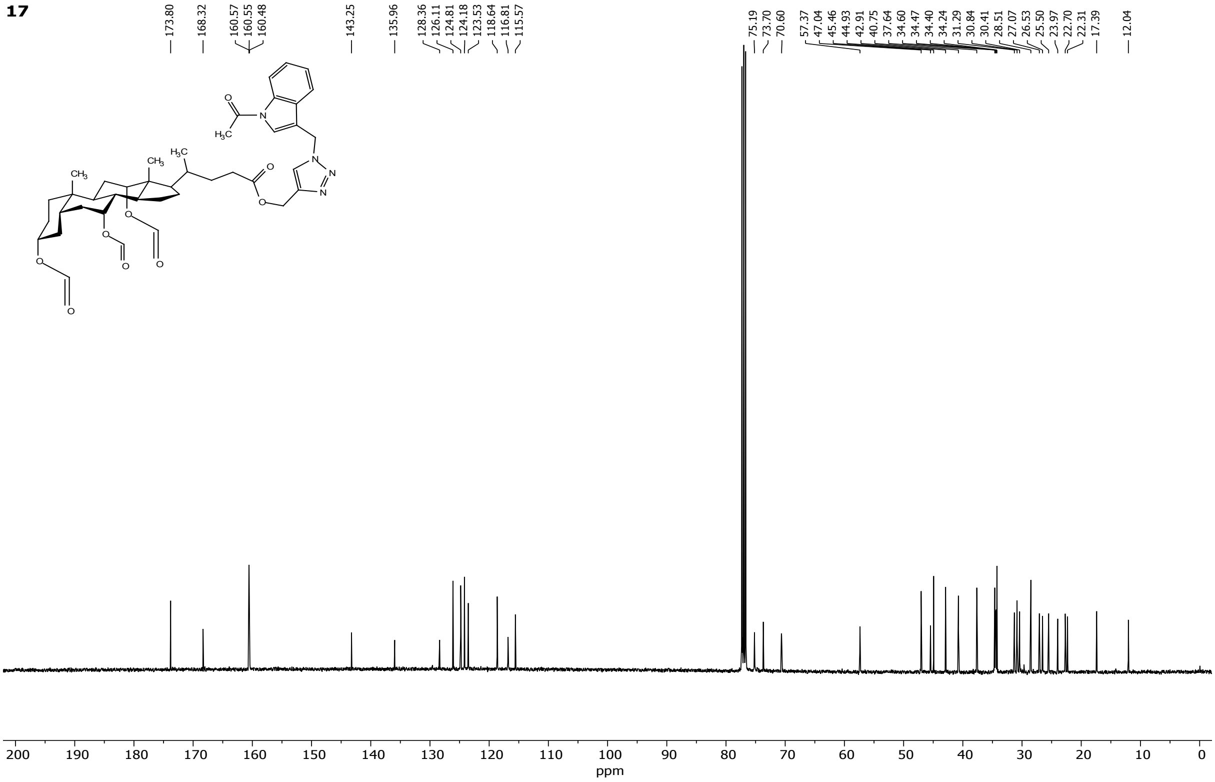

$^{13}C\{^{1}H\}$ NMR (101 MHz, $CDCl_3$) spectra of compound **17**

18

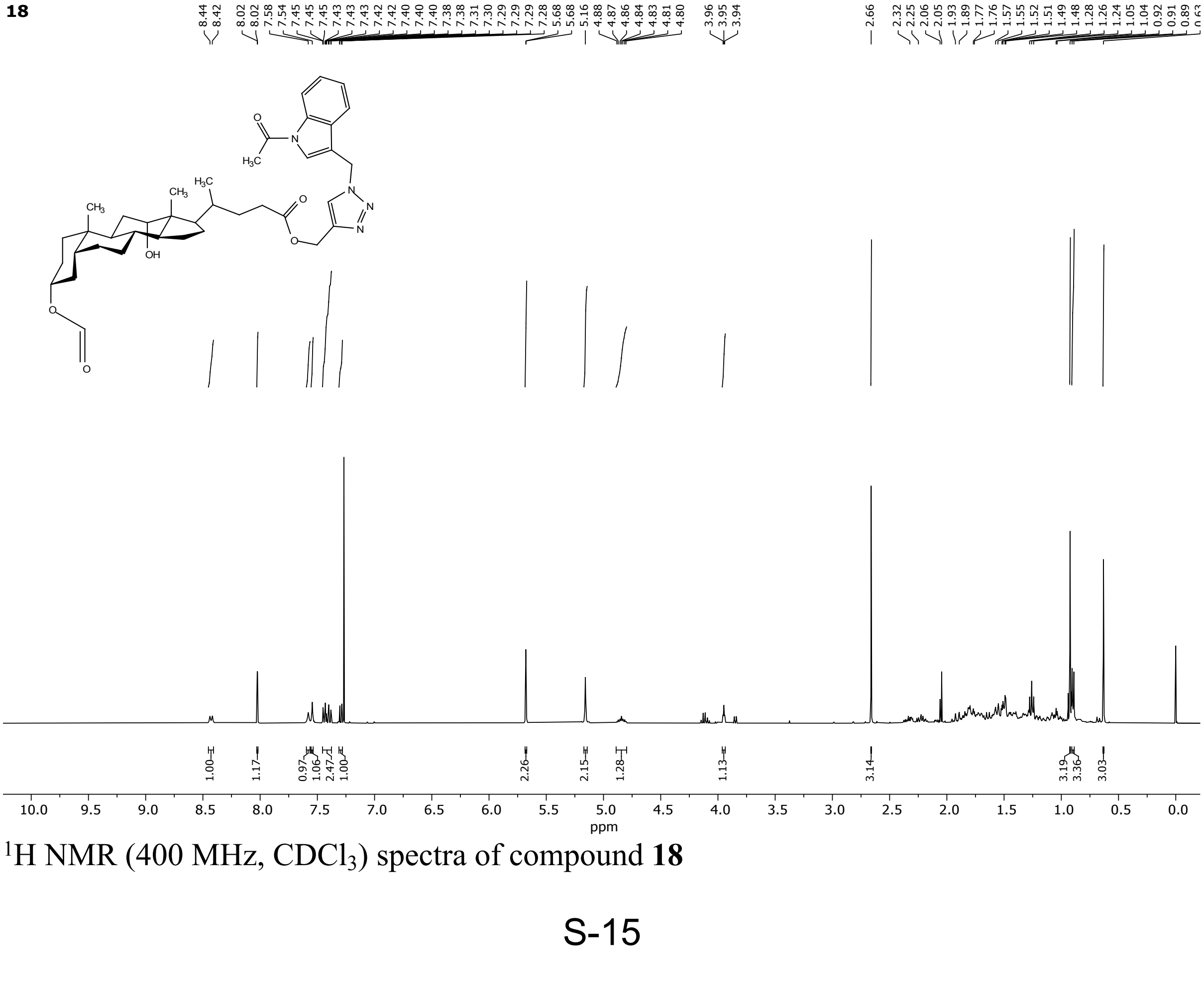

^{1}H NMR (400 MHz, $CDCl_3$) spectra of compound **18**

18

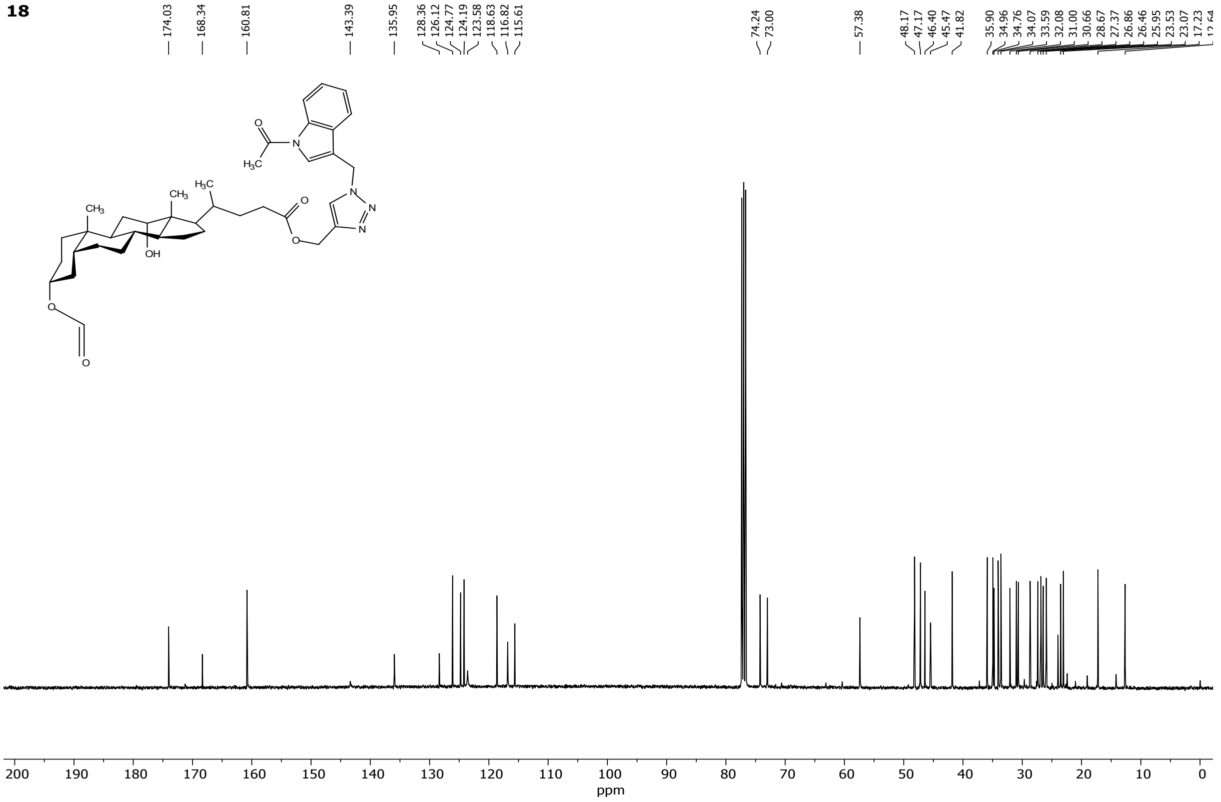

$^{13}C\{^{1}H\}$ NMR (101 MHz, $CDCl_3$) spectra of compound **18**

19

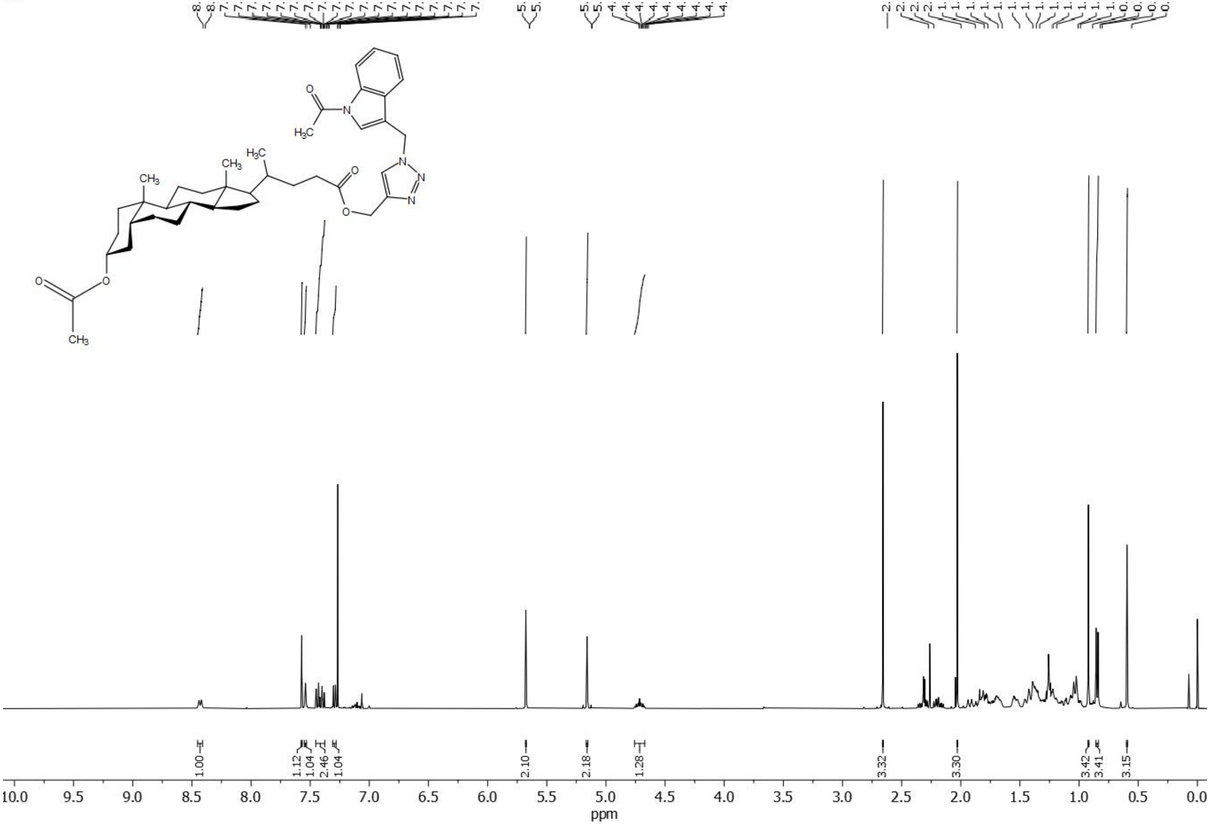

^{1}H NMR (400 MHz, $CDCl_3$) spectra of compound **19**

19

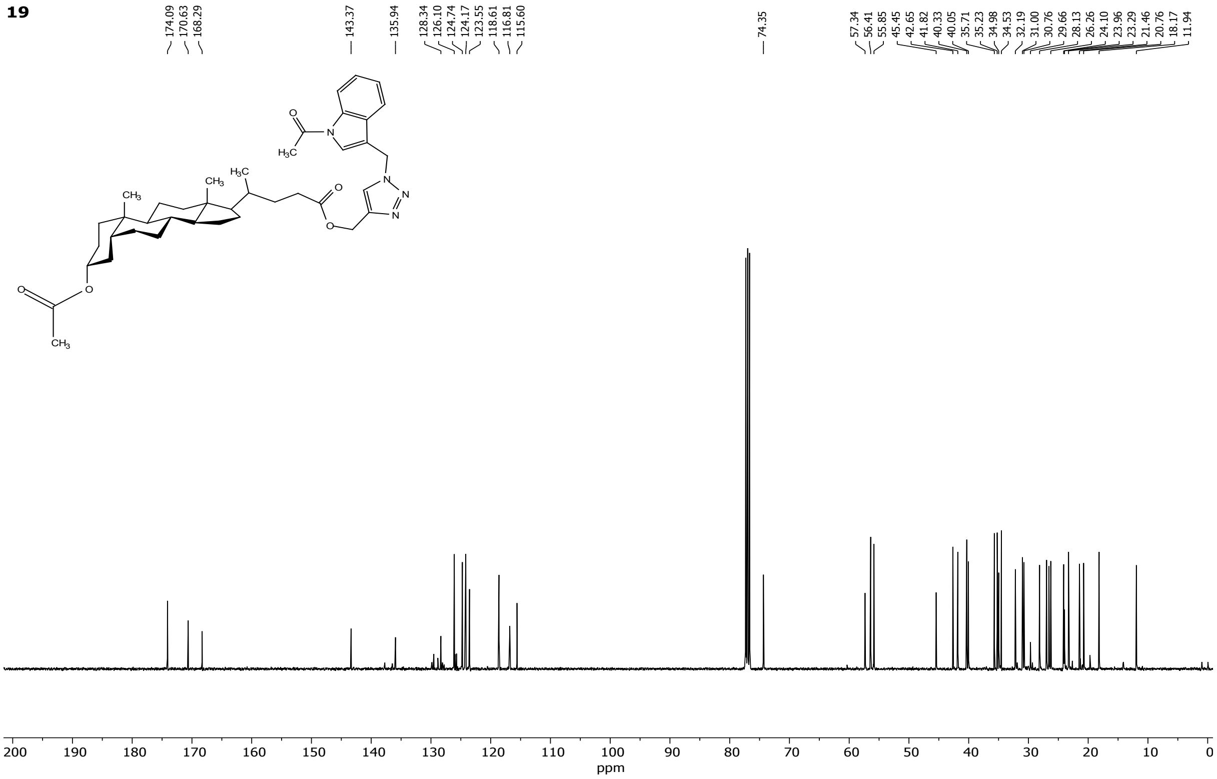

$^{13}C\{^1H\}$ NMR (101 MHz, $CDCl_3$) spectra of compound **19**

20

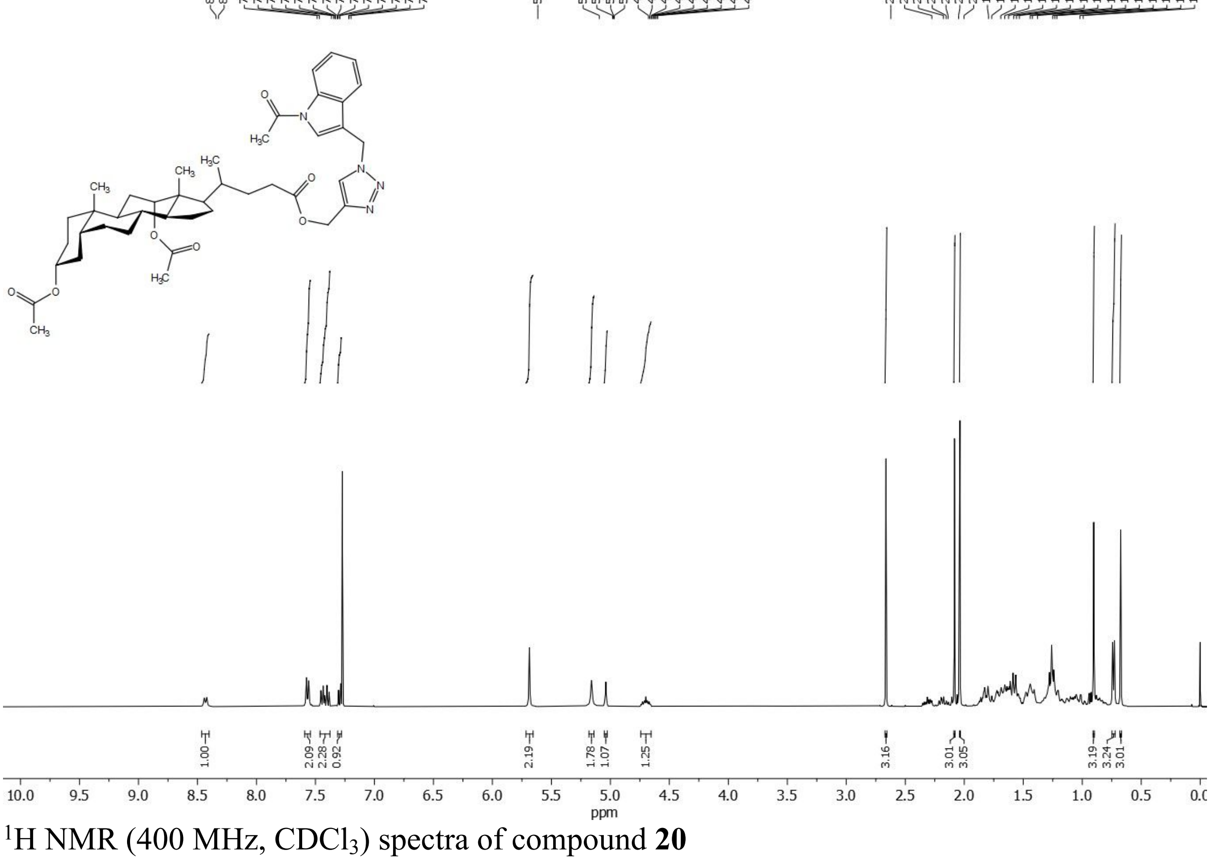

^1H NMR (400 MHz, $CDCl_3$) spectra of compound **20**

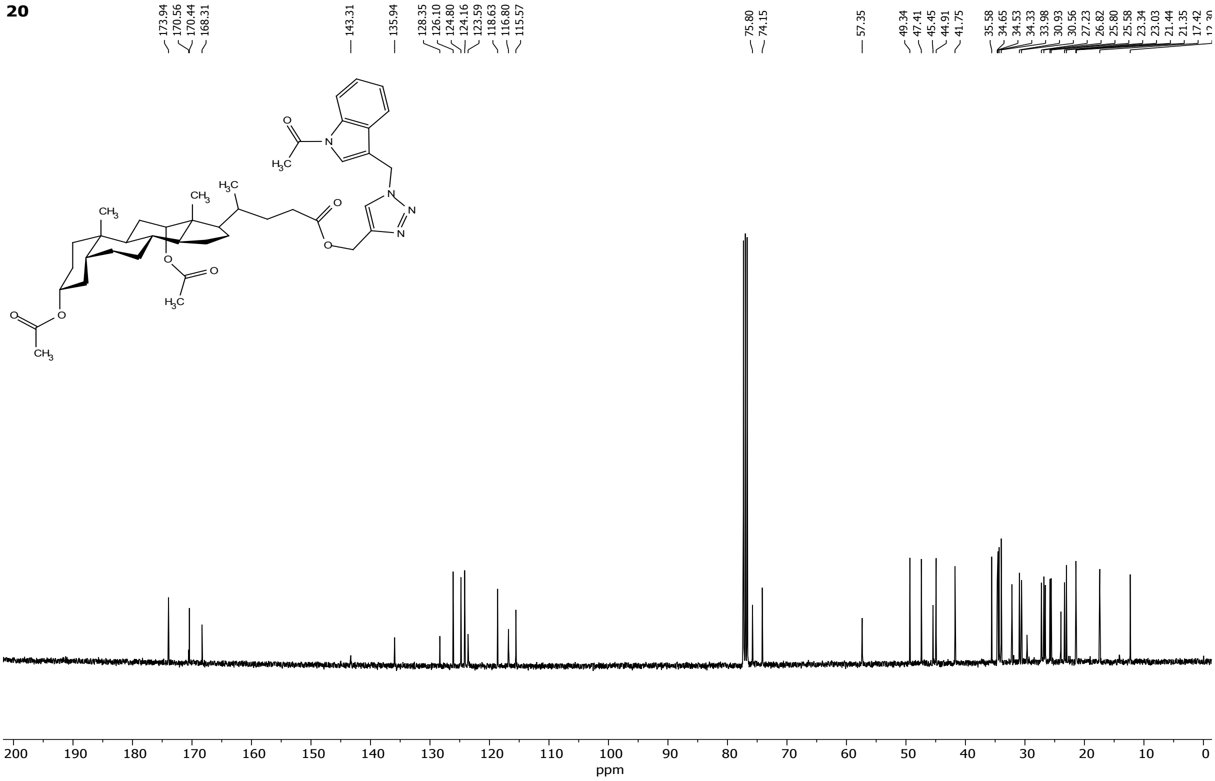

$^{13}C\{^1H\}$ NMR (101 MHz, $CDCl_3$) spectra of compound **20**

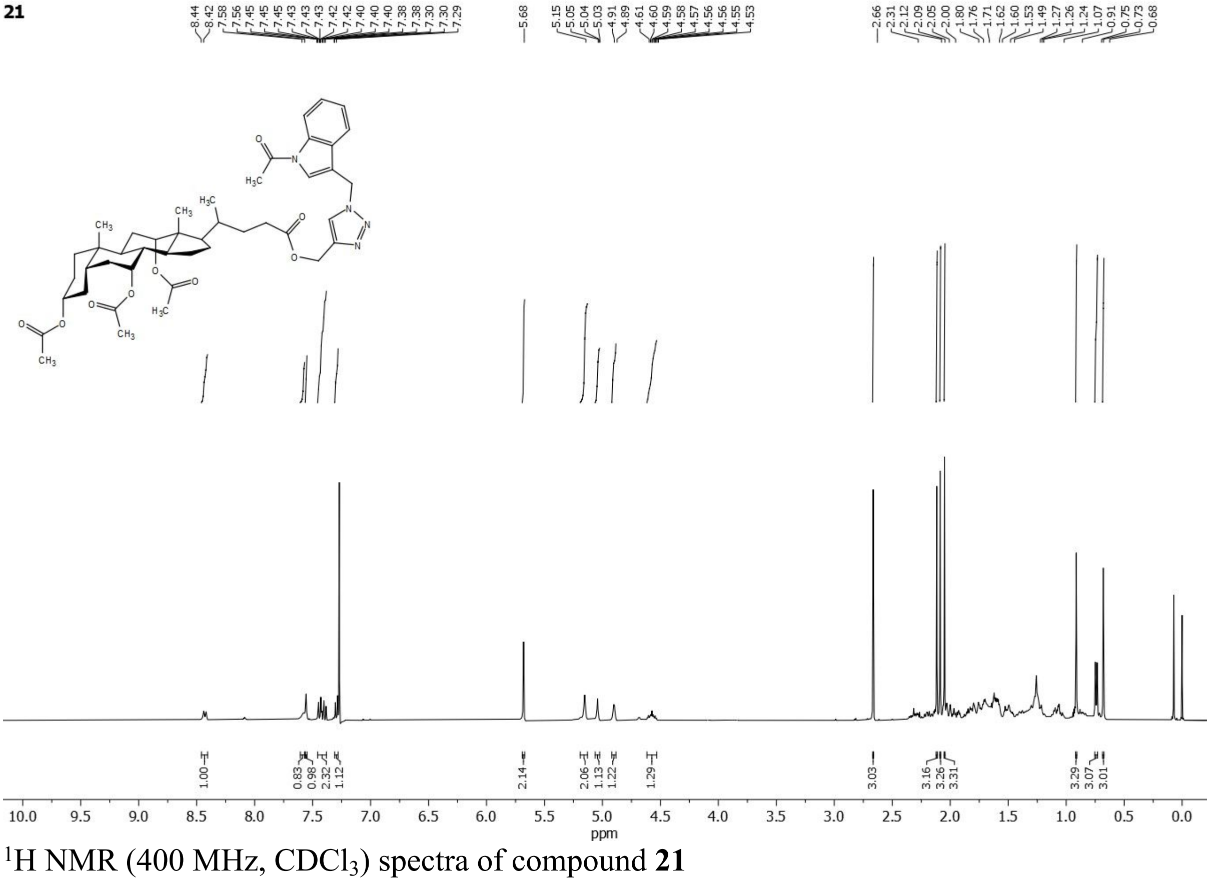

^1H NMR (400 MHz, $CDCl_3$) spectra of compound **21**

21

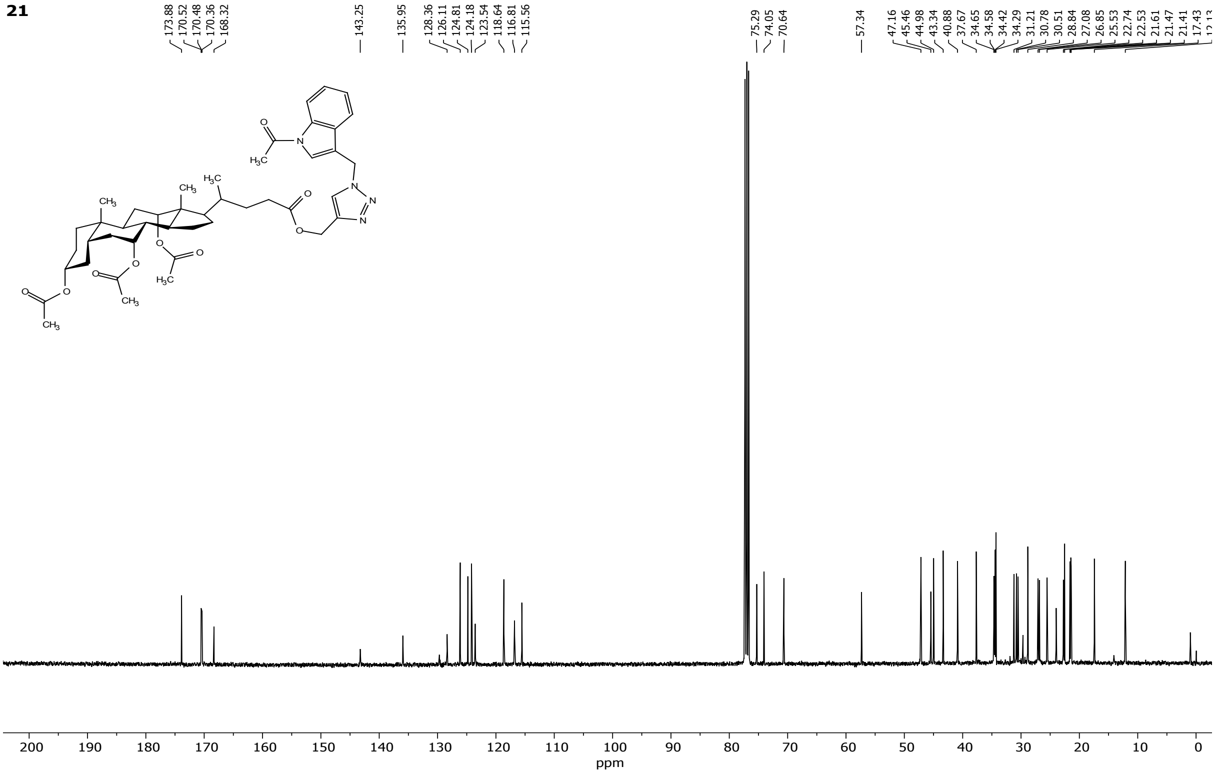

$^{13}C\{^{1}H\}$ NMR (101 MHz, $CDCl_3$) spectra of compound **21**
